# Derivatives of 6-cinnamamido-quinoline-4-carboxamide impair lysosome function and induce apoptosis

**DOI:** 10.18632/oncotarget.9348

**Published:** 2016-05-13

**Authors:** Hsiao-Hui Kuo, Rajesh Kakadiya, Yi-Chen Wu, Tsann-Long Su, Te-Chang Lee, Yi-Wen Lin, Ling-Huei Yih

**Affiliations:** ^1^ Institute of Cellular and Organismic Biology, Academia Sinica, Taipei 115, Taiwan; ^2^ Institute of Biomedical Sciences, Academia Sinica, Taipei 115, Taiwan

**Keywords:** anticancer, autophagy, cinnamamide quinolines, ERK, lysosome

## Abstract

Autophagy is a lysosomal degradative process that protects cancer cells from multiple types of stress. In this study, we synthesized a series of derivatives of 6-cinnamamido-quinoline-4-carboxamide (CiQ), and investigated their effects on the proliferation and autophagy of cancer cells *in vitro*. These derivatives effectively inhibited the proliferation of a broad spectrum of cancer cell lines. Further study revealed that CiQ derivatives may induce autophagy and result in disruption of autophagy propagation. Consequently, these derivatives triggered massive apoptosis, as evidenced by caspase-9 activation and PARP cleavage. Blockage of autophagy by depletion of autophagy related gene ATG5 or BECN1 considerably alleviated CiQ-induced cell death, indicating that autophagy may mediate CiQ-induced cell death. Furthermore, treatment with CiQ derivatives increased lysosome membrane permeability (LMP) and enhanced accumulation of ubiquitinated proteins, which collectively indicate impaired lysosome function. In addition, treatment of cells with CiQ derivatives activated extracellular signal-regulated kinase (ERK); abrogation of ERK activation, either by treating cells with U0126, an inhibitor of mitogen-activated protein/ERK kinase 1 (MEK1), or by ectopically overexpressing a dominant-negative MEK1, significantly reduced CiQ derivative-induced LMP, LC3 and p62 accumulation, and cytotoxicity. These results indicate that CiQ derivatives activate ERK and disrupt lysosome function, thereby altering autophagic flux and resulting in apoptotic cell death.

## INTRODUCTION

The contribution of the quinoline motif toward the anticancer activity of certain compounds is evident from studies of the clinically-used anticancer drugs Camptothecin, Topotecan, and Irinotecan. Quinoline derivatives not only exert their anticancer activities through targeting DNA or microtubules and inducing cell cycle arrest or apoptosis, but also inhibit angiogenesis, disrupt cell migration, and modulate nuclear receptor responsiveness [1]. It is thus anticipated that quinoline derivatives may have potential as a template for the development of anticancer agents with diverse mechanisms of action. Studies of the structure-activity relationship will enable evaluation of the anticancer activity of custom-designed quinoline derivatives.

**Figure 1:**
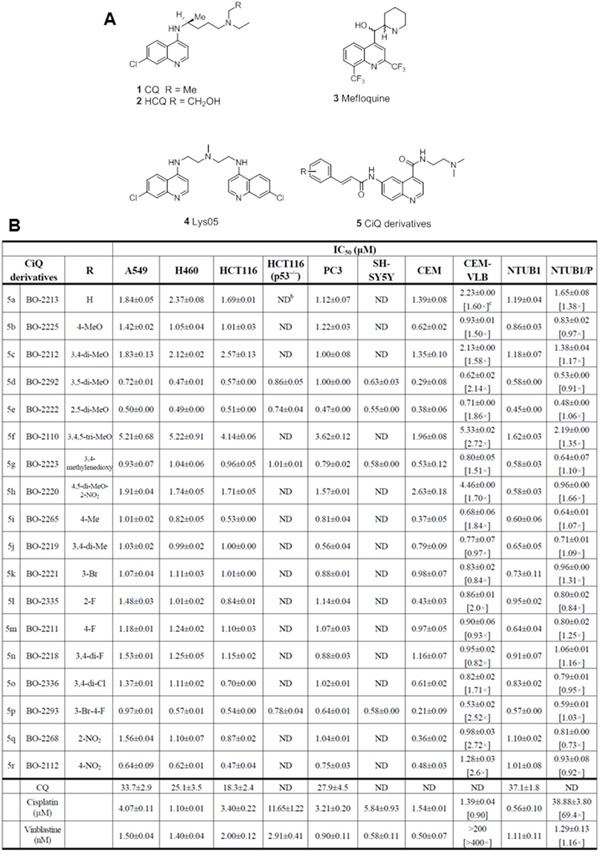
**A.** The chemical structures of selected autophagy inhibitors and CiQ. **B.** The IC50 of CiQ derivatives against the indicated human cancer cell lines. Cells were treated with each drug for 72 h, and the number of viable cells was subsequently determined using the WST-8 assay. Data represent the mean±SD of the IC50 of each compound, as determined based on the dose–effect relationships of six concentrations from three to six independent experiments. ND, not determined. Numbers in brackets are measures of cross-resistance, as determined by comparison with the corresponding IC50 value of the parent cell line.

The antimalarial chloroquine (CQ; Figure 1A compound **1**) and its analog, hydroychloroquine (HCQ; Figure 1A compound **2**), have been demonstrated to inhibit autophagy, and have been evaluated in clinical trials for cancer therapy. CQ is a weak-base lysosome-tropic quinoline, which becomes protonated when it enters the lysosome; accumulation of protonated CQ reduces the acidity of the lysosome, thereby decreasing its function [2]. Both CQ and HCQ disrupt lysosomal function, and thus inhibit autophagy [3]. Other quinoline derivatives, such as mefloquine (Figure 1, compound **3**), can also inhibit autophagy; furthermore, mefloquine exerts antileukemic effects through disrupting lysosome activity [4]. In addition, Lys05 (Figure 1, compound **4**), a dimer of 4-amino-7-chloroquinoline, exhibits enhanced lysosome accumulation and deacidification as compared with HCQ, and has significantly higher anticancer activity than CQ/HCQ in preclinical models [5]. Autophagy is a lysosome-dependent, self-catabolic process, in which cellular components are sequestered in autophagic vesicles and transported to lysosomes for proteolytic degradation [6]. Recent reports have demonstrated that autophagy is required for cell survival and proliferation following Ras-mediated transformation [7, 8]. Autophagy is also increased in tumor cells subjected to metabolic and therapeutic stress [9], and facilitates survival under such conditions [10, 11]. In addition, induction of autophagy is a key factor underlying resistance to several anticancer agents [12]. Disruption of autophagy has been reported to not only induce cancer cell death, but also sensitize cancer cells to chemotherapy-induced apoptosis [13, 14], making it a promising strategy for cancer therapy. Novel quinoline derivatives with low toxicity that can potently inhibit autophagy thus hold enormous promise as anticancer therapeutic agents.

Hybrid molecules composed of two pharmacophoric groups are often used when designing new drugs, as they may have increased potencies and/or modified selectivity profiles as compared with the corresponding single drugs [15, 16]. Cinnamamide derivatives display anticancer potential [17, 18], and can be linked with various moieties (such as phenyl, benzyl, or heterocyclic rings) while retaining their biological activity [19–21]. As such, we prepared a series of derivatives of 6-cinnamamido-quinoline-4-carboxamide (CiQ) (Figure 1A, compound **5**), which were designed as hybrid molecules consisting of a quinoline moiety and a cinnamamide core.

Water solubility is an important parameter when developing new drugs. The therapeutic application of certain drugs is limited by their poor solubility in aqueous solvents. To circumvent this issue, we synthesized CiQ derivatives in which the *N,N*-dimethylamino-ethyl hydrophilic side-chains were linked to the quinoline ring via a carboxamide linker. The tertiary amine function on the side-chain can form acid salts, yielding compounds with improved solubility in water. Here, we report that these newly synthesized CiQ derivatives disrupt lysosome function, which in turn impairs autophagy flux and induces apoptosis.

## RESULTS

### Cytotoxicity of CiQ derivatives

We synthesized 18 derivatives of CiQ (**5a-r**), and examined their effects on the proliferation of human lymphoblastic leukemia cells and several solid tumor cell lines (Figure 1B). CQ, cisplatin, and vinblastine were used as positive controls. We report that the CiQ derivatives significantly inhibited the proliferation of the tested cancer cell lines *in vitro*, with the concentrations that reduced cell growth by 50% (IC_50_) ranging from 0.3 to less than 10 μM. The structure-activity relationship which emerged from our results revealed that the substituent(s) on the phenyl ring of the cinnamamide core of CiQ do not greatly alter cytotoxicity against the tested cancer cell lines. Importantly, the IC_50_ values of the CiQ derivatives were at least 10-fold lower than that of CQ, indicating greater cytotoxicity of the derivatives. Of the tested compounds, **5d** (BO-2292), **5e** (BO-2222), **5g** (BO-2223), **5p** (BO-2293), and **5r** (BO-2112) exhibited antitumor activity against a broad spectrum of tested cell lines. We proceeded to examine the effects of these derivatives on autophagy.

### CiQ derivatives are effective against multidrug resistant and *p53* knockout cells

Acquired multidrug resistance (MDR) is a major concern for cancer chemotherapy [22]. To determine whether our CiQ derivatives are effective against MDR cells, we measured their cytotoxicity against two pairs of cell lines: (i) human lymphoblastic leukemia CCRF/CEM and its vinblastine-resistant sub-line (CCRF-CEM/VLB; exhibits over 200-fold resistance to vinblastine) [23] ; and (ii) human bladder cancer NTUB1 and its cisplatin resistant sub-line (NTUB1/P; exhibits over 60-fold resistance to cisplatin) [24]. All the tested compounds displayed relatively equal cytotoxicity within the isogenic cell line pairs (Figure 1B), indicating that the MDR cell lines possess little to no cross-resistance to the CiQ derivatives. This suggests that the tested compounds are poor substrates for membrane MDR transporters. Since *p53* status is also a major obstacle for cancer therapy [25], we examined the cytotoxic effects of CiQ derivatives on the human colon cancer cell line HCT-116 and its *p53* knockout sub-clone. The tested compounds (**5d**, **5e**, **5g**, and **5p**) had similar cytotoxic effects on both cell lines. In contrast, cisplatin was less cytotoxic against the *p53* knockout sub-line than parental HCT-116 (Figure 1B). These results indicate that our novel CiQ compounds significantly inhibit the proliferation of cancer cells with diverse origins, independent of their p53 and p-glycoprotein status.

### CiQ derivatives induce autophagy and result in disruption of autophagic flux

**Figure 2:**
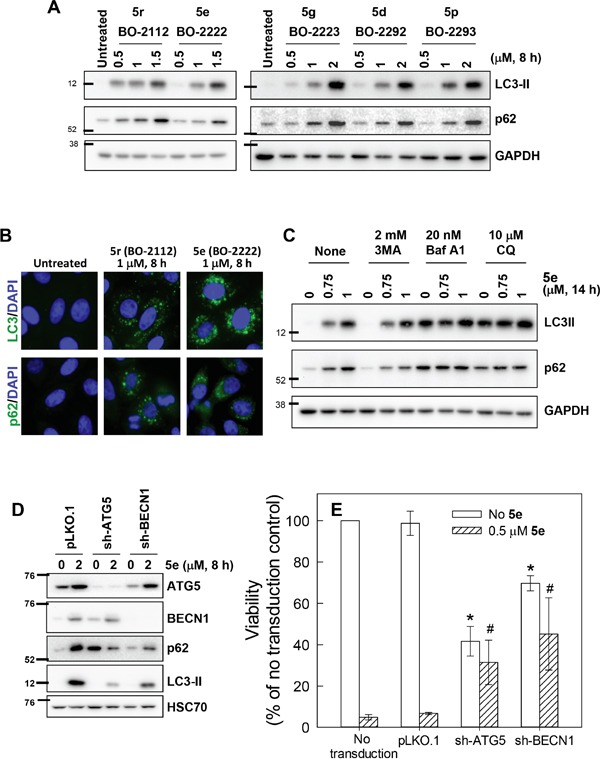
Disruption of autophagy by treatment with CiQ derivatives **A.** A549 cells were treated with the indicated concentration of CiQ derivative for 8 h, and then subjected to immunoblotting to detect cellular accumulation of LC3-II and p62. **B.** A549 cells were treated with 1 μM **5e** (BO-2222) or **5r** (BO-2112) for 8 h and then subjected to immunofluorescence staining of LC3 or p62 (green). Nuclei were counterstained with DAPI (blue). **C.** A549 cells were treated with the indicated concentration of **5e** (BO-2222) alone or together with 2 mM 3MA, 20 nM bafilomycin A1 (Baf A1), or 10 μM CQ for 14 h, and the cells were subsequently subjected to LC3-II and p62 immunoblotting. GAPDH served as a loading control. **D** and **E.** The expression of ATG5 or BECN1 was downregulated by transducing cells with virion containing the corresponding shRNA. At 72 h after transduction, cells were collected for immunoblot analysis to verify the depletion efficiency (D), or for analysis of the cytotoxicity of **5e** (E). The data represent the mean ± SD of at least three independent experiments. * indicates *p* < 0.01 as compared to untransduced and untreated cells and # indicates *p* < 0.01 as compared to cells transduced with control vector and treated with CiQ, according to Student's *t* test.

We proceeded to examine the mechanisms underlying the cytotoxic effects of **5d**, **5e**, **5g**, **5p**, and **5r**. The tested compounds neither induced γ–H2AX (a marker for DNA double strand breaks) nor altered tubulin polymerization (data not shown), but considerably induced accumulation of microtubule-associated protein-1 light chain 3-II (LC3-II, the cleaved form of LC3) (Figure 2A). Immunofluorescence staining revealed that all of the treated cells contained LC3 punctae, but untreated cells exhibited a weaker and diffused LC3 distribution pattern (Figure 2B, upper panel), indicating that CiQ derivatives may induce autophagy and/or block autophagic flux. A second marker of autophagy, the adaptor/scaffold protein p62/sequestosome-1 (p62), was significantly increased after CiQ treatment (Figure 2A), and was present in punctae or aggregates (Figure 2B, lower panel). As the steady-state level of p62 is regulated by autophagy, and accumulation or aggregation of p62 is considered a marker of defective autophagic degradation [26], our results suggest that treatment with CiQ derivatives may also impair autophagic flux. The **5e**-induced accumulation of p62, but not LC3-II (Figure 2C), was slightly reduced by co-treatment with 3-methyladenine (3MA, a class III PI3K inhibitor that inhibits the initiation of autophagy), suggesting that **5e** might induce autophagy therefore blockage of autophagy initiation can reduce **5e**–induced p62 accumulation. Bafilomycin A1 or CQ (inhibitors that block the fusion of autophagosomes and lysosomes) alone significantly induced accumulation of both LC3-II and p62 (Figure 2C), but did not further enhance LC3-II and p62 accumulation when applied together with **5e**; this suggests that **5e** may also block autophagic degradation, thereby preventing any further enhancement of **5e**–induced p62 accumulation by bafilomycin A1 or CQ. These observations indicate that CiQ derivative-induced defects may induce autophagy and subsequently block propagation of autophagy at a later stage.

To verify the role of autophagy in CiQ cytotoxicity, we downregulated the expression of autophagy-related gene 5 (ATG5) or BECN1 in A549 cells through the use of specific shRNAs. CiQ (**5e**) treatment resulted in increased expression of ATG5 and BECN1, and this was accompanied by significant accumulation of LC3-II and p62 (Figure 2D). Depletion of ATG5 or BECN1 substantially reduced CiQ-induced accumulation of LC3-II (Figure 2D), indicating that autophagy was blocked. Depletion of ATG5 significantly induced p62 accumulation (Figure 2D), suggesting that ATG5 might also participate in the processing of p62. Nonetheless, blockage of autophagy by depletion of ATG5 or BECN1 also significantly prevent **5e**–induced accumulation of p62 (Figure 2D). In addition, depletion of ATG5 or BECN1 reduced cell viability, but considerably alleviated CiQ-induced cell death, as compared to cells transduced with control shRNA (Figure 2E). These results indicate that autophagy is indispensable for proliferation of A549 cells and may mediate the cytotoxicity of CiQ derivatives.

### CiQ derivatives induce apoptosis

**Figure 3:**
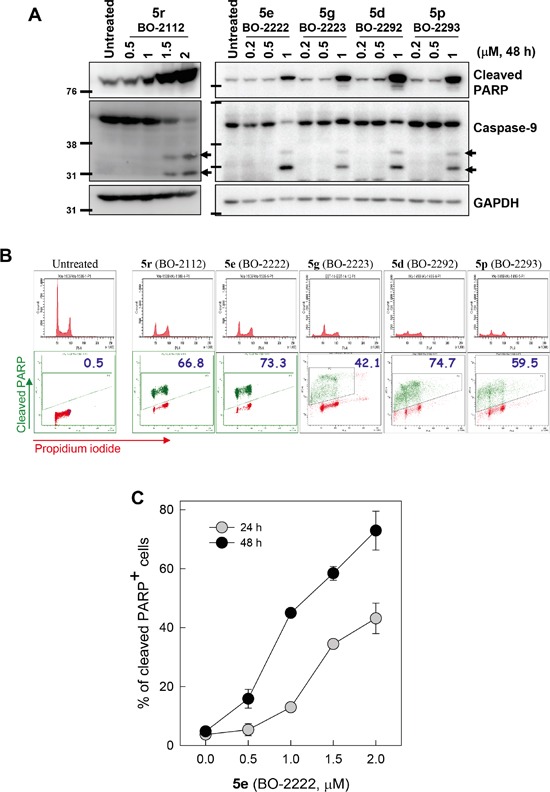
Induction of apoptosis by treatment with CiQ derivatives **A.** A549 cells were treated with the indicated CiQ derivative for 48 h and then subjected to immunoblotting to detect cleavage of PARP (cPARP) and caspase-9. **B.** Cell cycle distribution of cPARP-positive cells. A549 cells were treated with the indicated CiQ derivative for 48 h, and then subjected to flow cytometric analysis of PARP cleavage. Cellular DNA was counterstained with propidium iodide. In the lower panel, the green dots indicate cleaved PARP-positive cells and the numbers indicate the percentage of cleaved PARP-positive cells. **C.** A549 cells were treated with **5e** (BO-2222) for 24 or 48 h, and PARP cleavage was subsequently analyzed by flow cytometry. The percentages of cells with cleaved PARP were determined. The data represent the mean ± SD for three independent experiments.

Subsequent to disrupting autophagy, CiQ treatment induced apoptosis, as demonstrated by immunoblot analysis of cleaved caspase-9 and poly(ADP-ribose) polymerase (PARP) (Figure 3A). To determine the stage of the cell cycle at which apoptosis was initiated, we treated cells with 2 μM of the indicated CiQ derivative for 48 h, and then subjected the cells to flow cytometry to simultaneously detect cleaved PARP and DNA content. This revealed that CiQ derivative-induced apoptosis was independent of cell cycle stage (Figure 3B). Flow cytometric analysis also revealed that compound **5e** induced PARP cleavage in a concentration- and time-dependent manner (Figure 3C). These results indicate that CiQ cytotoxicity mainly arises from induction of apoptosis.

### CiQ derivatives alter lysosome function

**Figure 4:**
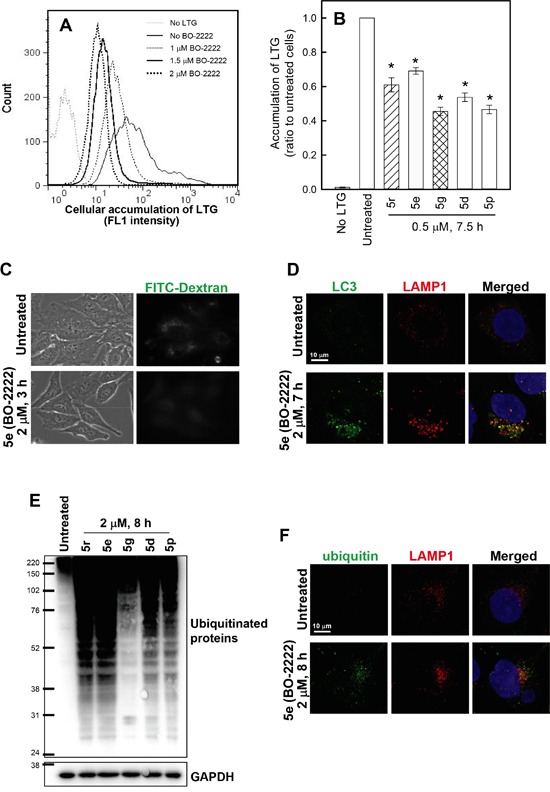
Induction of lysosome dysfunction by treatment with CiQ derivatives **A** and **B.** CiQ derivative treatment reduced the accumulation of LTG. A549 cells were untreated or treated for 7 h with 1–2 μM **5e** (BO-2222) (A) or with 1 μM of the indicated CiQ (B), and then loaded with LTG by incubation in media containing 100 nM LTG for 30 min. Following thorough washout of excess LTG, cellular accumulation of LTG was analyzed by flow cytometry at FL1. The FL1 spectrum in (A) indicates cellular accumulation of LTG. Cells unlabeled with LTG were used as a negative control. (B) The ratio of the mean intensity of LTG at FL1 in cells treated with test compounds to that in untreated cells. The data represent the mean ± SD for three independent experiments. * indicates *p* < 0.01 as compared to untreated cells. **C.** CiQ derivatives induced the release of FITC-dextran from lysosomes. A549 cells were incubated in media containing 1 mg/ml FITC-dextran for 14 h. After thorough washout of free FITC-dextran, the FITC-dextran-loaded cells were subsequently treated with vehicle or 2 μM **5e** (BO-2222) for 3 h. The cells were monitored under an inverted fluorescence microscope. **D. 5e** (BO-2222)-induced LC3 punctae colocalized with LAMP1. A549 cells were untreated or treated with 2 μM **5e** (BO-2222) for 7 h, and then immunostained for LC3 (green) and LAMP1 (red). Nuclei were counterstained with DAPI (blue). **E** and **F.** Treatment with CiQ derivatives induced accumulation of ubiquitinated proteins. A549 cells were treated with 2 μM of the indicated CiQ derivative for 8 h, and ubiquitinated proteins were subsequently detected by immunoblotting (E) or immunofluorescence staining (E, green). For immunofluorescence staining, cells were co-immunostained with LAMP1 (red) to locate lysosomes, and nuclei were counterstained with DAPI (blue).

Degradation of p62 is regulated by the digestive activity of lysosomes [26]. Therefore, we proceeded to examine the effects of CiQ derivatives on lysosome function by using LysoTracker® Green (LTG), a dye that accumulates in acidic compartments such as lysosomes, to evaluate the integrity of the lysosome compartment. Cells were treated with vehicle or **5d**, **5e**, **5g**, **5p**, or **5r** for 7 h, and then loaded with LTG for 30 min. Flow cytometric analysis showed that **5e** (BO-2222) treatment reduced cellular accumulation of LTG in a concentration-dependent manner (Figure 4A). Other CiQ derivatives also significantly reduced cellular LTG accumulation (Figure 4B), suggesting that these derivatives may prevent LTG retention by deacidifying the lysosome and enhancing lysosome membrane permeability (LMP). To confirm this hypothesis, we monitored cellular endocytic uptake and release of a non-digestible fluid-phase substrate, FITC-dextran. After 14 h of incubation in media containing 1 mg/ml FITC-dextran (10 kDa), cells exhibited a punctate pattern of fluorescent dextran particles (Figure 4C, upper panel); these findings are indicative of uptake and accumulation of FITC-dextran in the lysosomes, even after 3 h of washout. However, in the presence of **5e**, the punctate pattern of FITC-dextran became diffused (Figure 4C, lower panel), indicating the release of FITC-dextran from the lysosomal compartment. This finding thus confirms that CiQ derivatives enhance LMP, and may disrupt lysosome integrity. We next examined whether lysosome disruption contributes to CiQ derivative-induced defective autophagy, by examining cellular localization of LC3 and lysosome associated membrane protein 1 (LAMP1). The results showed that LC3 punctae mainly colocalized with LAMP1 in **5e**-treated cells (Figure 4D), indicating that **5e** did not inhibit fusion of autophagosomes and lysosomes.

Ubiquitin serves as a common cargo recognition signal for the recruitment of substrates into autophagosomes, and then to autolysosomes for degradation [27, 28]. We examined whether autophagic clearance of ubiquitinated proteins was altered by treatment with CiQ derivatives, by performing immunoblotting and immunofluorescence staining with an antibody against ubiquitin. We report that ubiquitinated proteins were significantly increased in cells treated for 8 h with the test compounds (Figure 4E), and were co-localized with LAMP1 in punctae structures in **5e**-treated cells (Figure 4F, lower panel), indicating that their degradation in lysosomes may be blocked. These results indicate that the CiQ derivatives inhibited lysosomal degradative activity.

### Abrogation of ERK activation reduces CiQ-induced autophagy, LMP, and cell death

**Figure 5:**
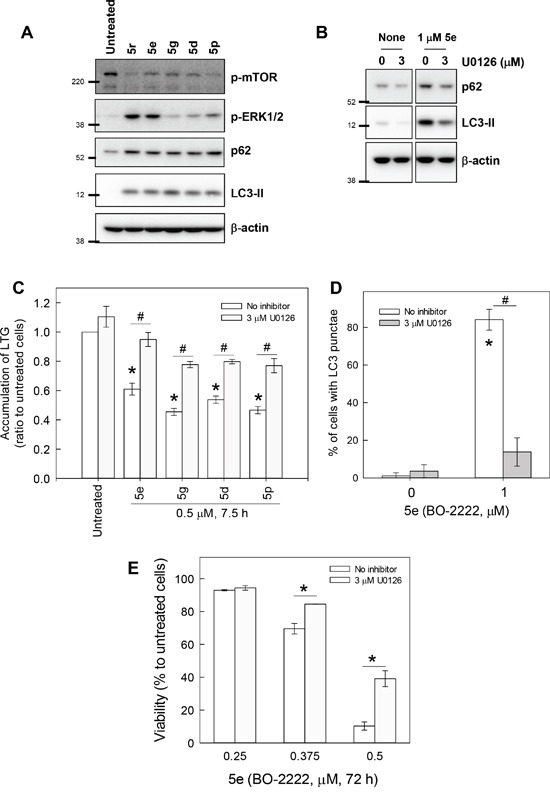
CiQ derivatives disrupted lysosome function and blocked autophagy in an ERK-dependent manner **A.** CiQ derivative treatment induced phosphorylation of ERK. A549 cells were treated with 2 μM of the indicated CiQ derivative for 8 h, and then subjected to immunoblot with antibodies against the indicated protein. **B.** U0126 reduced **5e** (BO-2222)-induced accumulation of LC3-II and p62. A549 cells were pre-treated with 3 μM U0126 for 14 h and then co-treated with 1 μM **5e** for another 8 h. **C.** U0126 ameliorated CiQ-induced LMP. A549 cells were pre-treated with 3 μM U0126 for 14 h and then co-treated with 0.5 μM of the indicated CiQ derivative for 7 h. The cells were then loaded with LTG, and LTG accumulation was analyzed. **D.** U0126 reduced **5e**-induced LC3 punctae formation. A549 cells were pre-treated with 3 μM U0126 for 14 h and then co-treated with 1 μM **5e** for 8 h. LC3 punctae were detected by immunofluorescence staining. At least 300 cells were examined under a fluorescence microscope for each experiment. **E.** U0126 reduced the cytotoxicity of **5e**. A549 cells were treated with the indicated concentration of **5e** alone or together with 3 μM U0126 for 72 h. Cell viability was determined using the WST-8 assay. The data represent the mean ± SD of at least three independent experiments. * indicates *p* < 0.01 as compared to untreated cells and # indicates *p* < 0.01 as compared to CiQ treatment alone, according to Student's *t* test.

**Figure 6:**
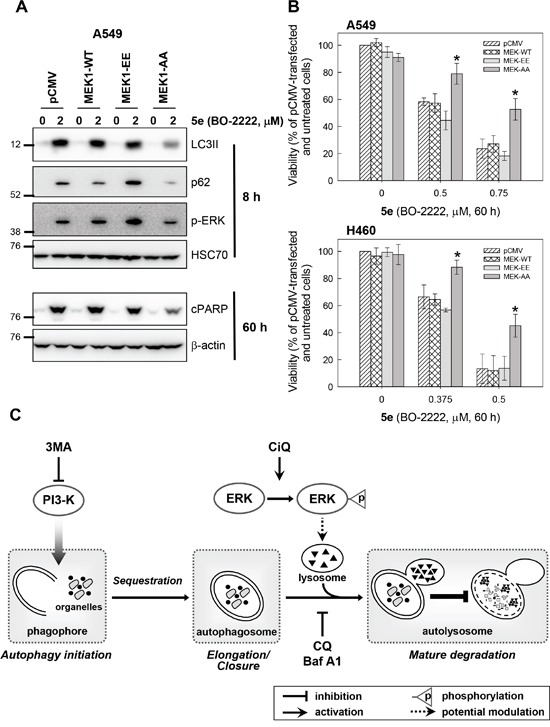
**A** and **B.** Ectopic overexpression of a dominant-negative MEK1 ameliorated **5e**-induced phosphorylation of ERK, accumulation of LC3-II and p62, and cytotoxicity. A549 or H460 cells were transfected with the indicated MEK expression construct, and at 24 h after transfection were treated with 2 μM **5e** for 8 h or 60 h (A), or with the indicated concentration of **5e** for 60 h (B). Immunoblotting was used to detect the indicated protein (A). Cell viability was determined using the WST-8 assay (B). * indicates *p* < 0.01 as compared to empty vector (pCMV)-transfected cells subjected to the same CiQ treatment. **C.** A simplified model depicting how CiQ disrupts autophagy. The three main steps in the autophagic pathway are shown. The formation of the autophagosome depends on the initiation and/or nucleation of a specific membrane structure, known as the phagophore. A portion of the cytoplasm, including organelles, is sequestered in the phagophore. The autophagosome is formed following elongation and closure of the phagophore. Finally, the outer membrane of the autophagosome fuses with the lysosome, and the internal material is degraded within the autolysosome. Autophagy can be inhibited at early stages using 3MA (a PI3K inhibitor) to block initiation, or at late stages using CQ (a lysosomotropic agent) or bafilomycin A1 (Baf A1, an ATPase inhibitor) to block the fusion of autophagosomes and lysosomes. We report here that CiQ derivatives activate ERK and impair lysosome function, which disrupts the degradative activity of autolysosomes (as indicated by the bar-headed line).

We next attempted to delineate the mechanism by which CiQ derivatives alter the function of lysosomes and autophagy, by examining their effects on the mTOR, PI3K/AKT, and MAPK signaling pathways. As shown in Figure 5A, treatment with **5d**, **5e**, **5g**, **5p**, or **5r** not only induced accumulation of LC3-II and p62, but also significantly reduced phosphorylated mTOR and increased phosphorylated extracellular signal-regulated kinase (ERK). Only minor effects on PI3K/AKT, GSK3, and JNK were observed (data not shown). mTOR is a well-conserved negative regulator of autophagy initiation [29], and therefore the observed decrease in its phosphorylation was not unexpected. To determine the role of CiQ derivative-induced ERK activation, we used U0126, an inhibitor of mitogen-activated protein/ERK kinase 1 (MEK1), to prevent ERK activation. Co-treatment of cells with the test compounds and U0126 significantly reduced CiQ-induced accumulation of LC3-II, p62 (Figure 5B), and LMP (Figure 5C), as well as LC3 punctae formation (Figure 5D) and cytotoxicity (Figure 5E). In addition, **5e**-induced phosphorylation of ERK, accumulation of LC3-II and p62, cleavage of PARP (Figure 6A), and cytotoxicity (Figure 6B) were decreased by ectopic overexpression of a dominant-negative MEK1 mutant (MEK1-AA), unaffected by overexpression of wild type MEK1 (MEK1-WT), and slightly enhanced by overexpression of constitutively active MEK1 (MEK1-EE). These results indicate that CiQ derivatives may alter lysosome function and disrupt autophagy through activation of ERK.

## DISCUSSION

Exploiting vulnerabilities in the intracellular signaling pathways of tumor cells is a key strategy for the development of new drugs. Here, we describe the design and synthesis of a series of hybrid CiQ derivatives. These compounds display potent *in vitro* cytotoxicity against various human solid tumors, independent of the cellular status of p53 or p-glycoproteins, and of tissue type. The inhibitory effects of these compounds appear to arise primarily from their ability to alter lysosome function and disrupt autophagy.

Autophagy can be disrupted at several stages, including formation of the autophagosome, vesicle fusion with lysosomes, and degradation of autolysosome contents [30]. It has been demonstrated that disruption of microtubule dynamics inhibits autophagosome trafficking, thereby preventing the maturation of autophagosomes [31–33]. Derivatives of phenylcinnamide have been demonstrated to induce cancer cell death through inhibition of tubulin polymerization and the resulting induction of mitotic defects [21]. However, our CiQ derivatives, which bear a phenylcinnamide moiety, did not affect microtubule polymerization or induce mitotic arrest in treated cells (data not shown), suggesting that their inhibitory effects on autophagy are not due to microtubule disruption. Instead, our results demonstrated that these agents impair the lysosomal accumulation of LTG and 10-kDa fluorescent dextran, indicating that CiQ may deacidify the lysosome, induce LMP, and disrupt the integrity of lysosomes.

The lysosome is a membrane-enclosed organelle containing many hydrolytic enzymes essential for the turnover of macromolecules [34]. Induction of LMP causes the release of cathepsins and other hydrolases from the lysosomal lumen to the cytosol [35]. However, we did not observe a significant redistribution of cathepsin B from the lysosomes to the cytosol (data not shown). These results suggest that CiQ derivatives may not completely destabilize the lysosome by inducing massive lysosome breakdown, and as such, only smaller molecules may be released into the cytoplasm. In addition, the deleterious effects of CiQ derivatives on lysosomes did not affect the colocalization of CiQ-induced LC3 punctae and LAMP1, suggesting that CiQ may not disrupt the fusion of autophagosomes and lysosomes. However, we observed that ubiquitinated proteins were considerably increased in the lysosomes of cells treated with CiQ derivatives. Misfolded proteins and/or damaged organelles are ubiquitinated, targeting them for autophagic clearance by p62-mediated autophagosomal engulfment [27, 28]. Disruption of autophagy by CQ has been shown to induce accumulation of ubiquitinated proteins in the lysosome [36]. Thus, CiQ derivatives may inhibit the lysosomal degradative activity required for autophagic clearance.

We also report that CiQ-induced LMP can be rescued by co-treatment with U0126, indicating that ERK activation may contribute to CiQ-induced lysosome dysfunction. Accumulating evidence indicates that impairment of autophagy through disruption of lysosome function can be triggered by drug-induced activation of ERK [37–39]. The precise role of ERK in autophagy has not been clearly defined. Oncogenic activation of Ras, the upstream activator of ERK, has been demonstrated to induce autophagic vacuolation and to reduce autophagic degradation [40]. Sustained activation of the ERK pathway has been shown to induce autophagic vacuolation through disruption of the maturation of autophagosomes into functional autolysosomes [41]. Although our results suggest that the fusion of the autophagosome and the lysosome is not inhibited by CiQ treatment, the maturation of functional autolysosomes may be altered by CiQ-induced ERK activation. This was confirmed by our finding that prevention of ERK activation (whether by U0126 treatment or ectopic expression of dominant-negative MEK1) ameliorates CiQ-induced LMP and LC3 punctae formation, and significantly reduces accumulation of p62 (the steady-state level of which is regulated by autophagy). Activation of the ERK pathway has also been reported to impair autophagy by promoting degradation of FOXO1 [42], a protein involved in the dynamic control of autophagy [43]. These reports support our hypothesis that CiQ derivatives disrupt lysosome function via ERK activation, thereby blocking autophagy. CQ is known to induce deacidification of the lysosome, and to inhibit autophagy at the fusion and lysosomal degradation steps. Our results show that CiQ also induces deacidification of the lysosome, but is likely to inhibit lysosomal degradation without affecting the fusion of autophagosomes and lysosomes. A simplified model for the roles of CiQ, CQ, and 3MA is shown in Figure 6C.

In summary, we have synthesized a series of new CiQ derivatives which can impair lysosomal function and block autophagy, and consequently induce apoptosis. Of these derivatives, compound **5e** (BO-2222) is significantly more cytotoxic than CQ to the tested cancer cell lines. This suggests that **5e** may have potential for further development as a potent inhibitor of autophagy.

## MATERIALS AND METHODS

### Synthesis of CiQ derivatives

A total of 18 CiQ derivatives were synthesized. Detailed experimental procedures for CiQ synthesis are provided in the Supplementary Information.

### Cell culture

The human lymphoblastic leukemia cell line CCRF-CEM and its vinblastine-resistant sub-line CCRF-CEM/VLB were maintained in RPMI1640 supplemented with 10% fetal bovine serum (Invitrogen), 0.2% sodium bicarbonate, 100 U/ml of penicillin, and 100 μg/ml of streptomycin, at 37°C in a humidified incubator in air and 5% CO_2_. The resistant line was routinely cultured in the presence of 100 nM vinblastine (Sigma, St. Louis, MO). A549 (lung adenocarcinoma), H460 (large-cell lung carcinoma), PC3 (prostate adenocarcinoma), and SH-SY5Y (neuroblastoma) were obtained from the American Type Culture Collection (Manassas, VA), and were routinely maintained in Dulbecco's modified Eagle's media (Invitrogen) supplemented with 10% fetal bovine serum (Invitrogen), 0.37% sodium bicarbonate, 100 U/ml of penicillin, and 100 μg/ml of streptomycin, at 37°C in a humidified incubator in air and 10% CO_2_. HCT116 (colon carcinoma) and the corresponding p53 knockout clone [44] were cultured in McCoy's 5A supplemented with 10% fetal bovine serum (Invitrogen), 0.2% sodium bicarbonate, 100 U/ml of penicillin, and 100 μg/ml of streptomycin, at 37°C in a humidified incubator in air and 5% CO_2_. The human bladder urothelial carcinoma cell line NTUB1 and the cisplatin-resistant sub-line NTUB1/P (generous gifts from Dr. Yeong-Shiau Pu (Department of Urology, National Taiwan University Hospital, Taipei, Taiwan)) [45] were cultured in RPMI1640 supplemented with 10% fetal bovine serum (Invitrogen), 0.2% sodium bicarbonate, 100 U/ml of penicillin, and 100 μg/ml of streptomycin, at 37°C in a humidified incubator in air and 5% CO_2_. The cells were passaged twice per week.

### Cytotoxicity assay

The cytotoxicity of the test compounds was determined by counting viable cell numbers with methylthiazol tetrazolium (WST-8) (Cell Count Kit 8; Dojindo Molecular Technologies, Inc.), as described previously [46]. The compound concentration that reduced cell growth by 50% (IC_50_) was determined based on the dose–effect relationships of six concentrations from three to six independent experiments, using GraphPad PRISM v5.0 (GraphPad, San Diego, CA).

### Depletion of ATG5 or BECN1

Depletion of each protein was achieved by transducing cells with VSV-G-pseudotyped lentivirus-based shRNA. ATG5-specific shRNA (TRCN151963) and BECN1-specific shRNA (TRCN299864) were obtained from the National RNAi Core Facility Platform (Institute of Molecular Biology/Genomic Research Center, Academia Sinica, Taipei, Taiwan). Cells were transduced with shRNA-containing virus (multiplicity of infection = 3) in growth medium supplemented with 10 μg/ml polybrene. At 72 h post-transduction, cells were subjected to cytotoxicity and immunoblotting assays.

### Immunoblots

Immunoblotting of cultured cell extracts was performed as described [47]. The following antibodies were used: rabbit antibodies against human caspase-9 (#9502), cleaved PARP (#9541), LC3 (#2775), phospho-ERK1/2 (#9101), phospho-mTOR (S2448, #5536), SQSTM1/p62 (#8025), and ubiquitin (#3936), from Cell Signaling Technology; mouse antibodies against human HSC70 (sc-7298), and LAMP1 (sc-18821), from Santa Cruz Biotechnology; rabbit anti-GAPDH (GTX100118) from GeneTex; and mouse anti-β-actin (MAB1501) from Chemicon. All antibodies were diluted in 5% skimmed milk prepared in PBS containing 0.2% Tween 20 (0.2% PBST). β-actin, GAPDH, or HSC70 was used as a loading control.

### Immunofluorescence staining

Cells grown on cover slips were treated as indicated, washed twice with PBS, and fixed *in situ* with 90% methanol at −20°C for 15 min. The cells were washed three more times with PBS, and co-immunostained for 1 h at room temperature with anti-LAMP1 and anti-LC3, anti-p62, or anti-ubiquitin. Non-bound antibodies were removed by extensive washing with 0.1% PBST. Cells were subsequently incubated for 1 h with Alexa 488-coupled anti-rabbit immunoglobulin and Alexa-633-coupled anti-mouse immunoglobulin antibodies (Invitrogen Life Technology), while nuclei were simultaneously counterstained with 0.1 μg/mL 4,6-diamino-2-phenylindole (DAPI, Sigma). After thorough rinsing with PBST, cells were mounted using VECTASHIELD (Vector Laboratories, Inc.) and examined under a confocal microscope (Leica SP5, Leica) to determine the percentage of cells with LC3 punctate dots. Three to four independent experiments were performed, and at least 300 cells were analyzed in each experiment.

### Detection of apoptosis

Flow cytometry was used to detect cells containing cleaved PARP (a marker of apoptosis), as previously described [48].

### Detection of lysosome membrane permeability (LMP)

LysoTracker® Green (LTG, Invitrogen) was used to stain the acidic compartment of cells. After treatment with the test compounds for 7 h, cells were stained with 100 nM LTG for 30 min. Cells were subsequently collected and thoroughly rinsed with PBS. The intensity of cellular LTG fluorescence was measured using a flow cytometer at FL1 (FACSCanto II, BD Biosciences). Dead cells were excluded from the analysis by FSC/SSC analysis. A decrease in fluorescence intensity indicates LMP. The mean FL1 for each treatment group was calculated and normalized to that of untreated cells. Negative control cells were untreated and unstained with LTG.

A separate group of cells were incubated for 14 h with 1 mg/ml fluorescein isothiocyanate (FITC)-labeled dextran (10 kDa, Sigma), which can be taken up via endocytosis, and stored in functional lysosomes. Cells were washed thoroughly with culture media to remove excess fluorescence dextran, and then treated with vehicle or test compound for 3 h. The cells were then examined under a Zeiss Axiovert 200 inverted fluorescence microscope and photographed with a Zeiss camera (AxioCam ICm1).

### Transfection with MEK1 expression vectors

Transfections were performed using Lipofectamine 2000 reagent (Invitrogen). Cells were transfected with plasmids encoding wild type MEK1 (MEK1-WT, Clontech), constitutively active MEK1 (MEK1-EE), or a dominant-negative MEK1 (MEK1-AA) [49]. At 24 h after transfection, cells were treated with drugs for the indicated period of time, and then subjected to immunoblotting or viability analysis.

## SUPPLEMENTARY FIGURES






