# Supplementary material for: Derivatives of 6-cinnamamido-quinoline-4-carboxamide impair lysosome function and induce apoptosis
Source: Oncotarget. 2016 May 13;7(25):38078–90. doi: 10.18632/oncotarget.9348 (PMC5122373; doi:10.18632/oncotarget.9348)
Supplement: Supplementary file 1 [file oncotarget-07-38078-s001.pdf]

Derivatives of 6-cinnamamido-quinoline-4-carboxamide impair lysosome function and induce apoptosis

SUPPLEMENTARY DATA

Chemical synthesis

General chemistry

All commercial chemicals and solvents were reagent grade and were used without further purification, unless otherwise specified. All reactions were performed under a nitrogen atmosphere in dried glassware, unless otherwise noted. Melting points were determined in open capillaries on a Fargo melting point apparatus and were uncorrected. Thin-layer chromatography (TLC) was performed on silica gel G60 F254 (Merck, Merck KGaA, Darmstadt, Germany), and short wavelength ultraviolet (UV) light was used for visualization. The purity of all tested compounds was ≥95% based on analytical HPLC (high-performance liquid chromatography). HPLC was performed on an Elite instrument with a Mightysil RP-18 (250 mm × 4.6 mm) column. Compounds were detected by UV at 254 nm. The mobile phase was acetonitrile/water containing 0.5% ammonium acetate (50:50 v/v) with a flow rate of 1 mL/min. The injection volume was 10 μL. ^{1}H NMR spectra and ^{13}C NMR spectra were recorded on a Bruker AVANCE 600 DRX (Institute of Biomedical Sciences, Academia Sinica) and/or 400 MHz Bruker (Institute of Biological Chemistry, Academia Sinica) Top-Spin spectrometer in the indicated solvents. The proton chemical shifts were reported in parts per million (δ ppm) relative to $(CH_3)_4Si$ (TMS), and coupling constants (J) were reported in hertz (Hz). Abbreviations used are as follows: s, singlet; d, doublet; t, triplet; m, multiplet; brs, broad singlet.

Chemical synthesis of 6-cinnamamido-quinoline-4-carboxamide (CiQ) derivatives

6-Cinnamamido-quinoline-4-carboxamide (CiQ) derivatives were synthesized starting from the known 6-nitro-2-phenylquinoline-4-carboxylic acid intermediate **8**,[1] as shown in Scheme 1. Compound **8** was synthesized by reacting the commercially available 5-nitroisatin (**6**) and acetophenone (**7**) in a mixture of glacial acetic acid and concentrated HCl under heating at 75°C using a modified Pfitzinger approach.[2] Intermediate **8** was treated with thionyl chloride at reflux, and the reaction mixture was then evaporated to dryness under reduced pressure. The residue was reacted with *N,N*-dimethyl-1,2-ethanediamine (**9**) in chloroform to give carboxamide **10**. The nitro function of compound **10** was reduced to the corresponding 6-amino quinoline derivative **11** by reductive hydrogenation (10% Pd/C, H_2/cat. amount HCl). Reaction of compound **11** with the various cinnamic acid chloride compounds (**13a-r**; these in turn were freshly prepared from the corresponding known cinnamic acid compounds (**12a-r**) by treatment with thionyl chloride) resulted in the generation of the desired 6-cinammamido-quinoline derivatives (**5a-r**).

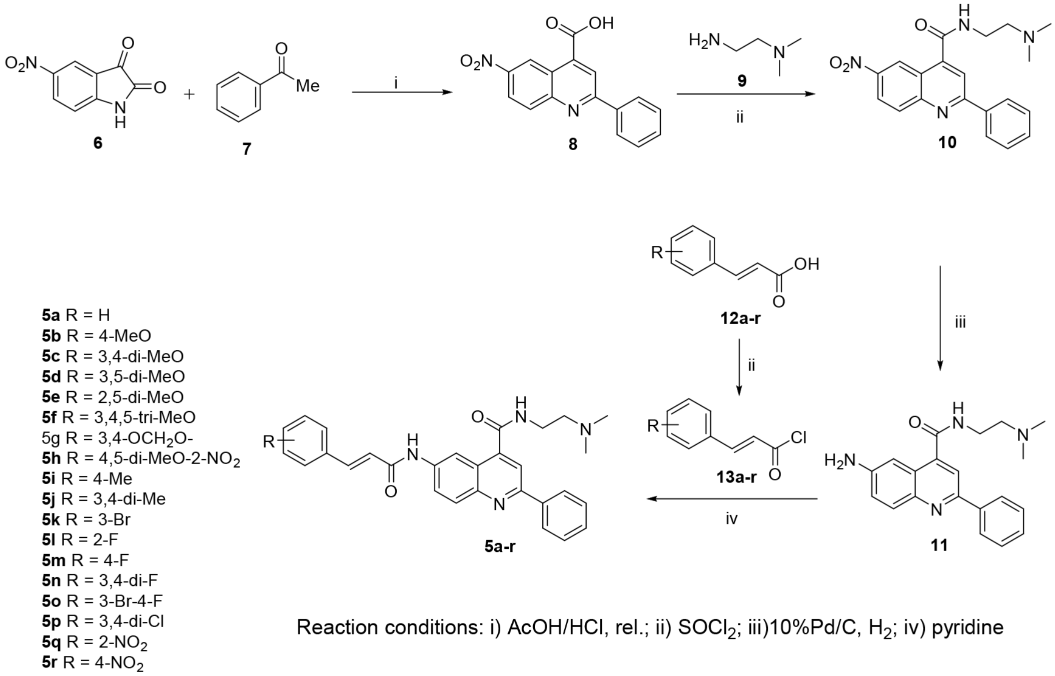

Scheme 1. Chemical synthesis of 6-cinnamamido-quinoline-4-carboxamide (CiQ) derivatives

6-Nitro-2-phenylquinoline-4-carboxylic acid (8)

Acetophenone **7** (3.6 g, 30 mmol) was added to a suspension of 5-nitroisatin **6** (5.73 g, 30 mmol) in glacial acetic acid (90 mL), in a 250 mL three-neck flask equipped with a reflux condenser. The reaction mass was heated at 75°C, followed by slow addition of conc. HCl (30 mL). The resulting mixture was stirred for 17 h at reflux temperature. The mixture was allowed to cool to room temperature and then diluted with water (300 mL). The solid precipitate was collected by filtration, and washed with water. The solid was then recrystallized from ethanol to give 6-nitro-2-phenylquinoline-4-carboxylic acid **8** (6 g, 69%) as a solid; mp 230-232°C (Reported 238 - 239°C)[1]; ^{1}H NMR (DMSO-d_6) δ 7.61–7.59 (m, 3H, ArH), 8.33–8.28 (m, 3H, ArH), 8.50 and 8.48 (dd, 1H, J = 2.60 and 9.2 Hz, ArH), 8.63 (s, 1H, ArH), 9.65 (d, 1H, J = 2.52 Hz).

***N*-(2-(Dimethylamino)ethyl)-6-nitro-2-phenylquinoline-4-carboxamide (10)**

6-nitro-2-phenylquinoline-4-carboxylic acid **8** (4.41 g, 15 mmol) was dissolved in thionyl chloride (40 mL) under a nitrogen atmosphere at 0°C. The reaction mixture was heated at 90°C for 2 h. The excess thionyl chloride was removed *in vacuo* to afford acid chloride intermediate as an orange solid, which was dissolved in dry chloroform (100 mL) under a nitrogen atmosphere. A solution of N,N-dimethyl-1,2-ethanediamine (**9**, 2.70 mL, 25 mmol) in chloroform (10 mL) was added dropwise at 0 °C to the above solution, and the reaction mixture was then stirred for 2 h at room temperature, before being quenched with sodium bicarbonate solution. The reaction mixture was diluted with chloroform and washed with saturated sodium bicarbonate solution, followed by brine solution. The organic layer was separated and dried over anhydrous Na_2SO_4, filtered, and concentrated. The product was purified by silica gel column chromatography using MeOH/$CHCl_3$ (5:95) to give *N*-(2-(dimethylamino)ethyl)-6-nitro-2-phenylquinoline-4-carboxamide **10** (4.8 g, 90%) as a light brown solid; mp 245–246°C; ^{1}H NMR (DMSO-d_6) δ 2.27 (s, 6H, 2×CH_3), 2.54 (d, 2H, J = 6.08 Hz, CH_2), 3.54 (t, 2H, J = 6.1 Hz, CH_2), 7.61–7.59 (m, 3H, 3×ArH), 8.27 (d, 1H, J = 9.2 Hz, CH_2), 8.34 (s, 2H, ArH), 8.48–8.47 (m, 1H, ArH), 9.22 (m, 1H, NH, exchangeable), 9.03 (m, 1H, ArH); ESI-HRMS for $C_{20}H_{20}N_4O_3$ $[M+H]^+$: calcd 365.1614; found 335.1602.

***N*-(2-(Dimethylamino)ethyl)-6-amino-2-phenylquinoline-4-carboxamide (11)**

A mixture of *N*-(2-(dimethylamino)ethyl)-6-nitro-2-phenylquinoline-4-carboxamide **10** (3.64 g, 10 mmol) in MeOH (400 mL) containing conc. HCl (0.5 mL) and a catalytic amount of 10% Pd/C (0.4 g) was hydrogenated at 35 psi for 6 h at room temperature. The mixture was then filtered through a pad of Celite, concentrated *in vacuo* to *N*-(2-(dimethylamino)ethyl)-6-amino-2-phenylquinoline-4-carboxamide **6** (2.8 g, 85%) as an orange solid; mp 217–218°C; ^{1}H NMR (DMSO-d_6) δ 1.79 (t, 2H, J = 5.56 Hz, CH_2), 2.18 (s, 6H, 2×CH_3), 2.38 (t, 2H, CH_2), 5.78 (s, 2H, NH_2, exchangeable), 7.53 (d, 3H, J = 5.72 Hz, 3×ArH), 8.23 (d, 4H, J = 9.52 Hz, 4×ArH), 8.29 (d, 1H, J = 3.44 Hz, ArH), 9.07 (t, 1H, ArH), 9.21 (d, 1H, J = 2.56 Hz, NH, exchangeable). ESI-HRMS for $C_{20}H_{22}N_4O$ $[M+H]^+$: calcd 335.1872; found 335.1892.

***N*-(2-(Dimethylamino)ethyl)-6-(3-phenylacrylamido)-2-phenylquinoline-4-carboxamide (5a)**

A solution of cinnamoyl chloride **13a** [freshly prepared by reacting cinnamic acid (**12a**, 0.59 g, 4 mmol) with $SOCl_2$ (10 mL) at 90°C] in dry chloroform (10 mL) was slowly added under a nitrogen atmosphere to a cooled (0°C) solution of 6-amino-*N*-(2-(dimethylamino)ethyl)-2-phenylquinoline-4-carboxamide (**11**, 1.1 g, 3.33 mmol) and pyridine (3 mL) in dry chloroform (30 mL). After being stirred at room temperature for 6 h under a nitrogen atmosphere, the solution was carefully washed with a saturated solution of $NaHCO_3$ and brine. The organic layer was separated, dried over anhydrous Na_2SO_4, and evaporated to dryness. The crude product obtained was purified by silica gel column chromatography using $CHCl_3$/MeOH (100:3) as an eluent system. The fractions containing the main product were combined and evaporated *in vacuo* to dryness. The residue was then crystallized from CH2Cl_2/EtOH to give **5a** as a solid. Yield 1.25 g (89%); mp 223–224°C; HPLC purity >99%; ^{1}H NMR (DMSO-d_6) δ 2.25 (s, 6H, 2×CH_3), 2.54 (t, J = 6.8 Hz, 2H, CH_2), 3.50 (q, J = 6.1 and 12.4 Hz, 2H, CH_2), 6.93 (d, J = 15.7 Hz, 1H, CH=CH), 7.42–7.49 (m, 2H, ArH), 7.51–7.56 (m, 2H, ArH), 7.57 (t, J = 7.3 Hz, 2H, ArH), 7.66–7.68 (m, 3H, CH=CH & ArH), 8.07 (s, 1H, ArH), 8.11 (d, J = 9.0 Hz, 1H, ArH), 8.21 (d, J = 8.8 Hz, 1H, ArH), 8.29 (d, J = 7.5 Hz,2H, ArH), 8.60 (s, 1H, ArH), 8.81 (t, J = 4.7 Hz, 1H, NH, exchangeable), 10.73 (s, 1H, NH, exchangeable). ^{13}C NMR (DMSO-d_6) δ 37.5, 40.4, 58.0, 112.9, 116.9, 122.0, 123.8, 124.0, 127.1, 127.8, 128.9, 129.1, 129.6, 129.9, 130.1, 134.7, 137.9, 138.4, 140.6, 142.4, 145.0, 154.2, 163.9, 166.8. ESI-HRMS for $C_{29}H_{28}N_4O_2$ $[M+H]^+$: calcd 465.2291; found 465.2292.

The following CiQ derivatives were synthesized by following the same procedure as that used for preparing compound **5a**.

***N*-(2-(Dimethylamino)ethyl)-6-(3-(4-methoxyphenyl)acrylamido)-2-phenyl-quinoline-4-carboxamide (5b)**

Compound **5b** was prepared from **11**(1.1 g, 3.3 mmol) and 4-methoxycinnamoyl chloride **13b** [freshly

prepared from 4-methoxycinnamic acid (**12b**, 0.72 g, 4 mmol)]. Yield 1.23 g (83%); mp 275–276°C; HPLC purity 98.2%. ^{1}H NMR (DMSO-d_6) δ 2.76 (s, 6H, 2×CH_3), 3.22 (m, 2H, CH_2), 3.71 (q, J = 5.5 and 11.3 Hz, 2H, CH_2), 3.82 (s, 3H, OCH_3), 6.84 (d, J = 15.7 Hz, 1H, CH=CH), 7.03 (d, J = 8.4 Hz, 1H, ArH), 7.50–7.62 (m, 4H, CH=CH & ArH), 8.10 (d, J = 9.1 Hz, 1H, ArH), 8.18–8.20 (m, 1H, ArH), 8.29 (s, 1H, ArH), 8.34 (d, J = 7.7 Hz, 2H, ArH), 8.68 (s, 1H, ArH), 9.12 (t, J = 5.3 Hz, 1H, NH, exchangeable), 10.73 (s, 1H, NH, exchangeable). ^{13}C NMR (DMSO-d_6) δ 35.2, 43.0, 55.3, 56.2, 112.5, 114.5, 117.3, 119.5, 123.8, 123.9, 127.1, 127.2, 128.8, 129.4, 129.6, 130.1, 138.2, 138.3, 140.3, 141.6, 144.9, 154.1, 160.7, 164.3, 167.2. ESI-HRMS for $C_{30}H_{30}N_4O_3$ [M+H]$^+$: calcd 495.2396; found 495.2395.

N-(2-(Dimethylamino)ethyl)-6-(3-(3,4-dimethoxylphenyl)acrylamido)-2-phenyl-quinoline-4-carboxamide (5c)

Compound **5c** was prepared from **11** (1.1 g, 3.3 mmol) and 3,4-dimethoxycinnamoyl chloride **13c** [freshly prepared from 3,4-dimethoxycinnamic acid (**12c**, 0.83 g, 4 mmol)]. Yield 1.38 g (88%); mp 262–263°C; HPLC purity >99%. ^{1}H NMR (DMSO-d_6) δ 2.64 (s, 6H, 2×CH_3), 3.06 (m, 2H, CH_2), 3.66–3.67 (m, 2H, CH_2), 3.82 (s, 3H, OCH_3), 3.84 (s, 3H, OCH_3), 6.87 (d, J = 15.6 Hz, 1H, CH=CH), 7.03 (d, J = 8.2 Hz, 1H, ArH), 7.03 (d, J = 8.3 Hz, 1H, ArH), 7.25 (s, 1H, ArH), 7.50–7.61 (m, 4H, CH=CH & ArH), 8.10 (d, J = 9.0 Hz, 1H, ArH), 8.19–8.21 (m, 1H, ArH), 8.25 (s, 1H, ArH), 8.33 (d, J = 7.9 Hz, 2H, ArH), 8.67 (s, 1H, ArH), 9.06 (m, 1H, NH, exchangeable), 10.72 (s, 1H, NH, exchangeable). ^{13}C NMR (DMSO-d_6) δ 36.7, 43.5, 55.4, 55.5, 56.6, 110.1, 111.8, 112.5, 117.2, 119.7, 121.9, 123.8, 123.9, 127.1, 127.4, 128.8, 129.6, 130.1, 138.1, 138.3, 140.7, 141.8, 144.9, 148.9, 150.5, 154.1, 164.3, 167.1. ESI-HRMS for $C_{31}H_{32}N_4O_4$ [M+H]$^+$: calcd 525.2502; found 525.2498.

N-(2-(Dimethylamino)ethyl)-6-(3-(3,5-dimethoxylphenyl)acrylamido)-2-phenyl-quinoline-4-carboxamide (5d)

Compound **5d** was prepared from **11** (0.5 g, 1.5 mmol) and 3,5-dimethoxycinnamoyl chloride **13d** [freshly prepared from 3,5-dimethoxycinnamic acid (**12d**, 0.42 g, 2 mmol)]. Yield 0.62 g (79%); mp 210–211°C; HPLC purity >99%. ^{1}H NMR (DMSO-d_6) δ 2.24 (s, 6H, 2×CH_3), 2.53 (t, J = 7.0 Hz, 2H, CH_2), 3.49 (q, J = 6.5 and 12.9 Hz, 2H, CH_2), 3.8 (s, 6H, 2×OCH_3), 7.57 (t, J = 2.0 Hz, 1H, ArH), 6.82–6.83 (m, 2H, ArH),6.89 (d, J = 15.7 Hz, 1H, CH=CH), 7.50–7.53 (m, 1H, ArH), 7.56–7.60 (m, 3H, ArH), 8.07 (s, 1H, ArH), 8.09–8.11 (m, 1H, ArH), 8.20 and 8.22 (dd, J = 2.2 and 9.2 Hz, 1H, ArH), 8.28 (d, J = 7.3 Hz, 2H, ArH), 8.57 (d, J = 2.1 Hz, 1H, ArH), 8.80 (t, J = 5.5 Hz, 1H, NH, exchangeable), 10.75 (s, 1H, NH, exchangeable). ^{13}C NMR (DMSO-d_6) 37.4, 45.3, 55.3, 58.0, 102.1, 105.6, 112.7, 116.9, 122.6, 123.7, 124.0, 127.0, 128.8, 129.6, 130.1, 136.6, 137.8, 138.3, 140.6, 142.4, 144.9, 154.2, 160.7, 163.8, 166.7. ESI-HRMS for $C_{31}H_{32}N_4O_4$ [M+H]$^+$: calcd 525.2502; found 525.2487.

N-(2-(Dimethylamino)ethyl)-6-(3-(2,5-dimethoxyphenyl)acrylamido)-2-phenyl-quinoline-4-carboxamide (5e)

Compound **5e** was prepared from **11** (1.1 g, 3.3 mmol) and 2,5-dimethoxycinnamoyl chloride **13e** [freshly prepared from 2,5-dimethoxycinnamic acid (**12e**, 0.83 g, 4 mmol)]. Yield 1.22 g (77%); mp 185–186°C; HPLC purity >99%. ^{1}H NMR (DMSO-d_6) δ 2.24 (s, 6H, 2×CH_3), 2.53 (t, J = 6.8 Hz, 2H, CH_2), 3.50 (q, J = 6.3 and 12.6 Hz, 2H, CH_2), 3.78 (s, 3H, CH_3), 3.86 (s, 3H, CH_3), 6.96 (d, J = 15.7 Hz, 1H, CH=CH), 6.99–7.07 (m, 2H, ArH), 7.17 (d, J = 2.3 Hz, 1H, ArH), 7.50–7.53 (m, 1H, ArH), 7.57 (t, J = 7.4 Hz, 2H, ArH), 7.86 (d, J = 15.7 Hz, 1H, CH=CH), 8.07 (s, 1H, ArH), 8.11 (d, J = 9.0 Hz, 1H, ArH), 8.22 (d, J = 9.0 Hz, 1H, ArH), 8.29 (d, J = 7.7 Hz, 2H, ArH), 8.57 (s, 1H, ArH), 8.79 (t, J = 5.4 Hz, 1H, NH, exchangeable), 10.55 (s, 1H, NH, exchangeable). ^{13}C NMR (DMSO-d_6) δ 37.4, 45.3, 55.4, 56.1, 58.1, 112.6, 112.7, 113.1, 116.8, 116.9, 122.7, 123.6, 123.7, 124.0, 127.0, 128.8, 129.6, 130.1, 135.6 138.0, 138.3, 142.4, 144.9, 152.1, 153.1, 154.1, 164.2, 166.7. ESI-HRMS for $C_{31}H_{32}N_4O_4$ [M+H]$^+$: calcd 525.2502; found 525.2490.

N-(2-(Dimethylamino)ethyl)-6-(3-(3,4,5-trimethoxylphenyl)acrylamido)-2-phenyl-quinoline-4-carboxamide (5f)

Compound **5f** was prepared from **11** (1.1 g, 3.3 mmol) and 3,4,5-trimethoxycinnamoyl chloride **13f** [freshly prepared from 3,4,5-trimethoxycinnamic acid (**12f**, 0.95 g, 4 mmol)]. Yield 1.32 g (80%); mp 209–210°C; HPLC purity >99%. ^{1}H NMR (DMSO-d_6) δ 2.23 (s, 6H, 2×CH_3), 2.55 (t, J = 6.7 Hz, 2H, CH_2), 3.45 (q, J = 6.6 and 12.7 Hz, 2H, CH_2), 3.71 (s, 3H, OCH_3), 3.85 (s, 6H, 2×OCH_3), 7.04 (d, J = 15.8 Hz, 1H, CH=CH), 7.49–7.53 (m, 1H, ArH), 7.57 (t, J = 7.2 Hz, 2H, ArH), 7.62 (d, J = 15.8 Hz, 1H, CH=CH), 7.65–7.66 (m, 1H, ArH), 7.69–7.72 (m, 1H, ArH), 8.07 (s, 1H, ArH), 8.10–8.11 (m, 1H, ArH), 8.26 (s, 1H, ArH), 8.38–8.39 (m, 2H, ArH), 8.52 (s, 1H, ArH), 8.93 (t, J = 5.4 Hz, 1H, NH, exchangeable), 10.79 (s, 1H, NH, exchangeable). ^{13}C NMR (DMSO-d_6) 34.6, 42.3, 55.9, 56.1, 60.2, 110.2, 112.4, 117.5, 118.3, 121.5, 124.2, 124.6, 127.8, 128.5, 128.9, 130.3, 130.4, 136.6, 138.6, 139.1, 140.8, 142.9, 153.1, 153.7, 157.9, 158.2, 164.2, 166.7. ESI-HRMS for $C_{32}H_{34}N_4O_5$ [M+H]$^+$: calcd 555.2607; found 555.2624.

_N_-(2-(Dimethylamino)ethyl)-2-phenyl-6-(3-(benzo[d][1,3]dioxol-5-yl)acrylamido)quinoline-4-carboxamide (5g)

Compound **5g** was prepared from **11**(1.1 g, 3.3 mmol) and 3,4-(methylenedioxy)cinnamoyl chloride **13g** [freshly prepared from 3,4-(methylenedioxy)cinnamic acid (**12g**, 0.77 g, 4 mmol)]. Yield 1.3 g (78%); mp 255–256°C; HPLC purity >99%. ^{1}H NMR (DMSO-d_6) δ 2.24 (s, 6H, 2×CH_3), 2.55 (t, J = 6.7 Hz, 2H, CH_2), 3.50 (q, J = 6.0 and 12.1 Hz, 2H, CH_2), 6.10 (s, 2H, -OCH_2O-), 6.76 (d, J = 15.6 Hz, 1H, CH=CH), 7.00 (d, J = 7.9 Hz, 1H, ArH), 7.18 (d, J = 7.9 Hz, 1H, ArH), 7.23 (s, 1H, ArH), 7.50–7.60 (m, 4H, CH=CH, ArH), 8.07 (s, 1H, ArH), 8.11 (d, J = 9.0 Hz, 1H, ArH), 8.23 (d, J = 9.0 Hz, 1H, ArH), 8.29 (d, J = 7.6 Hz, 2H, ArH), 8.57 (s, 1H, ArH), 8.79 (t, J = 5.4 Hz, 1H, NH, exchangeable), 10.55 (s, 1H, NH, exchangeable). ^{13}C NMR (DMSO-d_6) δ 37.4, 45.3, 58.1, 101.5, 106.3, 108.7, 112.7, 116.9, 120.0, 123.7, 123.8, 124.0, 127.0, 128.8, 129.0, 129.5, 130.1, 138.0, 138.3, 140.5, 142.4, 144.9, 148.0, 148.8, 154.1, 164.1, 166.8. ESI-HRMS for $C_{30}H_{28}N_4O_4$ $[M+H]^+$: calcd 509.2189; found 509.2184.

_N_-(2-(Dimethylamino)ethyl)-6-(3-(4,5-dimethoxy-2-nitrophenyl)acrylamido)-2-phenylquinoline-4-carboxamide (5h)

Compound **5h** was prepared from **11** (1.1 g, 3.3 mmol) and 4,5-dimethoxy-2-nitrocinnamoyl chloride **13h** [freshly prepared from 4,5-dimethoxy-2-nitrocinnamic acid (**12h**, 1.01 g, 4 mmol)]. Yield 1.50 g (88%); mp 230–231°C; HPLC purity 97.6%. ^{1}H NMR (DMSO-d_6) δ 2.52 (s, 6H), 2.92 (m, 2H), 3.61–3.63 (m, 2H), 3.91 (s, 3H), 3.99 (s, 3H), 6.95 (d, J = 15.41 Hz, 1H), 7.31 (s, 1H), 7.50–7.53 (m, 1H), 7.56–7.59 (m, 2H), 7.68 (s, 1H), 8.01 (d, J = 15.54, 1H), 8.10–8.19 (m, 3H), 8.30 (d, J = 7.6 Hz, 2H), 8.72 (s, 1H), 8.96 (t, J = 5.6 Hz, 1H, exchangeable), 10.84 (s, 1H, exchangeable). ^{13}C NMR (DMSO-d_6) δ 35.9, 39.0, 43.8, 56.2, 56.2, 56.9, 107.9, 109.9, 112.8, 117.1, 123.7, 123.9, 124.3, 125.6, 127.0, 128.8, 129.6, 130.1, 135.9, 137.7, 138.3, 141.1, 141.9, 145.0, 149.4, 152.8, 154.2, 163.4, 166.9. ESI-HRMS for $C_{31}H_{31}N_5O_6$ $[M+H]^+$: calcd 570.2353; found 570.2344.

_N_-(2-(Dimethylamino)ethyl)-6-(3-(4-methylphenyl)acrylamido)-2-phenyl-quinoline-4-carboxamide (5i)

Compound **5i** was prepared from **11** (1.1 g, 3.3 mmol) and 4-methylcinnamoyl chloride **13i** [freshly prepared from 4-methylcinnamic acid (**12i**, 0.65 g, 4 mmol)]. Yield 1.28 g (82%); mp 246–247°C; HPLC purity 97.9%. ^{1}H NMR (DMSO-d_6) δ 2.33 (s, 6H, 2×CH_3), 2.35 (s, 3H, CH_3), 2.65 (t, J = 6.8 Hz, 2H, CH_2), 3.53 (q, J = 6.6 and 12.6 Hz, 2H, CH_2), 6.88 (d, J = 15.7 Hz, 1H, CH=CH), 7.28 (d, J = 8.0 Hz, 2H, ArH), 7.50–7.59 (m, 5H, CH=CH & ArH), 7.62 (d, 1H, ArH), 8.09–8.11 (m, 2H, ArH), 8.20 and 8.22 (dd, J = 2.3 and 9.2 Hz, 1H, ArH), 8.30 (d, J = 7.2 Hz, 2H, ArH), 8.60 (d, J = 2.3 Hz, 1H, ArH), 8.84 (t, J = 5.6 Hz, 1H, NH, exchangeable), 10.64 (s, 1H, NH, exchangeable). ^{13}C NMR (DMSO-d_6) 20.9, 37.1, 44.9, 57.7, 112.7, 116.9, 121.0, 123.8, 123.9, 127.0, 127.8, 128.9, 129.6, 129.7, 130.1, 131.9, 137.9, 138.3, 139.7, 140.6, 142.3, 144.9, 154.1, 164.0, 166.8. ESI-HRMS for $C_{30}H_{30}N_4O_2$ $[M+H]^+$: calcd 479.2447; found 479.2439.

_N_-(2-(Dimethylamino)ethyl)-6-(3-(3,4-dimethylphenyl)acrylamido)-2-phenyl-quinoline-4-carboxamide (5j)

Compound **5j** was prepared from **11** (1.1 g, 3.3 mmol) and 3,4-dimethylcinnamoyl chloride **13j** [freshly prepared from 3,4-dimethylcinnamic acid (**12j**, 0.71 g, 4 mmol)]. Yield 1.35 g (92%); mp 263–264°C; HPLC purity 98.3%. ^{1}H NMR (DMSO-d_6) δ 2.23 (s, 6H), 2.26 (s, 3H), 2.27 (s, 3H), 2.54 (t, J = 7.0 Hz, 2H), 3.49 (q, J = 6.6 and 13.0 Hz, 2H), 6.86 (d, J = 15.7 Hz, 1H), 7.21–7.23 (m, 1H), 7.39–7.37 (m, 1H), 7.43 (s, 1H), 7.53–7.50 (m, 1H), 7.56–7.60 (m, 3H), 8.10–8.11 (m, 2H), 8.20 (dd, J = 2.3 and 9.1 Hz, 1H), 8.29 (m, 2H), 8.58–8.59 (m, 1H), 8.79 (t, J = 5.6 Hz, 1H, exchangeable), 10.61 (s, 1H, exchangeable). ^{13}C NMR (DMSO-d_6) δ 19.4, 37.4, 45.3, 58.0, 112.7, 116.9, 120.8, 123.8, 124.0, 125.4, 127.0, 128.8, 128.9, 129.6, 130.0, 130.1, 132.3, 136.9, 138.0, 138.3, 138.6, 140.7, 142.2, 145.0, 154.1, 164.1, 166.8. ESI-HRMS for $C_{31}H_{32}N_4O_2$ $[M+H]^+$: calcd 493.2604; found 493.2602.

_N_-(2-(Dimethylamino)ethyl)-6-(3-(3-bromophenyl)acrylamido)-2-phenyl-quinoline-4-carboxamide (5k)

Compound **5k** was prepared from **11** (1.1 g, 3.3 mmol) and 3-bromocinnamoyl chloride **13k** [freshly prepared from 3-bromocinnamic acid (**12k**, 0.91 g, 4 mmol)]. Yield 1.25 g (77%); mp 226–227°C; HPLC purity >99%. ^{1}H NMR (DMSO-d_6) δ 2.25 (s, 6H), 2.54 (t, J = 7.0 Hz, 2H), 3.49 (q, J = 6.6 and 12.9 Hz, 2H), 6.96 (d, J = 15.8 Hz, 1H), 7.43 (t, 7.8 Hz, 1H), 7.50 – 7.53 (m, 1H), 7.56 – 7.59 (m, 2H), 7.62 – 7.68 (m, 3H), 7.87 (s, 1H), 8.12 – 8.08 (m, 2H), 8.22 (dd, J = 2.2 and 9.1 Hz, 1H), 8.27 – 8.29 (m, 2H), 8.58 – 8.59 (m, 1H), 8.80 (t, J = 5.6 Hz, 1H, exchangeable), 10.66 (s, 1H, exchangeable). ^{13}C NMR (DMSO-d_6) δ 37.4, 45.3, 58.0, 112.9, 117.0, 122.3, 123.7, 123.8, 124.0, 126.6, 127.0, 128.9, 129.6, 130.2, 130.3, 131.1, 132.4, 137.2, 137.8, 138.3, 138.8, 142.4, 145.0, 154.2, 163.4, 166.7. ESI-HRMS for $C_{29}H_{27}BrN_4O_2$ $[M+H]^+$: calcd 543.1396; found 543.1388.

N-(2-(Dimethylamino)ethyl)-6-(3-(2-fluorolphenyl) acrylamido)-2-phenylquinoline-4-carboxamide (5l)

Compound **5l** was prepared from **11** (0.5 g, 1.5 mmol) and 2-fluorocinnamoyl chloride **13l** [freshly prepared from 2-fluorocinnamic acid (**12l**, 0.33 g, 2 mmol)]. Yield 0.53 g (74%); mp 212–213°C; HPLC purity >99%. ^{1}H NMR (DMSO-d_6) δ 2.25 (s, 6H, 2×CH_3), 2.55 (t, J = 7.0 Hz, 2H, CH2), 3.49 (q, J = 6.6 and 12.9 Hz, 2H, CH2), 7.03 (d, J = 15.8 Hz, 1H, CH=CH), 7.31–7.36 (m, 2H, ArH), 7.47–7.53 (m, 2H, ArH), 7.57 (t, 2H, ArH), 7.71 (d, J = 15.8 Hz, 1H, CH=CH), 7.74 (t, J = 7.6 Hz, 1H, ArH), 8.08 (s, 1H, ArH), 8.10–8.12 (m, 1H, ArH), 8.21 and 8.22 (dd, J = 2.1 and 9.4 Hz, 1H, ArH), 8.28 (d, J = 7.4 Hz, 2H, ArH), 8.60 (d, J = 2.0 Hz, 1H, ArH), 8.80 (t, J = 5.5 Hz, 1H, NH, exchangeable), 10.75 (s, 1H, NH, exchangeable). ^{13}C NMR (DMSO-d_6) 37.4, 45.3, 58.0, 113.0, 116.1, 116.3, 116.9, 122.3, 122.4, 123.7, 123.9, 124.9, 125.1, 127.0, 128.8, 129.6, 130.1, 131.7, 133.1, 137.8, 138.3, 142.4, 145.0, 154.2, 159.6, 161.6, 163.6, 166.7. ESI-HRMS for $C_{29}H_{27}FN_4O_2$ $[M+H]^+$: calcd 483.2196; found 483.2191.

N-(2-(Dimethylamino)ethyl)-6-(3-(4-fluorophenyl) acrylamido)-2-phenyl-quinoline-4-carboxamide (5m)

Compound **5m** was prepared from **11** (1.1 g, 3.3 mmol) and 4-fluorocinnamoyl chloride **13m** [freshly prepared from 4-fluorocinnamic acid (**12m**, 0.66 g, 4 mmol)]. Yield 0.98 g (68%); mp 257–258°C; HPLC purity >99%. ^{1}H NMR (DMSO-d_6) δ 2.24 (s, 6H, 2×CH_3), 2.54 (t, J = 6.9 Hz, 2H, CH_2), 3.49 (q, J = 6.4 and 12.8 Hz, 2H, CH_2),6.88 (d, J = 15.7 Hz, 1H, CH=CH), 7.31 (t, J = 8.6 Hz, 2H, ArH), 7.50–7.52 (m, 1H, ArH), 7.58–7.56 (m, 2H, ArH), 7.64 (d, J =15.7 Hz, 1H, CH=CH), 7.73–7.71 (m, 2H, ArH), 8.07 (s, 1H, ArH), 8.10 (d, J = 9.1 Hz, 1H, ArH), 8.22–8.24 (m, 1H, ArH), 8.34 (d, J = 7.7 Hz, 2H, ArH), 8.58 (s, 1H, ArH), 8.76 (t, J = 5.5 Hz, 1H, NH, exchangeable), 10.66 (s, 1H, NH, exchangeable). ^{13}C NMR (DMSO-d_6) δ 37.4, 45.2, 58.0, 112.8, 115.9, 116.0, 116.8, 121.9, 123.7, 123.9, 126.9, 128.8, 129.5, 129.9, 130.0, 130.1, 131.2, 131.3, 137.8, 138.3, 139.3, 142.4, 144.9, 154.1, 162.1, 163.7, 163.8, 166.7. ESI-HRMS for $C_{29}H_{27}FN_4O_2$ $[M+H]^+$: calcd 483.2196; found 483.2209.

N-(2-(Dimethylamino)ethyl)-6-(3-(3,4-difluorolphenyl) acrylamido)-2-phenylquinoline-4-carboxamide (5n)

Compound **5n** was prepared from **11** (1.1 g, 3.3 mmol) and 3,4-difluorocinnamoyl chloride **13n** [freshly prepared from 3,4-difluorocinnamic acid (**12n**, 0.74 g, 4 mmol)]. Yield 1.22 g (81%); mp 238–239°C; HPLC purity 97.3%. ^{1}H NMR (DMSO-d_6) δ 2.34 (s, 6H), 2.66 (t, J = 6.7 Hz, 2H), 3.54 (q, J = 12.7 Hz, 2H), 6.91 (d, J = 15.8 Hz, 1H), 7.59–7.50 (m, 5H), 7.66–7.63 (m, 1H), 7.77–7.73 (m, 1H), 8.12–8.10 (m, 2H), 8.23–8.21 (m, 1H), 8.29 (d, J = 7.3 Hz, 2H), 8.60 (d, J = 1.8 Hz, 1H), 8.86 (t, J = 5.5 Hz, 1H, NH, exchangeable), 10.73 (s, 1H, NH, exchangeable). ^{13}C NMR (DMSO-d_6) δ 37.0, 44.9, 57.7, 112.9, 116.5, 116.6, 117.0, 118.1, 118.2, 123.5, 123.8, 124.0, 127.1, 128.9, 129.6, 130.1, 137.8, 138.3, 138.3, 142.3, 149.0, 151.0, 145.0, 154.2, 163.5, 166.8. ESI-HRMS for $C_{29}H_{26}F_2N_4O_2$ $[M+H]^+$: calcd 501.2102; found 501.2110.

N-(2-(Dimethylamino)ethyl)-6-(3-(3-bromo-4-fluorophenyl)acrylamido)-2-phenylquinoline-4-carboxamide (5o)

Compound **5o** was prepared from **11** (0.5 g, 1.5 mmol) and 3-bromo-4-fluorocinnamoyl chloride **13o** [freshly prepared from 3-bromo-4-fluorocinnamic acid (**12o**, 0.49 g, 2 mmol)]. Yield 0.70 g (83%); mp 246–247°C; HPLC purity >99%. ^{1}H NMR (DMSO-d_6) δ 2.24 (s, 6H, 2×CH_3), 2.53 (t, J = 7.0 Hz, 2H, CH_2), 3.48 (q, J = 6.6 and 12.9 Hz, 2H, H_2), 6.89 (d, J = 15.7 Hz, 1H, CH=CH), 7.47–7.53 (m, 2H, ArH), 7.57 (t, J = 7.4 Hz, 2H, ArH), 7.63 (d, J = 15.7 Hz, 1H, CH=CH), 7.72–7.74 (m, 1H, ArH), 8.02–8.03 (m, 1H, ArH), 8.07 (s, 1H, ArH), 8.10 (d, J = 7.6 Hz, 1H, ArH), 8.20–8.22 (m, 1H, ArH),8.28 (d, J = 7.6 Hz, 2H, ArH), 8.56 (d, J = 1.5 Hz, 1H, ArH), 8.79 (t, J = 5.6 Hz, 1H, NH, exchangeable), 10.75 (s, 1H, NH, exchangeable). ^{13}C NMR (DMSO-d_6) 37.4, 45.3, 58.0, 108.7, 108.8, 112.9, 116.9, 117.3, 117.5, 123.5, 123.7, 123.9, 127.1, 128.8, 128.9, 129.6, 130.1, 132.8, 133.1, 137.7, 137.9, 138.3, 142.4, 144.9, 154.2, 157.8, 163.4, 166.7; ESI-HRMS for $C_{29}H_{26}BrFN_4O_2$ $[M+H]^+$: calcd 561.1301; found 561.1296.

N-(2-(Dimethylamino)ethyl)-6-(3-(3,4-dichlorophenyl) acrylamido)-2-phenyl-quinoline-4-carboxamide (5p)

Compound **5p** was prepared from **11** (0.5 g, 1.5 mmol) and 3,4-chlorocinnamoyl chloride **13p** [freshly prepared from 3,4-dichlorocinnamic acid (**12p**, 0.43 g, 2 mmol)]. Yield 0.63 g (79%); mp 259–260°C; HPLC purity 97.4%. ^{1}H NMR (DMSO-d_6) δ 2.24 (s, 6H, 2×CH_3), 2.53 (t, J = 6.9 Hz, 2H, CH_2), 3.49 (q, J = 6.5 and 12.8 Hz, 2H, CH_2), 6.96 (d, J = 15.6 Hz, 1H, CH=CH), 7.50–7.53 (m, 1H, ArH), 7.57 (t, J = 7.2 Hz, 2H, ArH), 7.63 (d, J = 15.6 Hz, 1H, CH=CH), 7.66–7.67 (m, 1H, ArH), 7.72–7.74 (m, 1H, ArH), 7.94 (d, J = 1.6 Hz, 1H, ArH), 8.07 (s, 1H, ArH), 8.10–8.11 (m, 1H, ArH), 8.20–8.23 (m, 1H, ArH),8.28 (d, J = 7.3 Hz, 2H, ArH), 8.57 (d, J = 1.9 Hz, 1H, ArH), 8.79 (t, J = 5.6 Hz, 1H, NH, exchangeable), 10.79 (s, 1H, NH, exchangeable). ^{13}C NMR (DMSO-d_6) 37.4, 45.2, 58.0, 112.9, 116.9, 123.7, 123.9, 124.4, 127.0, 127.4, 128.8, 129.6, 129.7, 130.1, 131.2, 131.7, 132.0, 135.6, 137.7, 137.8, 138.3, 142.4, 145.0, 154.2, 163.3, 166.7; ESI-HRMS for $C_{29}H_{26}Cl_2N_4O_2$ $[M+H]^+$: calcd 533.1511; found 533.1499.

_N_-(2-(Dimethylamino)ethyl)-6-(3-(2-nitrophenyl) acrylamido)-2-phenylquinoline-4-carboxamide (5q)

Compound **5q** was prepared from **11** (0.5 g, 1.5 mmol) and 2-nitrocinnamoyl chloride **13q** [freshly prepared from 2-nitrocinnamic acid (**12q**, 0.39 g, 2 mmol)]. Yield 0.56 g (74%); mp 222–223°C; HPLC purity >99%. ^{1}H NMR (DMSO-d_6) δ 2.24 (s, 6H, 2×CH_3), 2.54 (t, J = 7.0 Hz, 2H, CH_2), 3.50 (q, J = 6.5 and 12.9 Hz, 2H, CH_2), 7.10 (d, J = 15.7 Hz, 1H, CH=CH), 7.50–7.53 (m, 1H, ArH), 7.56–7.59 (m, 2H, ArH), 7.74–7.80 (m, 2H, ArH), 8.08 (s, 1H, ArH), 8.09–8.12 (m, 2H, ArH), 8.22–8.29 (m, 4H, ArH), 8.50 (s, 1H, ArH), 8.58 (dd, J = 2.0 Hz, 1H, ArH), 8.80 (t, J = 5.6 Hz, 1H, NH, exchangeable), 10.73 (s, 1H, NH, exchangeable). ^{13}C NMR (DMSO-d_6) 37.5, 45.3, 58.0, 112.9, 116.9, 121.7, 123.7, 123.9, 124.0, 125.0, 127.1, 128.8, 129.6, 130.2, 130.6, 134.1, 136.5, 137.7, 138.1, 138.3, 142.4, 145.0, 148.3, 154.2, 163.2, 166.7. ESI-HRMS for $C_{29}H_{27}N_5O_4$ $[M+H]^+$: calcd 510.2141; found 510.2146.

_N_-(2-(Dimethylamino)ethyl)-6-(3-(4-nitrophenyl) acrylamido)-2-phenylquinoline-4-carboxamide (5r)

Compound **5r** was prepared from **11** (1.1 g, 3.3 mmol) and 4-nitrocinnamoyl chloride **13r** [freshly prepared from 4-nitrocinnamic acid (**12r**, 0.77 g, 4 mmol)]. Yield 1.52 g (84%); mp 262–263°C; HPLC purity >99%. ^{1}H NMR (DMSO-d_6) δ 2.27 (s, 6H, 2×CH_3), 2.56 (t, J = 7.1 Hz, 2H, CH_2), 3.52 (q, J = 6.5 and 12.7 Hz, 2H, CH_2), 7.12 (d, J = 15.7 Hz, 1H, CH=CH), 7.53–7.62 (m, 3H, ArH), 7.75 (d, J = 15.7 Hz, 1H, CH=CH), 7.91 (d, J = 8.8 Hz, 1H, ArH), 8.21–8.28 (m, 3H, ArH), 8.31 (d, J = 8.8 Hz, 2H, ArH), 8.38 (d, J = 6.9 Hz, 2H, ArH), 8.49 (s, 1H, ArH), 8.78 (s, 1H, ArH), 8.80 (t, J = 5.7 Hz, 1H, NH, exchangeable), 10.75 (s, 1H, NH, exchangeable). ^{13}C NMR (DMSO-d_6) 34.6, 42.34, 55.61, 112.8, 118.2, 121.8, 124.1, 124.2, 124.4, 126.4, 127.6, 128.8, 128.9, 130.2, 134.3, 136.9, 137.7, 138.1, 138.2, 141.2, 142.7, 143.5, 147.7, 153.9, 163.4, 166.8. ESI-HRMS for $C_{29}H_{27}N_5O_4$ $[M+H]^+$: calcd 510.2141; found 510.2134.

REFERENCES

1. Wang LM, Hua L, Chen HJ, Sui YY, Shen W. One-pot synthesis of quinoline-4-carboxylic acid derivatives in water: Ytterbium perfluorooctanoate catalyzed Doebner reaction. J. Fluorine Chem. 2009; 130:406–409.
2. Lackey K, Sternbach DD. Synthesis of Substituted Quinoline-4-carboxylic Acids. Synthesis 1993; 1993:993-997.
