# Supplementary material for: Derivatives of 6-cinnamamido-quinoline-4-carboxamide impair lysosome function and induce apoptosis
Source: Oncotarget. 2016 May 13;7(25):38078–90. doi: 10.18632/oncotarget.9348 (PMC5122373; doi:10.18632/oncotarget.9348)

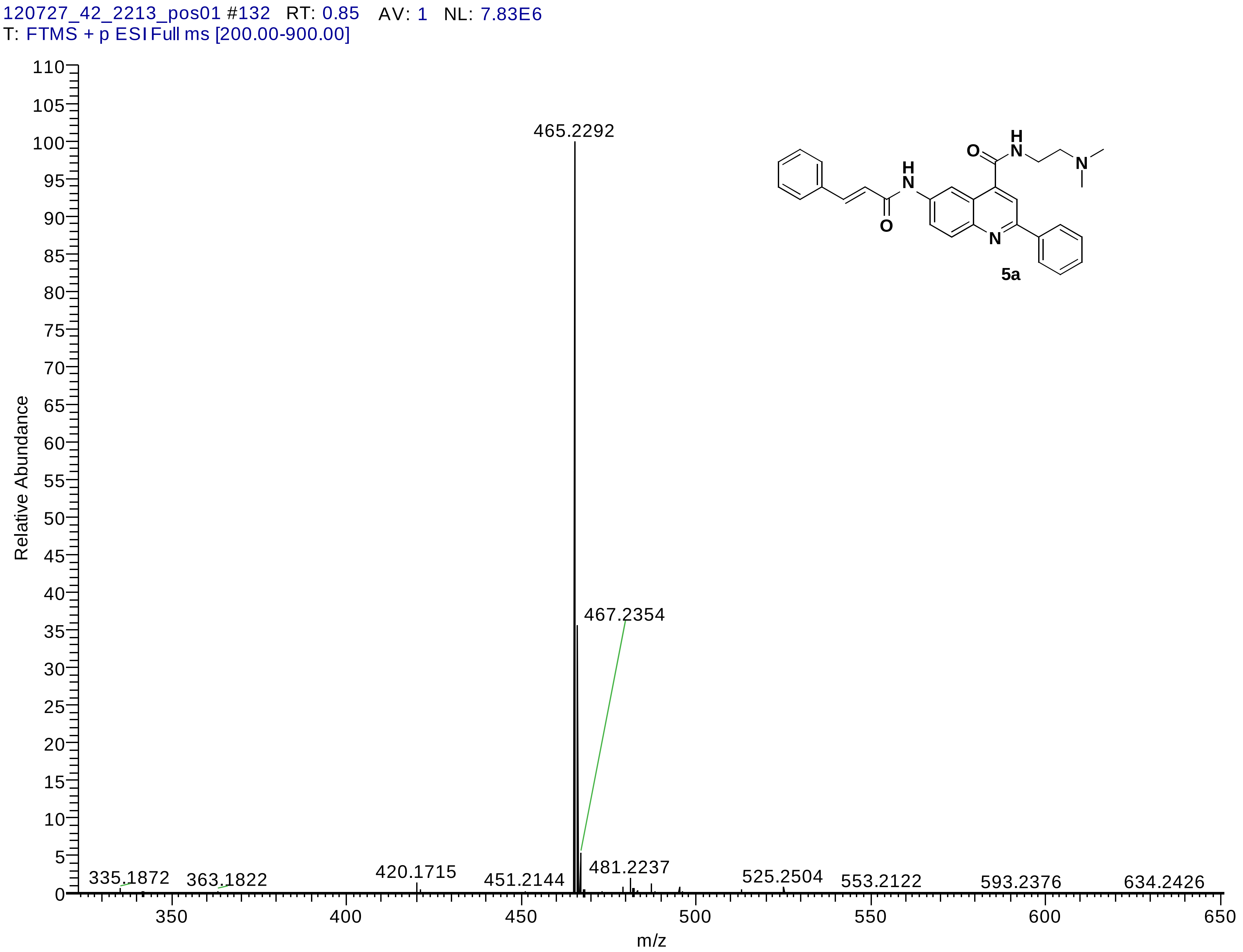
120727_42_2213_pos01 #132 RT: 0.85 AV: 1 NL: 7.83E6
T: FTMS + p ESI Full ms [200.00-900.00]
Relative Abundance
m/z
465.2292
467.2354
335.1872
363.1822
420.1715
451.2144
481.2237
525.2504
553.2122
593.2376
634.2426
5a

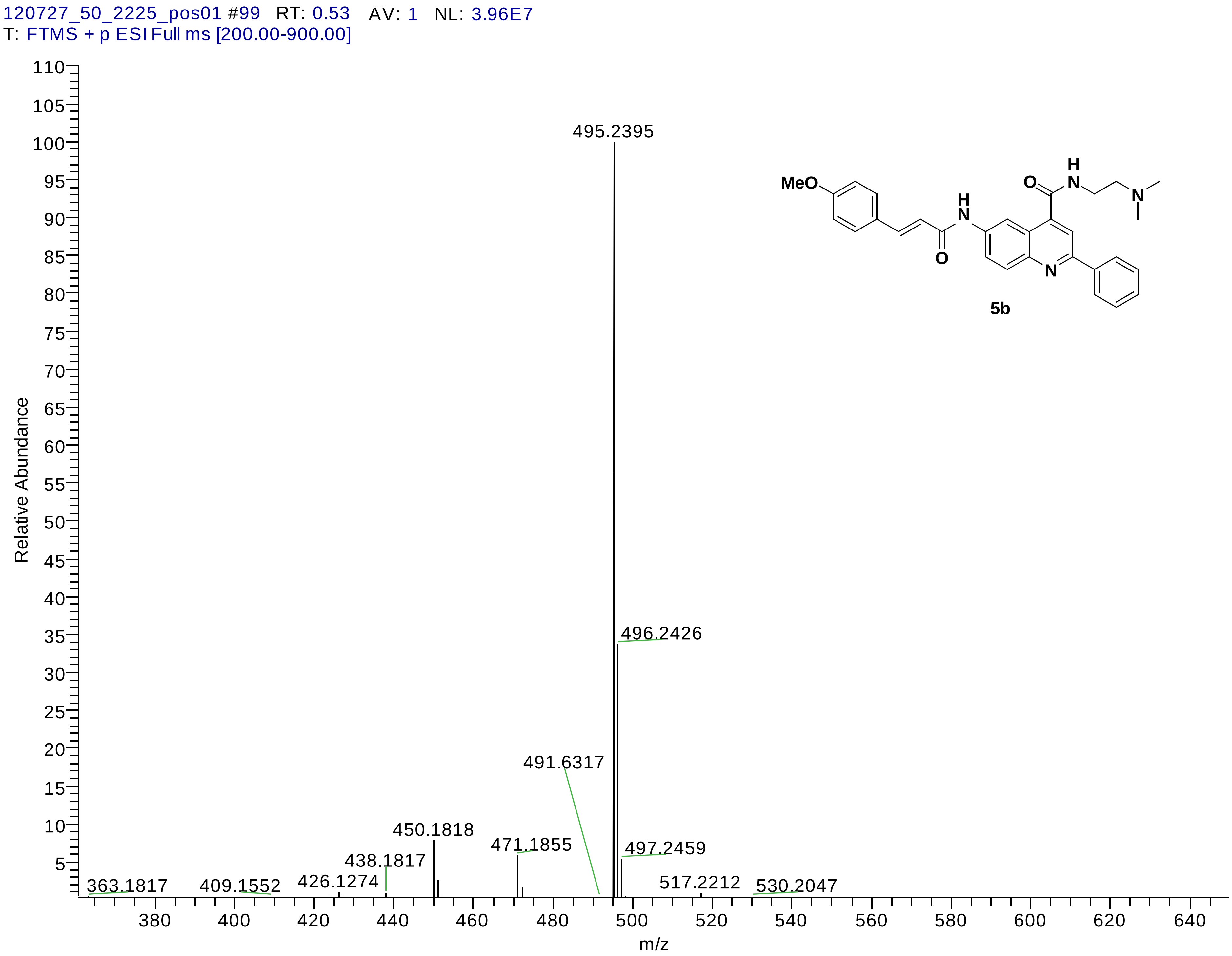
120727_50_2225_pos01 #99 RT: 0.53 AV: 1 NL: 3.96E7
T: FTMS + p ESI Full ms [200.00-900.00]
495.2395
496.2426
491.6317
450.1818
471.1855
497.2459
438.1817
426.1274
363.1817
409.1552
517.2212
530.2047
Relative Abundance
m/z
MeO
5b

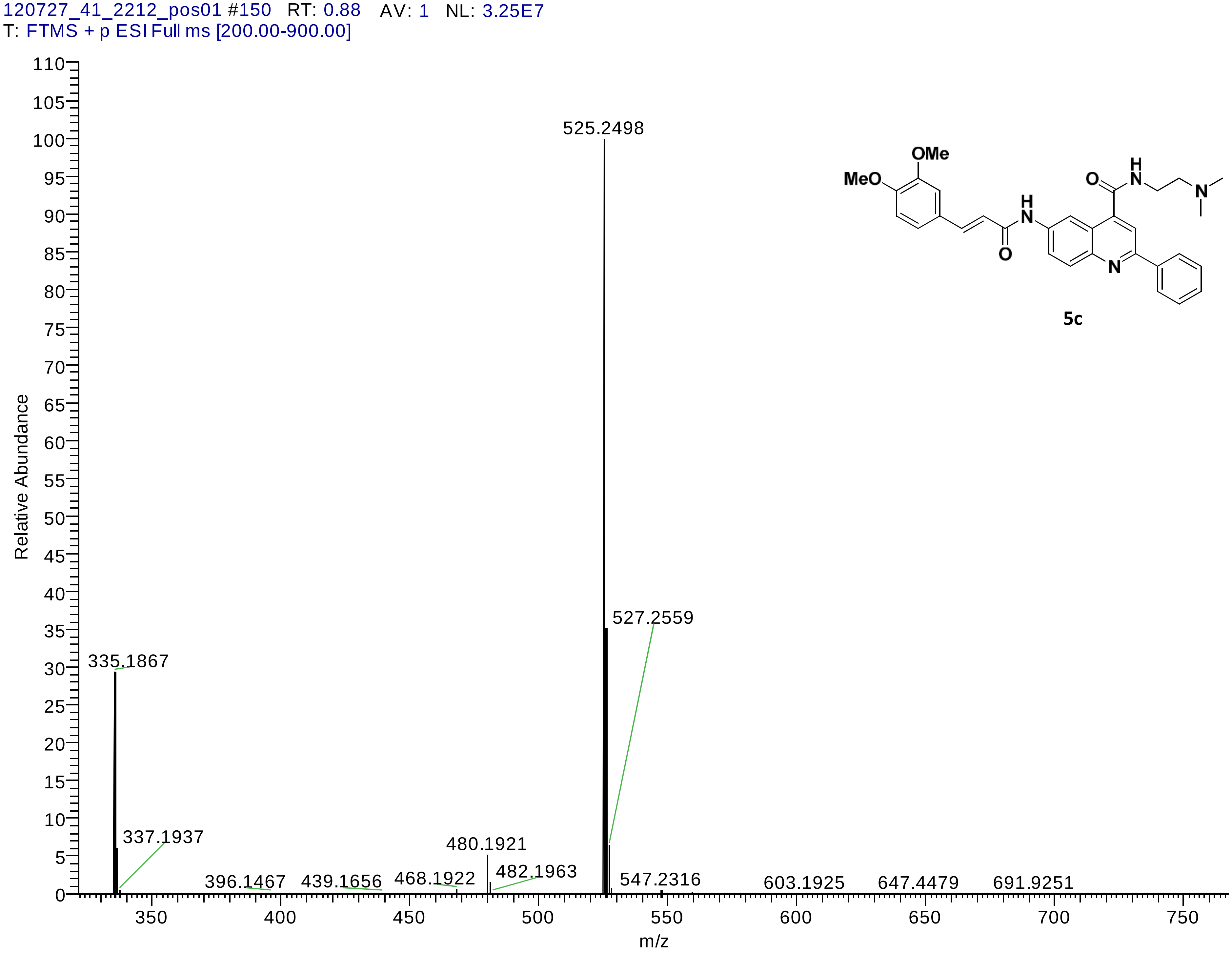
120727_41_2212_pos01 #150 RT: 0.88 AV: 1 NL: 3.25E7
T: FTMS + p ESI Full ms [200.00-900.00]
Relative Abundance
m/z
525.2498
527.2559
335.1867
337.1937
396.1467
439.1656
468.1922
480.1921
482.1963
547.2316
603.1925
647.4479
691.9251
OMe
MeO
H
N
O
N

5c

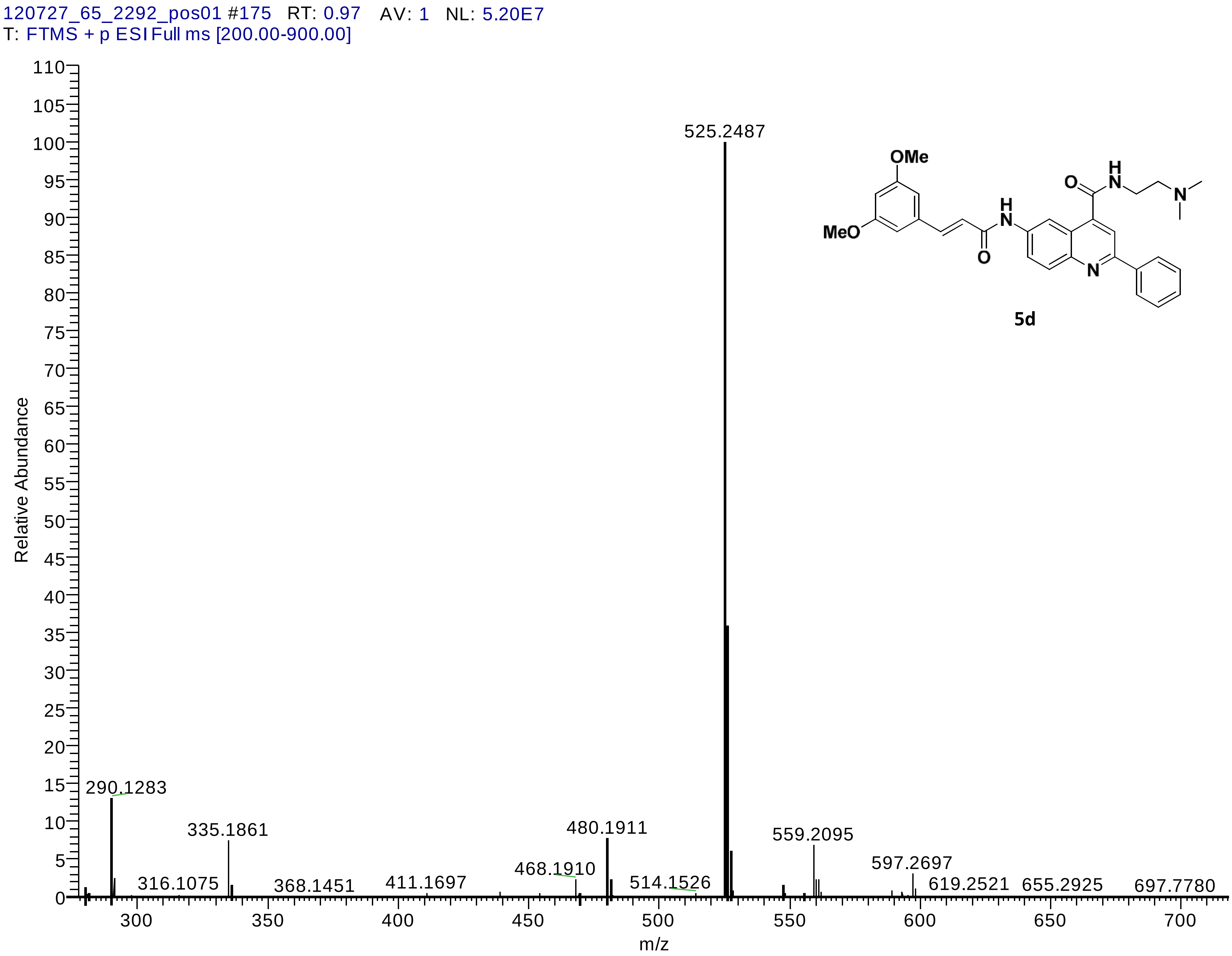
120727_65_2292_pos01 #175 RT: 0.97 AV: 1 NL: 5.20E7
T: FTMS + p ESI Full ms [200.00-900.00]
Relative Abundance
m/z
525.2487
290.1283
335.1861
316.1075
368.1451
411.1697
468.1910
480.1911
514.1526
559.2095
597.2697
619.2521
655.2925
697.7780
OMe
MeO
O
H
N
N
5d

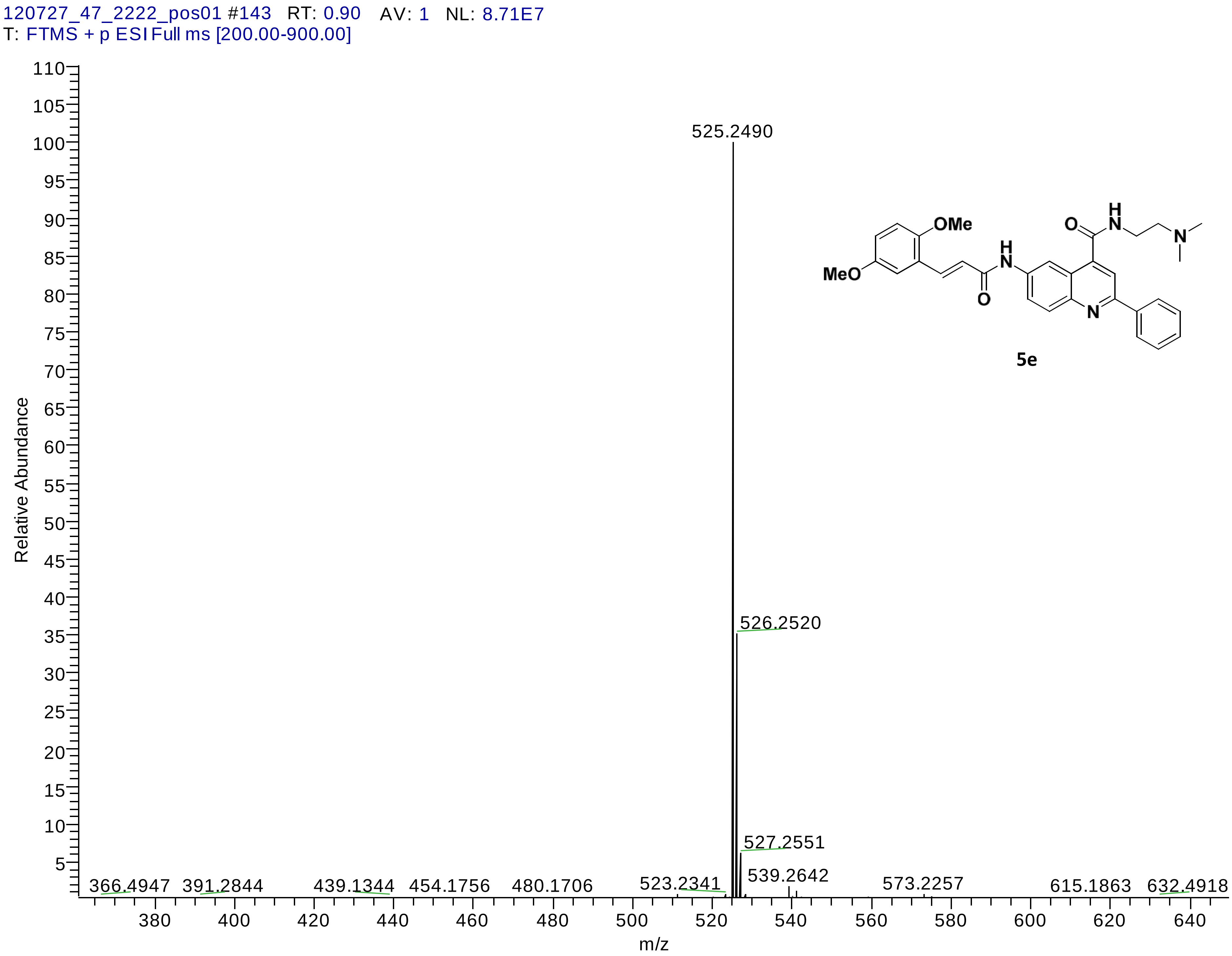
120727_47_2222_pos01 #143 RT: 0.90 AV: 1 NL: 8.71E7
T: FTMS + p ESI Full ms [200.00-900.00]
Relative Abundance
m/z
525.2490
526.2520
527.2551
539.2642
523.2341
573.2257
366.4947
391.2844
439.1344
454.1756
480.1706
615.1863
632.4918
OMe
MeO
O
N
H
5e

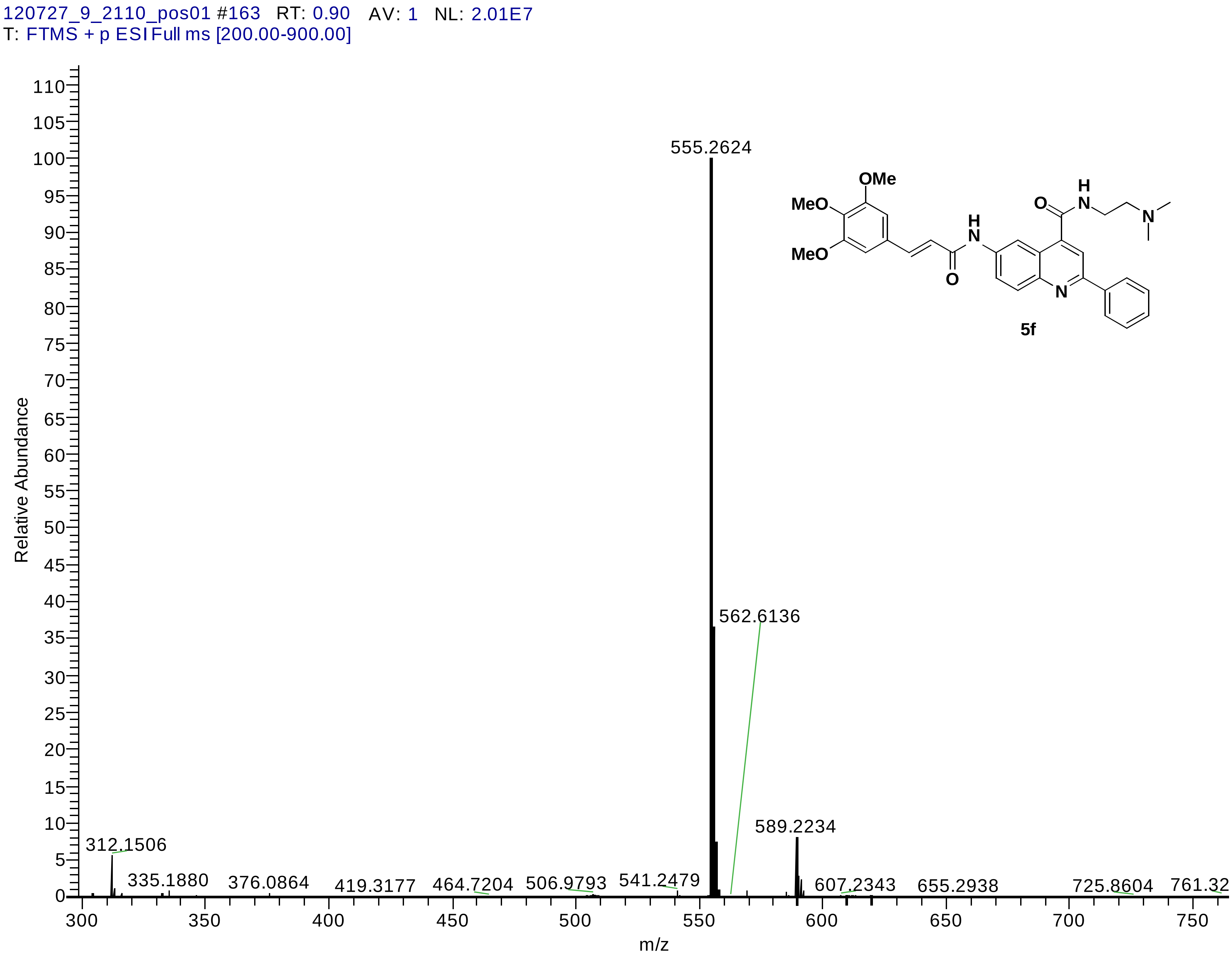
120727_9_2110_pos01 #163 RT: 0.90 AV: 1 NL: 2.01E7
T: FTMS + p ESI Full ms [200.00-900.00]
Relative Abundance
m/z
555.2624
562.6136
589.2234
312.1506
335.1880
376.0864
419.3177
464.7204
506.9793
541.2479
607.2343
655.2938
725.8604
761.32
OMe
MeO
MeO
H
N
O
N
N
5f

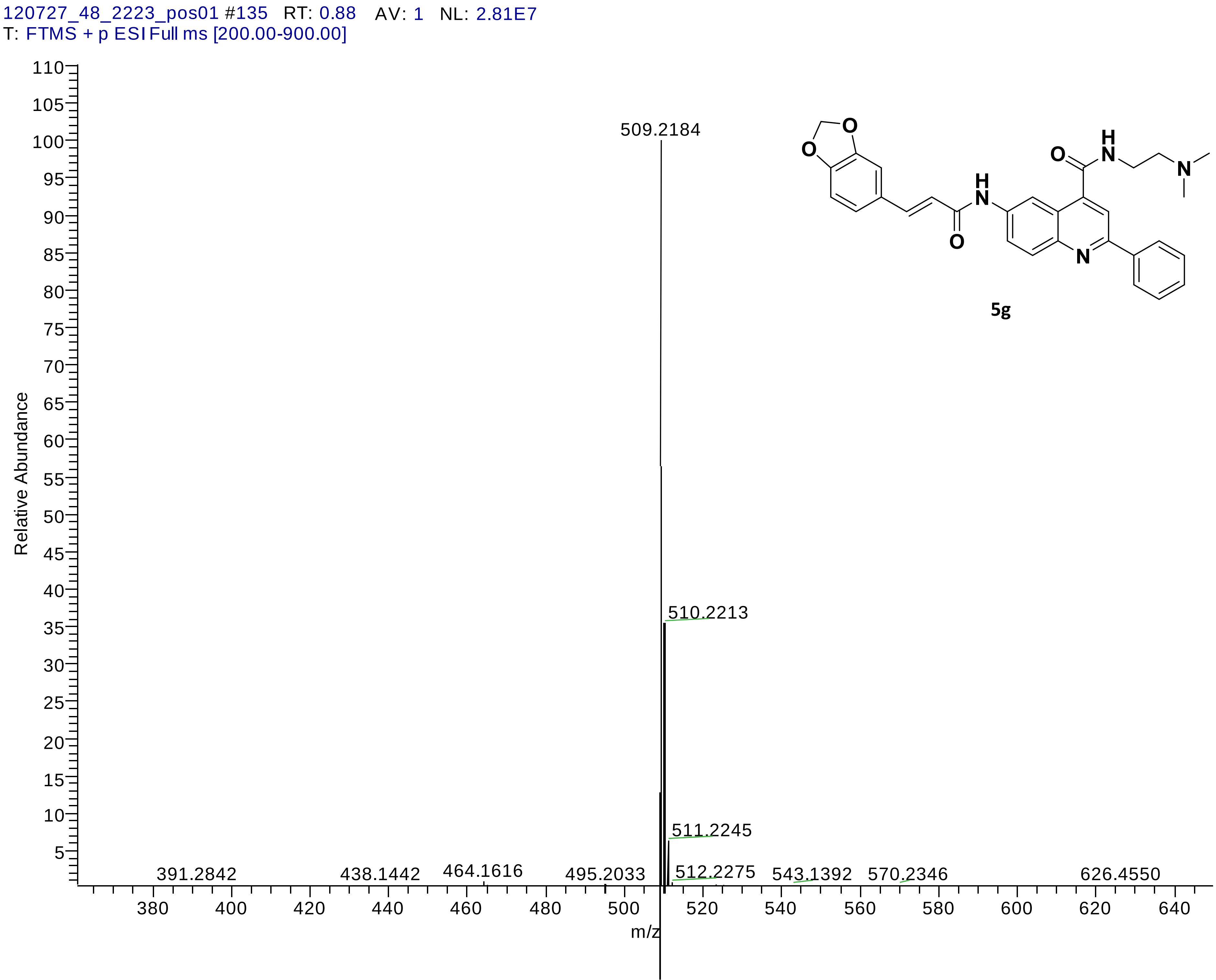
120727_48_2223_pos01 #135 RT: 0.88 AV: 1 NL: 2.81E7
T: FTMS + p ESI Full ms [200.00-900.00]
Relative Abundance
m/z
509.2184
510.2213
511.2245
512.2275
391.2842
438.1442
464.1616
495.2033
543.1392
570.2346
626.4550
O
O
H
N
O
O
H
N
N
N
5g

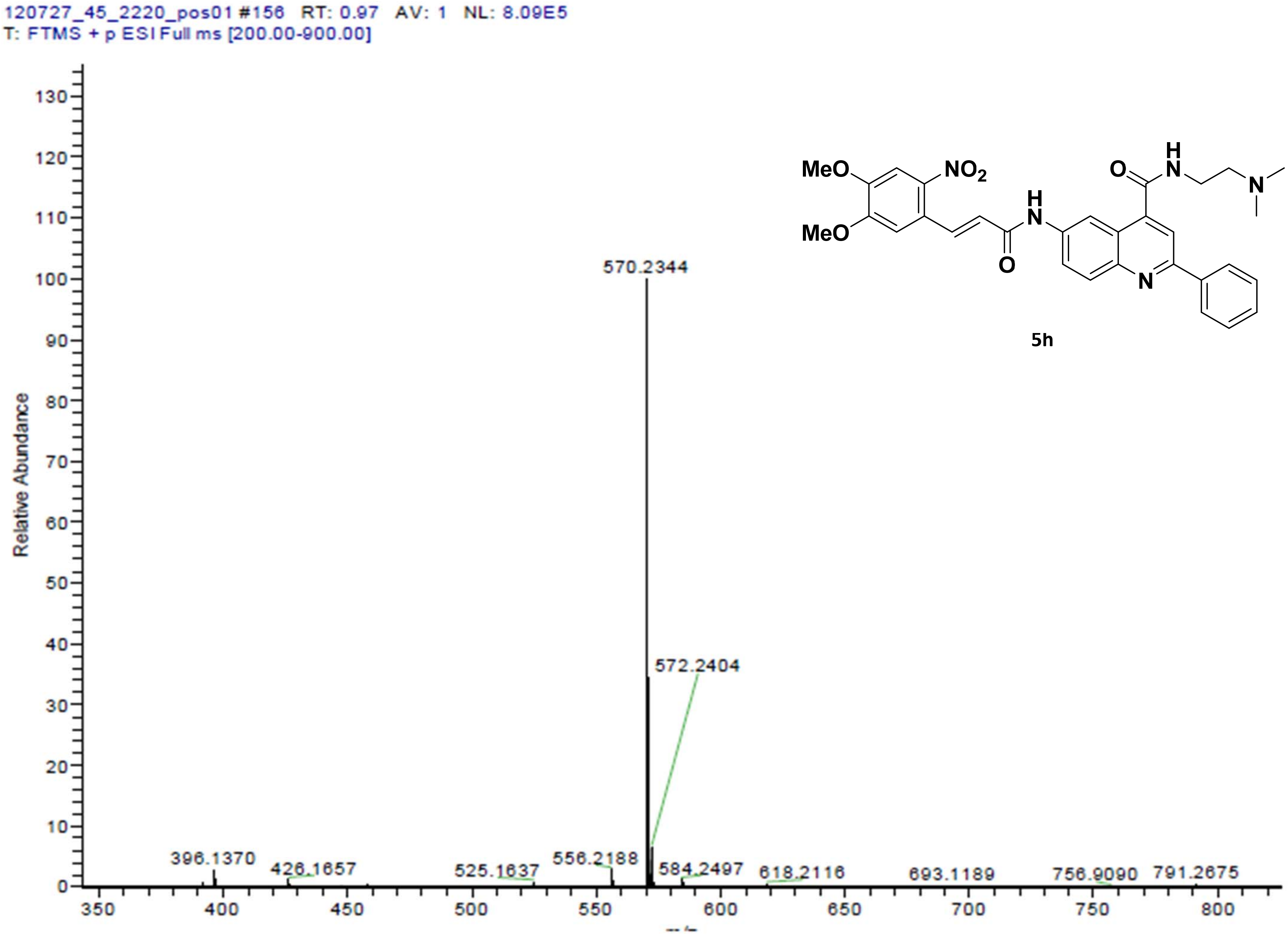
120727_45_2220_pos01 #156 RT: 0.97 AV: 1 NL: 8.09E5
T: FTMS + p ESI Full ms [200.00-900.00]
Relative Abundance
570.2344
572.2404
396.1370
426.1657
525.1637
556.2188
584.2497
618.2116
693.1189
756.9090
791.2675
MeO
MeO
NO2
H
N
O
O
H
N
N
N

5h

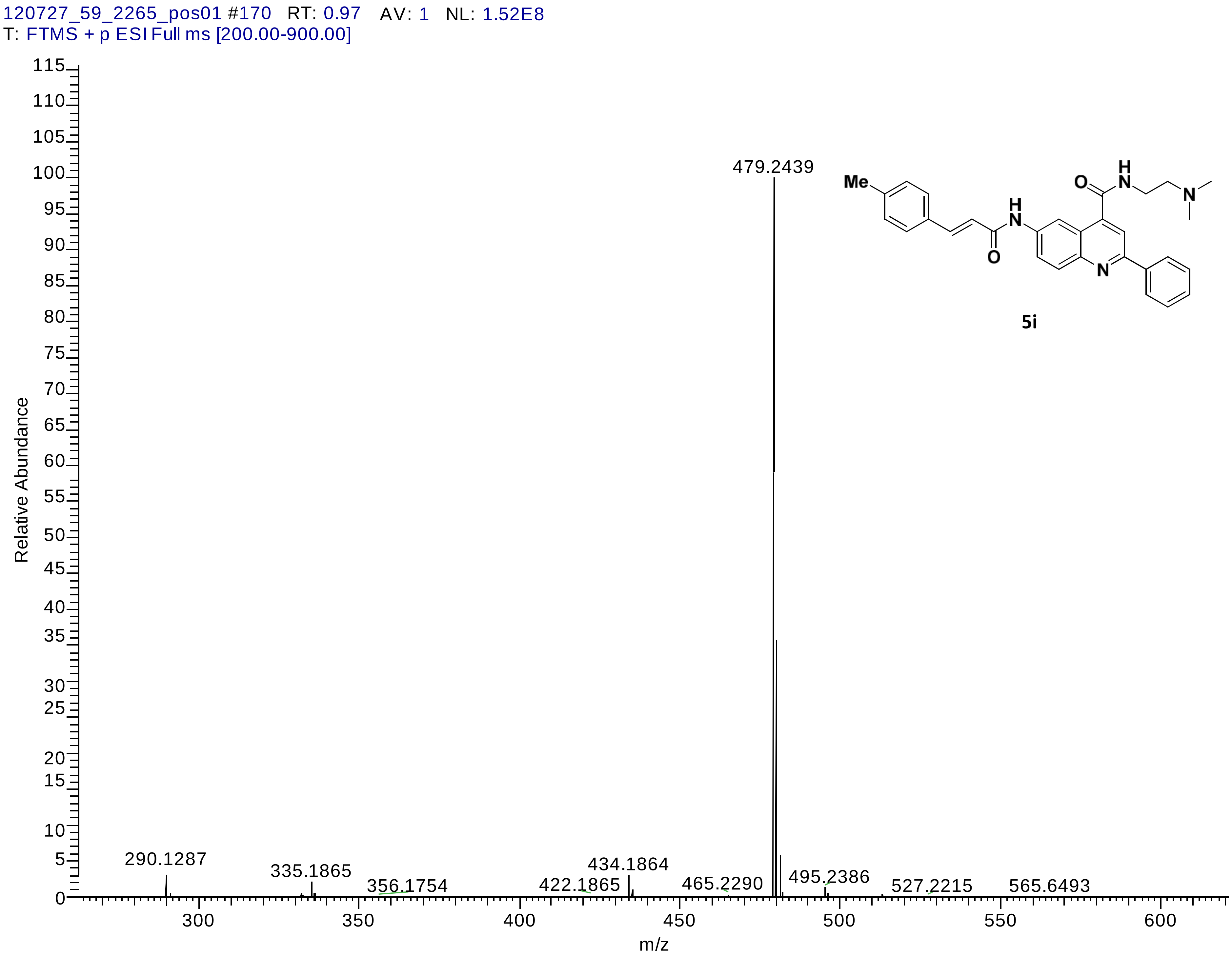
120727_59_2265_pos01 #170 RT: 0.97 AV: 1 NL: 1.52E8
T: FTMS + p ESI Full ms [200.00-900.00]
Relative Abundance
m/z
479.2439
290.1287
335.1865
356.1754
422.1865
434.1864
465.2290
495.2386
527.2215
565.6493
Me
O
H
N
N
5i

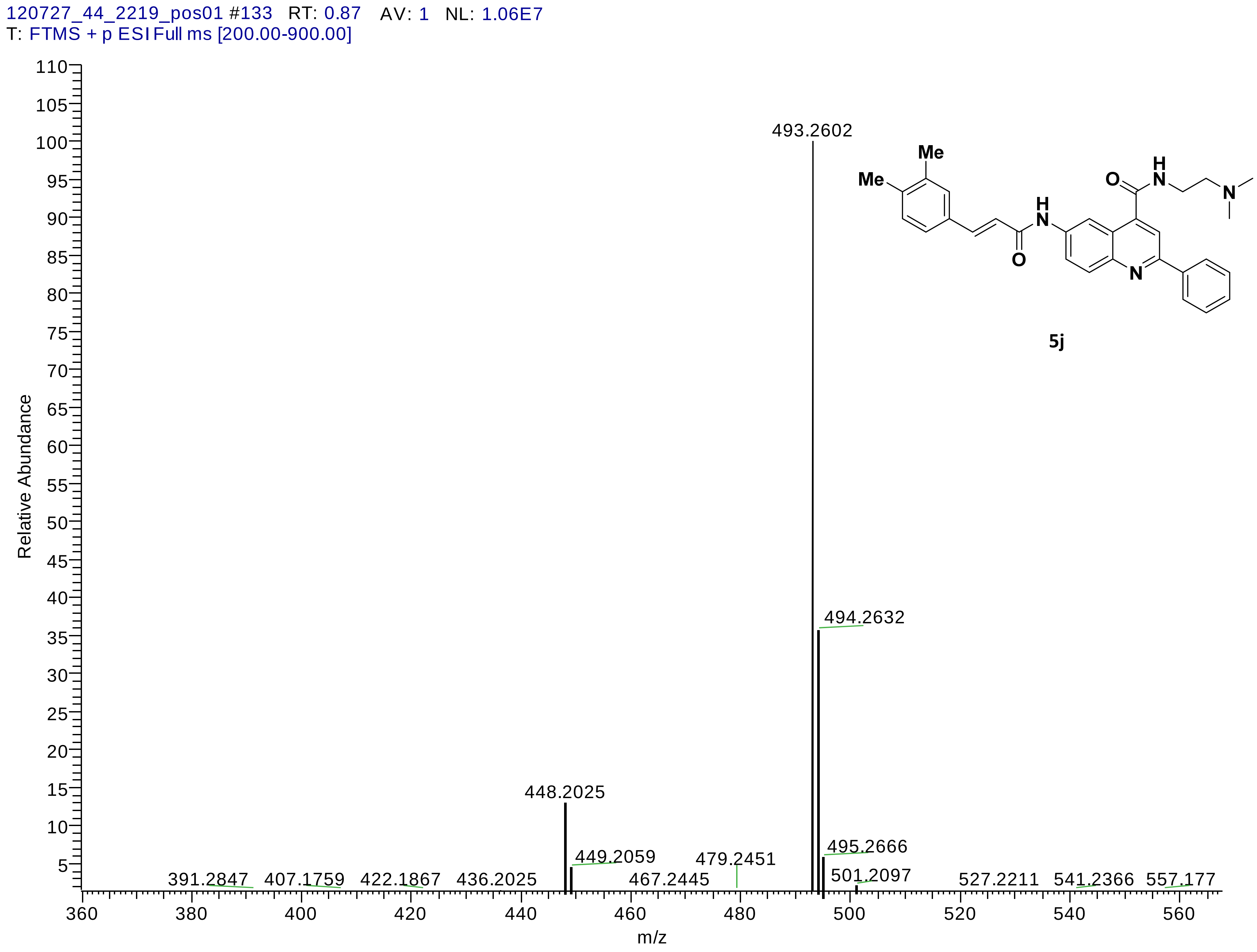
120727_44_2219_pos01 #133 RT: 0.87 AV: 1 NL: 1.06E7
T: FTMS + p ESI Full ms [200.00-900.00]
Relative Abundance
m/z
493.2602
494.2632
495.2666
448.2025
449.2059
479.2451
467.2445
501.2097
391.2847
407.1759
422.1867
436.2025
527.2211
541.2366
557.177
Me
Me
H
N
O
H
N
O
N
N

5j

120727_46_2221_pos01 #129 RT: 0.88 AV: 1 NL: 1.21E7
T: FTMS + p ESI Full ms [200.00-900.00]

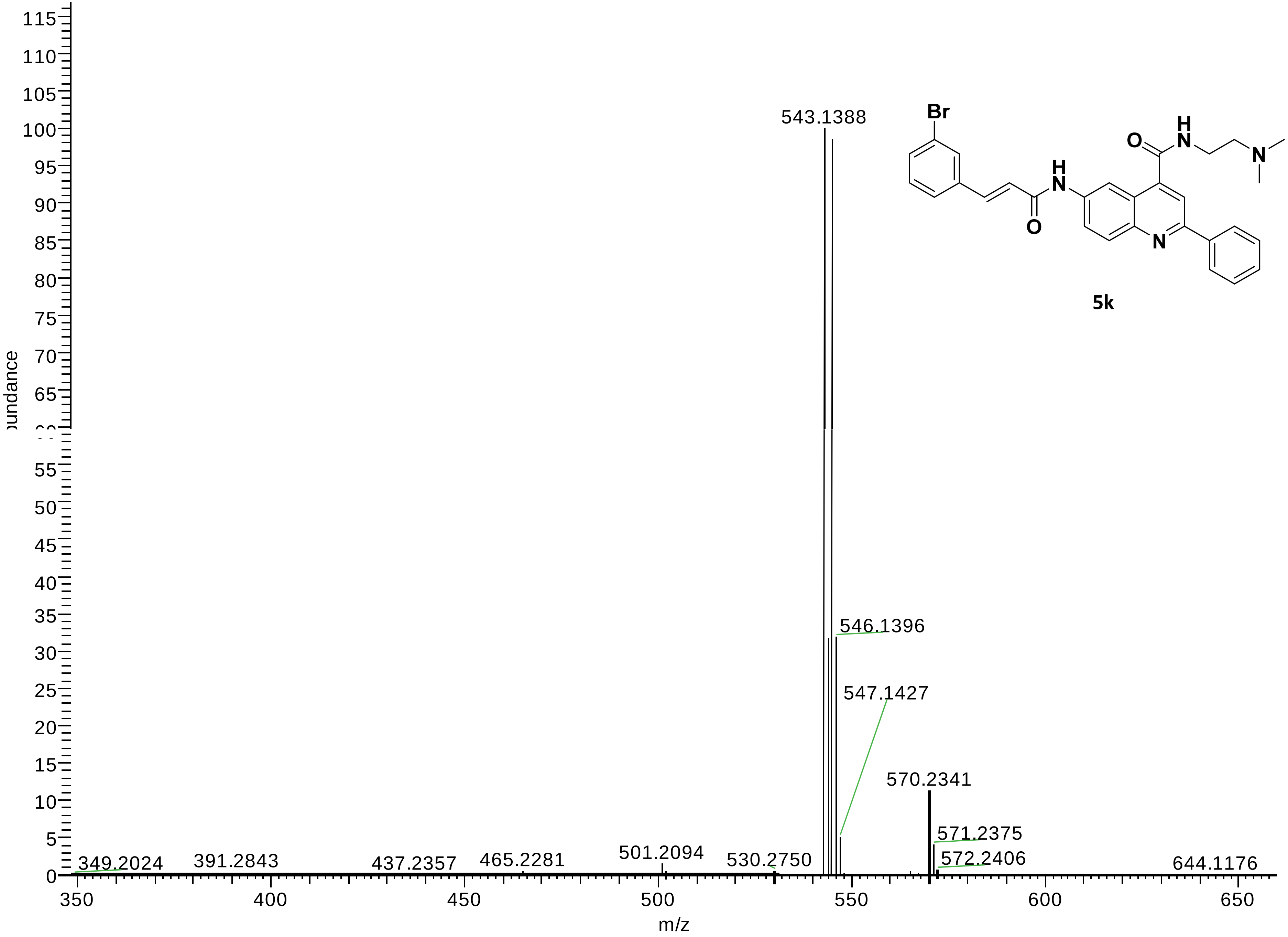

5k

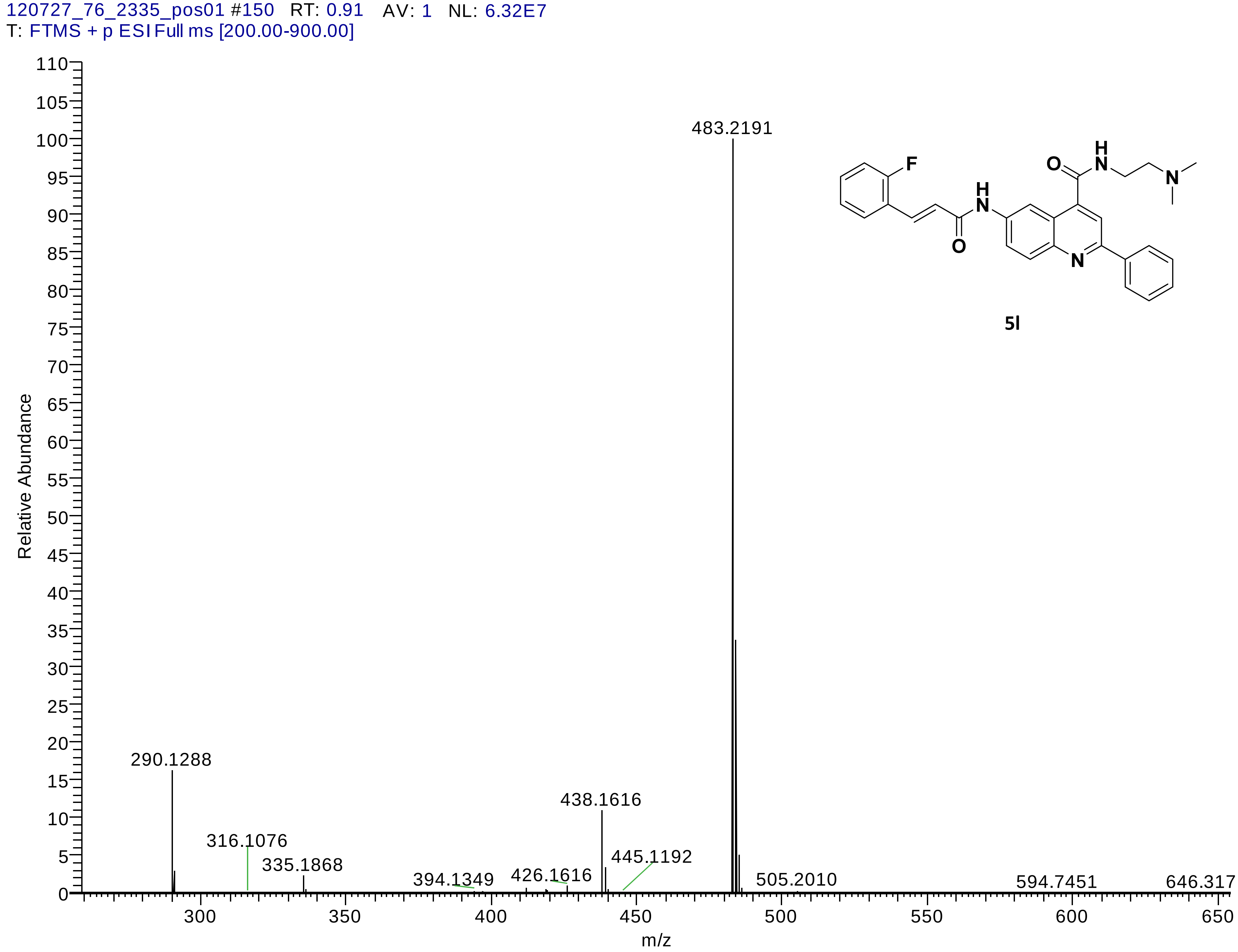
120727_76_2335_pos01 #150 RT: 0.91 AV: 1 NL: 6.32E7
T: FTMS + p ESI Full ms [200.00-900.00]
Relative Abundance
m/z
483.2191
290.1288
316.1076
335.1868
394.1349
426.1616
438.1616
445.1192
505.2010
594.7451
646.317
F
O
H
N
N
O
N
H
N
5l

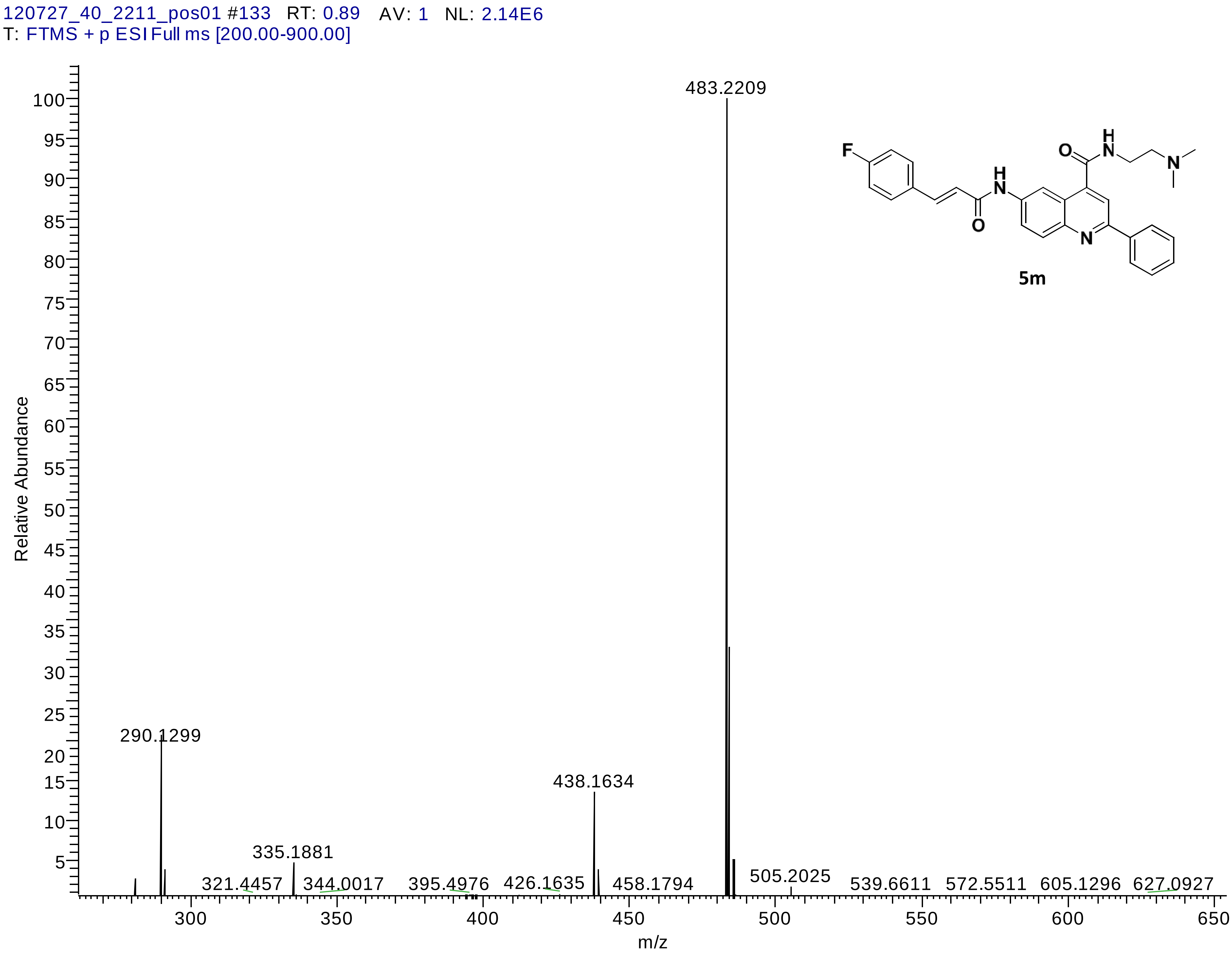
120727_40_2211_pos01 #133 RT: 0.89 AV: 1 NL: 2.14E6
T: FTMS + p ESI Full ms [200.00-900.00]
483.2209
290.1299
438.1634
335.1881
321.4457
344.0017
395.4976
426.1635
458.1794
505.2025
539.6611
572.5511
605.1296
627.0927
Relative Abundance
m/z
5m

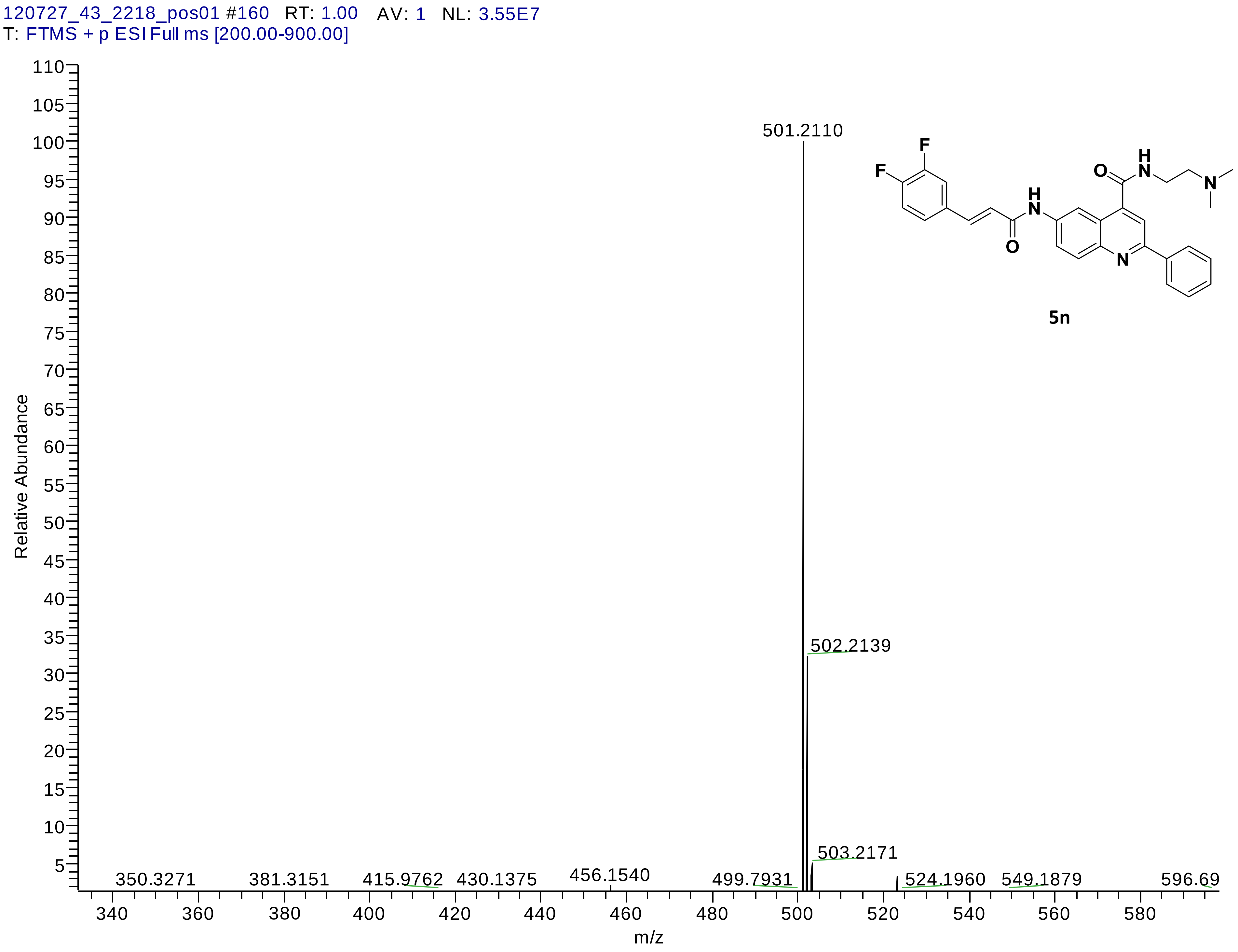
120727_43_2218_pos01 #160 RT: 1.00 AV: 1 NL: 3.55E7
T: FTMS + p ESI Full ms [200.00-900.00]
Relative Abundance
m/z
501.2110
502.2139
503.2171
350.3271
381.3151
415.9762
430.1375
456.1540
499.7931
524.1960
549.1879
596.69
5n

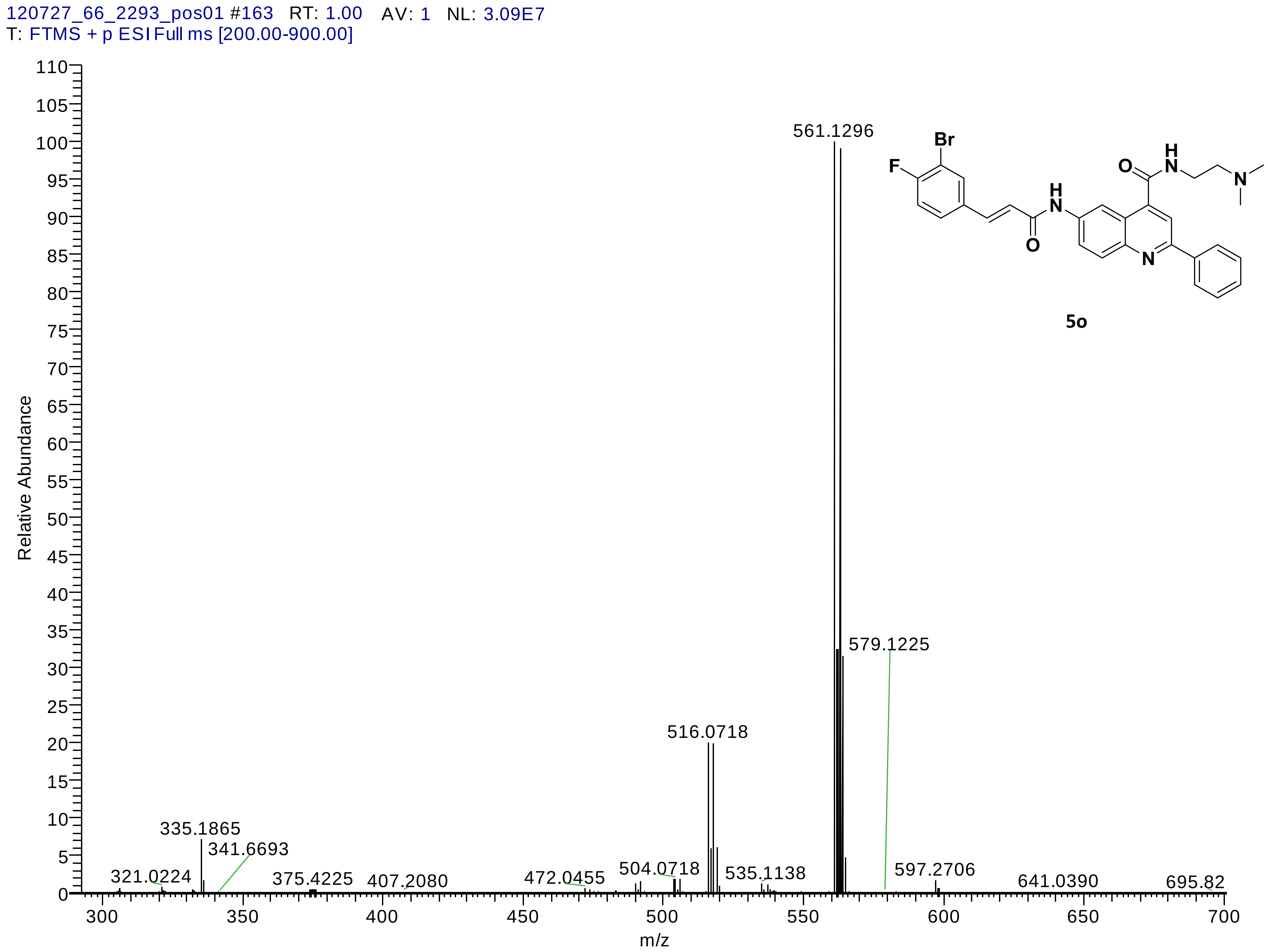

120727_66_2293_pos01 #163 RT: 1.00 AV: 1 NL: 3.09E7
T: FTMS + p ESI Full ms [200.00-900.00]
Relative Abundance
m/z
561.1296
579.1225
516.0718
335.1865
341.6693
321.0224
375.4225
407.2080
472.0455
504.0718
535.1138
597.2706
641.0390
695.82
Br
F
H
N
O
N
5o

120727_77_2336_pos01 #150 RT: 0.96 AV: 1 NL: 1.97E6
T: FTMS + p ESI Full ms [200.00-900.00]

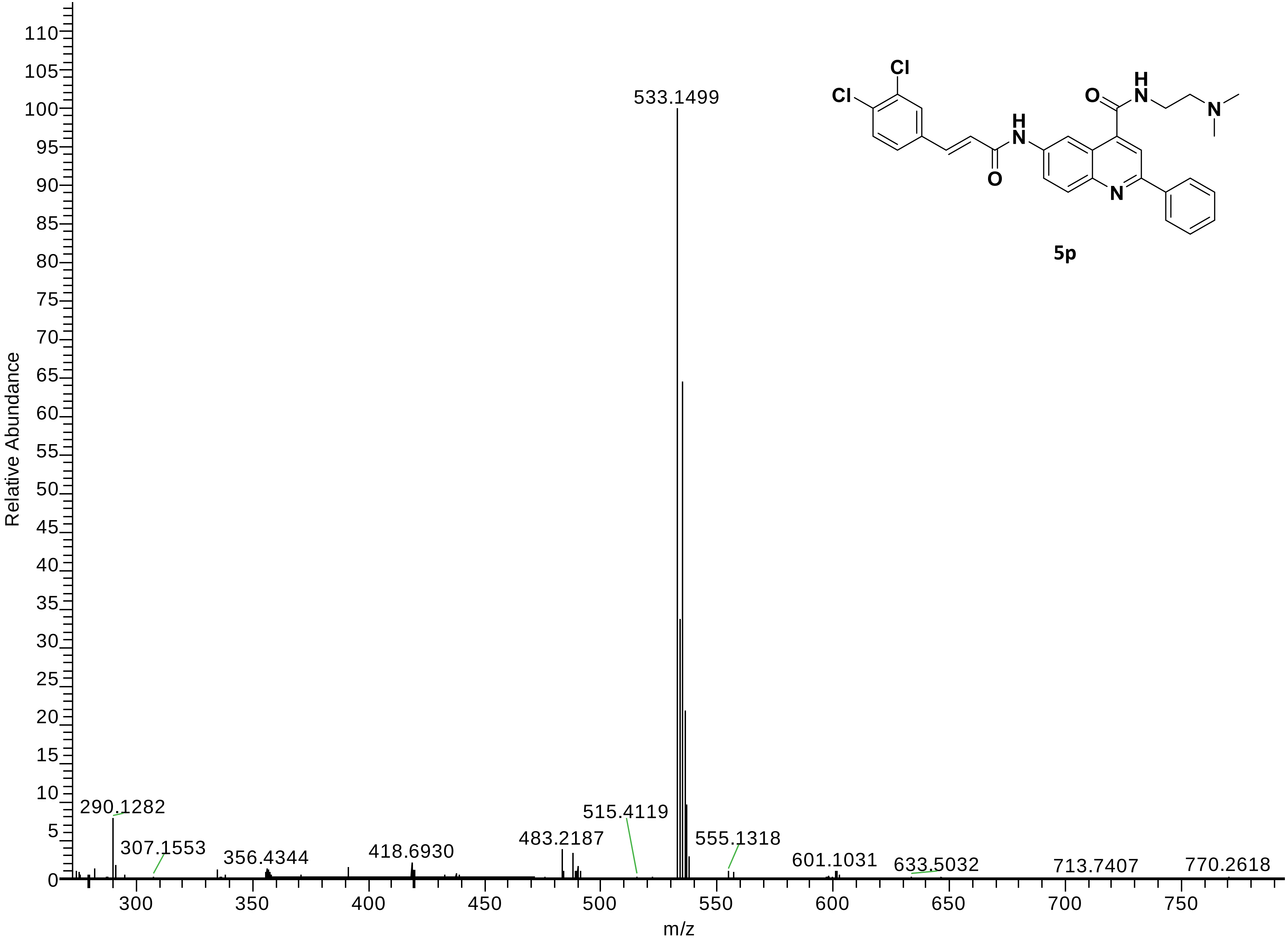

5p

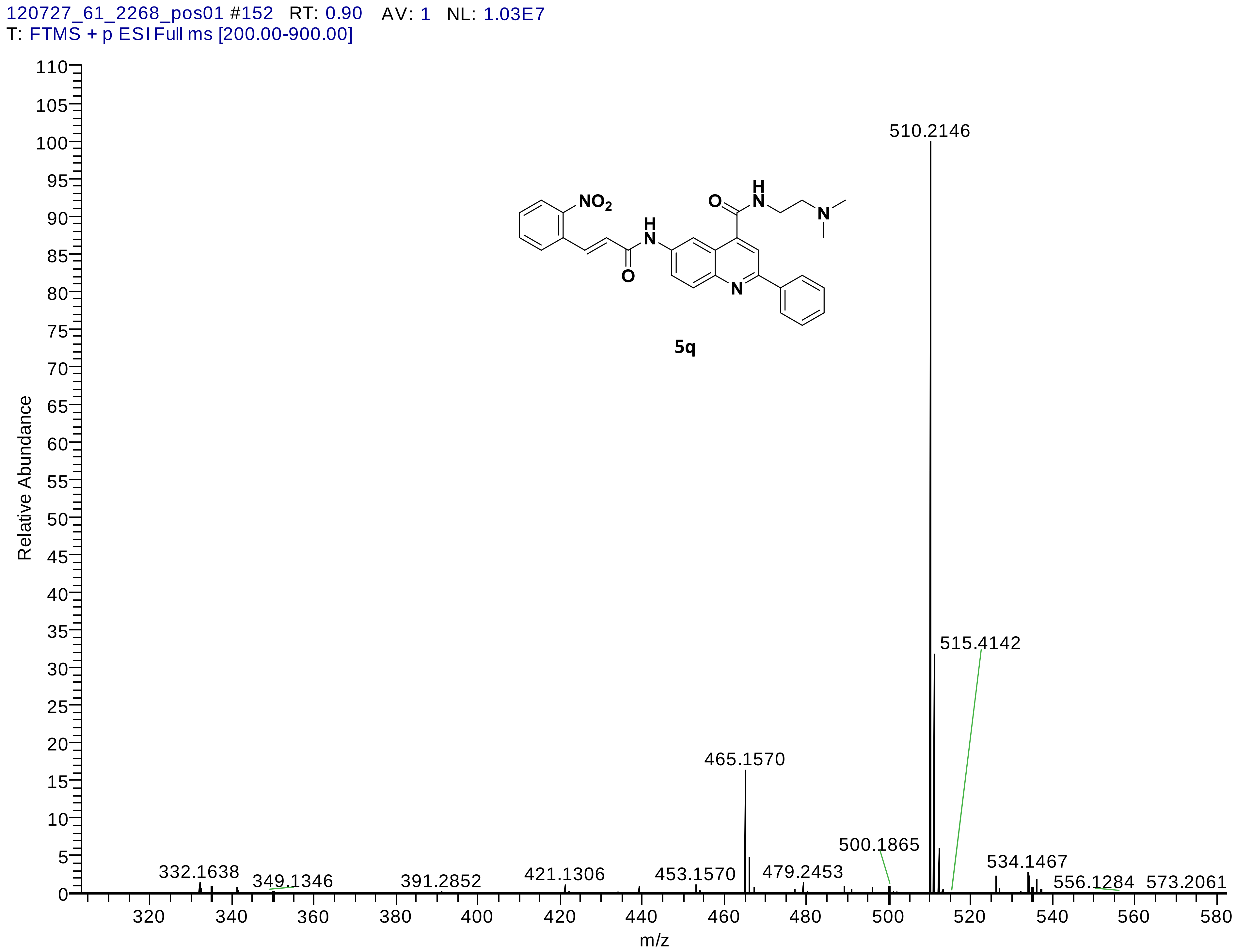
120727_61_2268_pos01 #152 RT: 0.90 AV: 1 NL: 1.03E7
T: FTMS + p ESI Full ms [200.00-900.00]
Relative Abundance
m/z
510.2146
515.4142
465.1570
500.1865
534.1467
332.1638
349.1346
391.2852
421.1306
453.1570
479.2453
556.1284
573.2061
NO2
H
N
O
N
H
N
N
O
5q

120727_10_2112_pos01 #147 RT: 0.88 AV: 1 NL: 3.44E7
T: FTMS + p ESI Full ms [200.00-900.00]

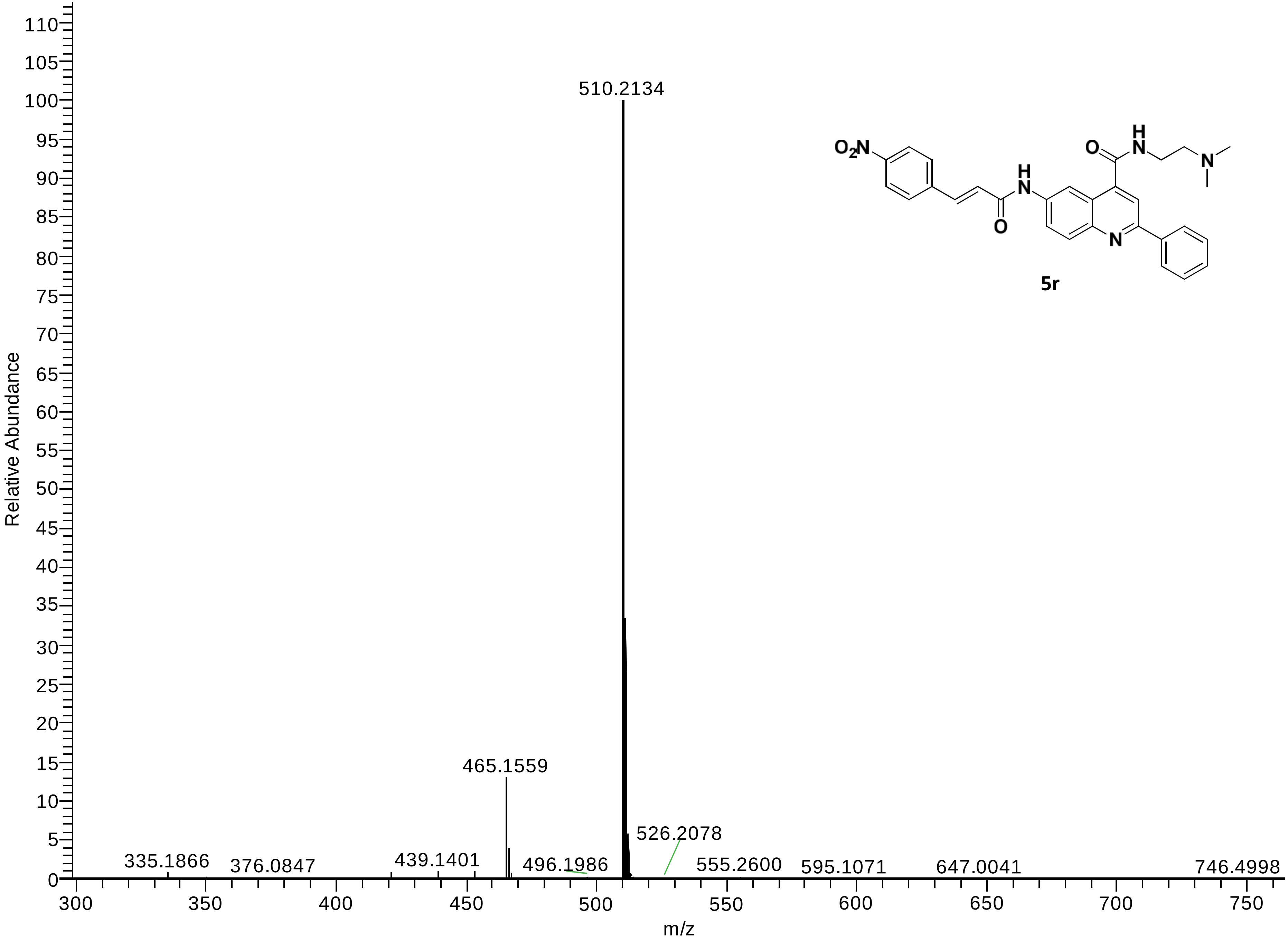

Supplement: Supplementary file 2 [file oncotarget-07-38078-s002.pdf]
