# Supplementary material for: Derivatives of 6-cinnamamido-quinoline-4-carboxamide impair lysosome function and induce apoptosis
Source: Oncotarget. 2016 May 13;7(25):38078–90. doi: 10.18632/oncotarget.9348 (PMC5122373; doi:10.18632/oncotarget.9348)
Supplement: Supplementary file 3 [file oncotarget-07-38078-s003.pdf]

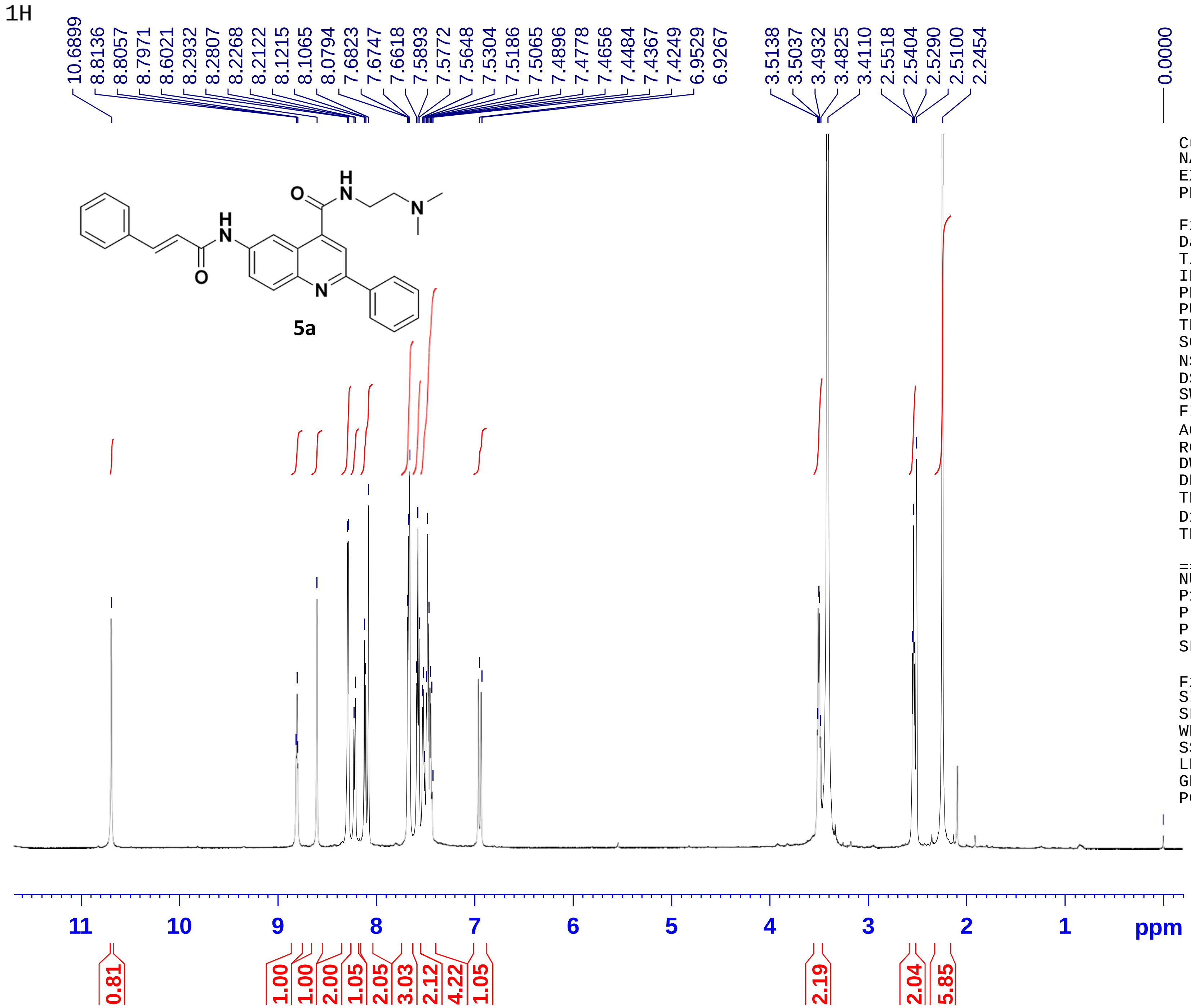

Current Data Parameters	
NAME	RK-II-187-23
EXPNO	1
PROCNO	1
F2 - Acquisition Parameters	
Date_	20120305
Time	17.09
INSTRUM	spect
PROBHD	5 mm CPTCI 1H-
PULPROG	zg
TD	32768
SOLVENT	DMSO
NS	1
DS	0
SWH	8389.262 Hz
FIDRES	0.256020 Hz
AQ	1.9530228 sec
RG	18
DW	59.600 usec
DE	6.50 usec
TE	298.0 K
D1	2.00000000 sec
TD0	1
======== CHANNEL f1 ========	
NUC1	1H
P1	9.25 usec
PL1	-1.00 dB
PL1W	3.00416374 W
SFO1	600.1328170 MHz
F2 - Processing parameters	
SI	32768
SF	600.1300011 MHz
WDW	no
SSB	0
LB	0 Hz
GB	0
PC	1.00

13C

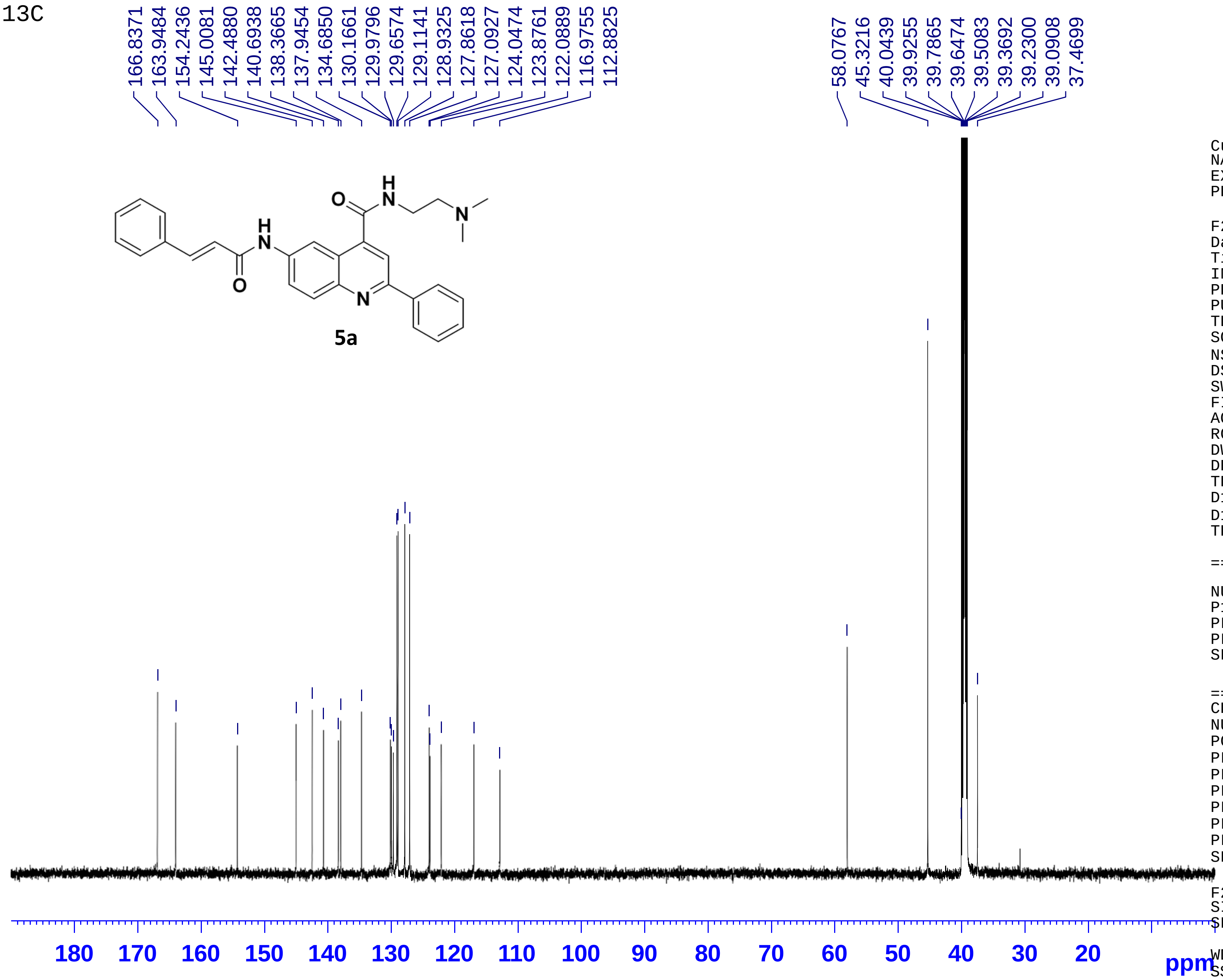

BRUKER

Current Data Parameters
NAME RK-II-187-23-13C
EXPNO 1
PROCNO 1

F2 - Acquisition Parameters
Date_ 20120305
Time 17.13
INSTRUM spect
PROBHD 5 mm CPTCI 1H-
PULPROG zgpg30
TD 65536
SOLVENT DMSO
NS 194
DS 4
SWH 36231.883 Hz
FIDRES 0.552855 Hz
AQ 0.9044468 sec
RG 6502
DW 13.800 usec
DE 6.50 usec
TE 298.0 K
D1 2.00000000 sec
D11 0.03000000 sec
TD0 1

======== CHANNEL f1 ========
NUC1 13C
P1 15.00 usec
PL1 -2.80 dB
PL1W 79.45259094 W
SFO1 150.9178993 MHz

======== CHANNEL f2 ========
CPDPRG2 waltz16
NUC2 1H
PCPD2 80.00 usec
PL2 -5.40 dB
PL12 10.70 dB
PL13 120.00 dB
PL2W 8.27415466 W
PL12W 0.20310640 W
PL13W 0 W
SFO2 600.1324005 MHz

F2 - Processing parameters
SI 32768
SF 150.9028741 MHz
WDW EM
SSB 0
LB 1.00 Hz
GB 0
PC 1.40

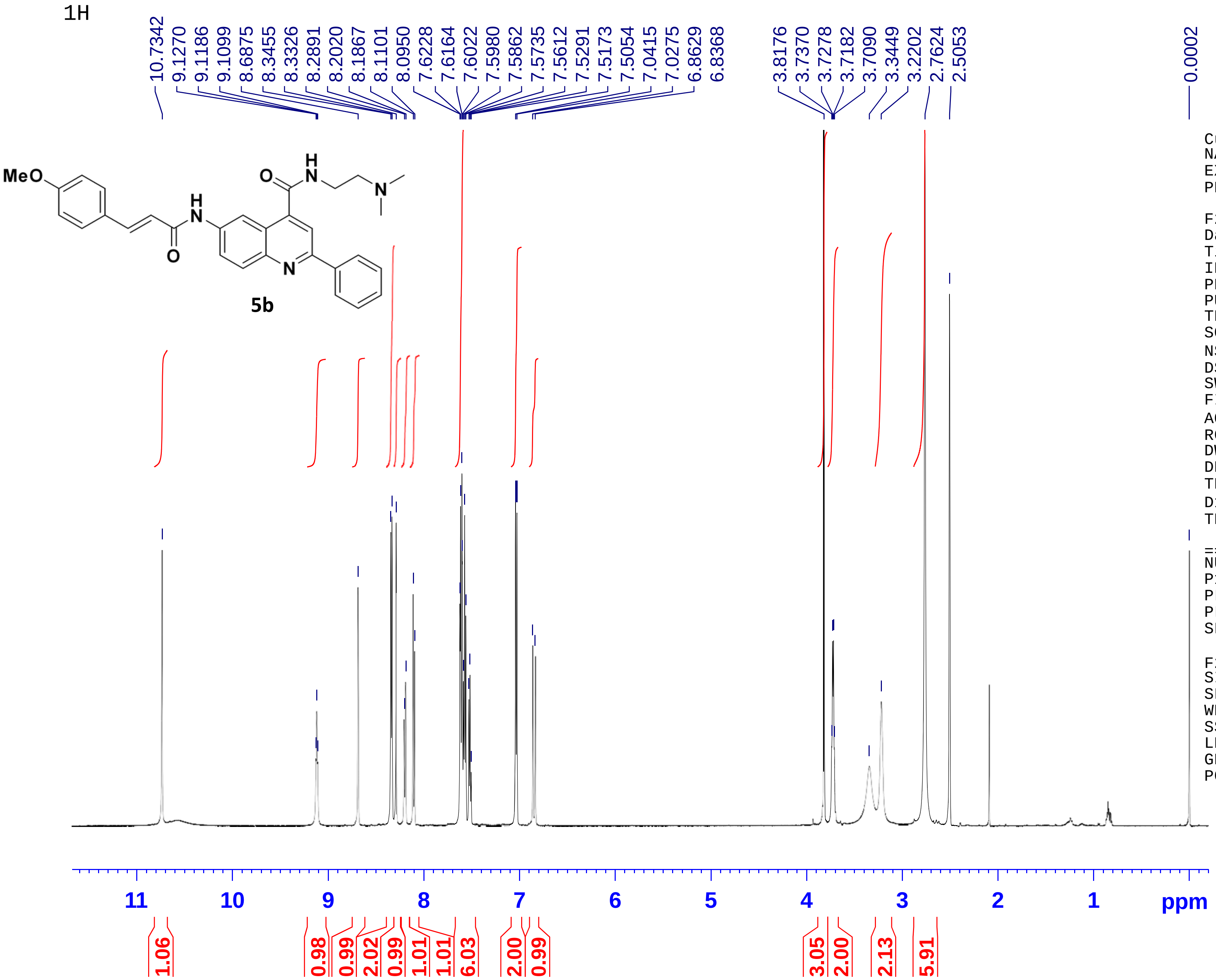


```
Current Data Parameters
NAME              RK-II-199-23
EXPNO                        1
PROCNO                       1

F2 - Acquisition Parameters
Date_                 20120306
Time                     14.38
INSTRUM                  spect
PROBHD      5 mm CPTCI 1H-
PULPROG                     zg
TD                       32768
SOLVENT                   DMSO
NS                           1
DS                           0
SWH                   8389.262 Hz
FIDRES                0.256020 Hz
AQ                   1.9530228 sec
RG                          18
DW                      59.600 usec
DE                        6.50 usec
TE                       298.2 K
D1                  2.00000000 sec
TD0                          1

======== CHANNEL f1 ========
NUC1                        1H
P1                        9.25 usec
PL1                      -1.00 dB
PL1W                3.00416374 W
SFO1             600.1328170 MHz

F2 - Processing parameters
SI                       32768
SF               600.1300043 MHz
WDW                         no
SSB          0
LB           0 Hz
GB           0
PC                        1.00
```

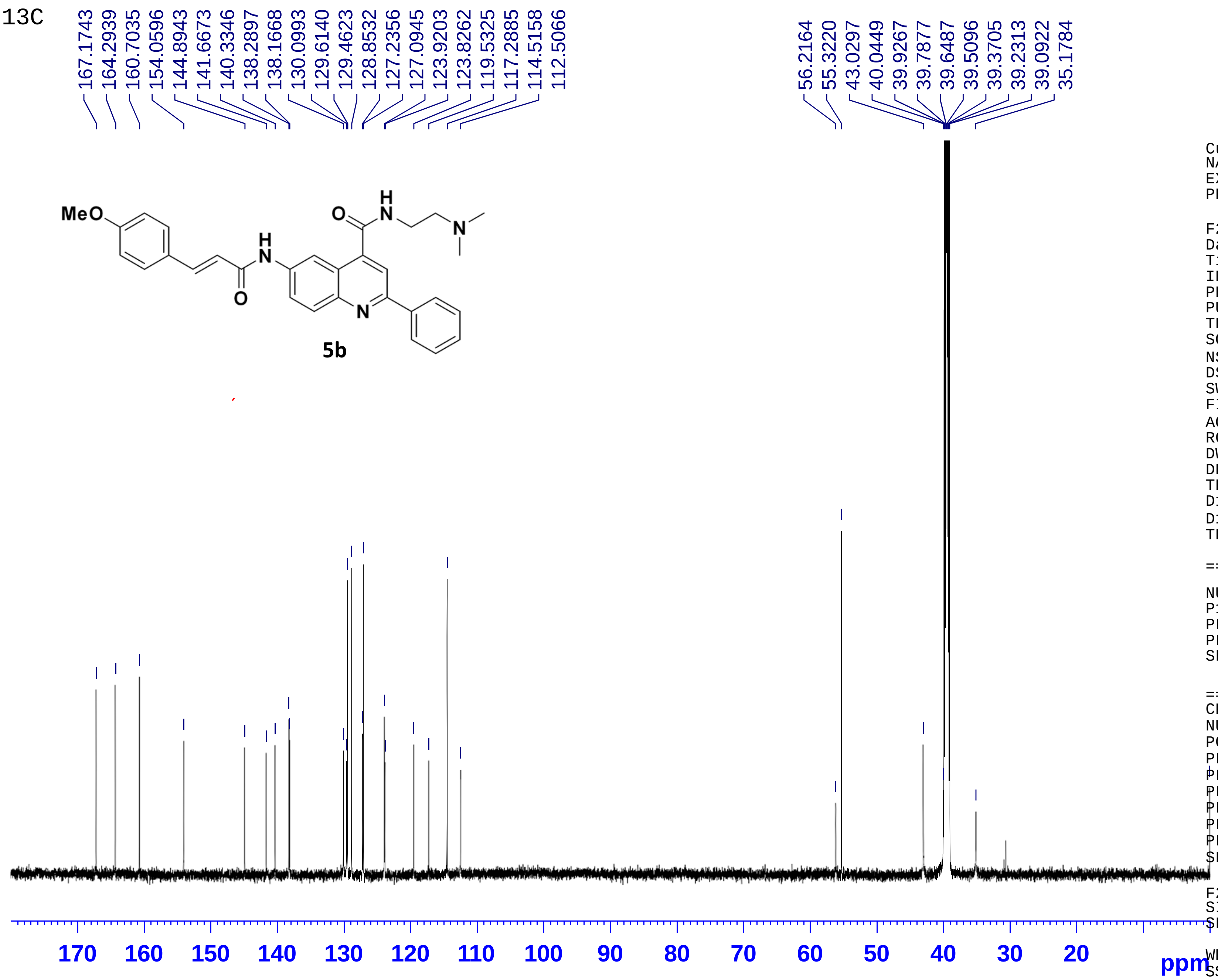


```
Current Data Parameters
NAME          RK-II-199-23-13C
EXPNO                        1
PROCNO                       1

F2 - Acquisition Parameters
Date_               20120306
Time                   14.42
INSTRUM                spect
PROBHD     5 mm CPTCI 1H-
PULPROG              zgpg30
TD                     65536
SOLVENT                 DMSO
NS                       336
DS                         4
SWH               36231.883 Hz
FIDRES             0.552855 Hz
AQ                0.9044468 sec
RG                    5792.6
DW                    13.800 usec
DE                    100.00 usec
TE                     298.0 K
D1                2.00000000 sec
D11               0.03000000 sec
TD0                        1

======== CHANNEL f1 ========
NUC1                     13C
P1                     15.00 usec
PL1                    -2.80 dB
PL1W             79.45259094 W
SFO1             150.9178993 MHz

======== CHANNEL f2 ========
CPDPRG2              waltz16
NUC2                      1H
PCPD2                  80.00 usec
PL2                    -5.40 dB
PL12                   10.70 dB
PL13                  120.00 dB
PL2W              8.27415466 W
PL12W             0.20310640 W
PL13W          0 W
SFO2             600.1324005 MHz

F2 - Processing parameters
SI                     32768
SF               150.9028819 MHz
WDW            0          EM
SSB
LB             0
GB                      1.00 Hz
PC
                        1.40
```

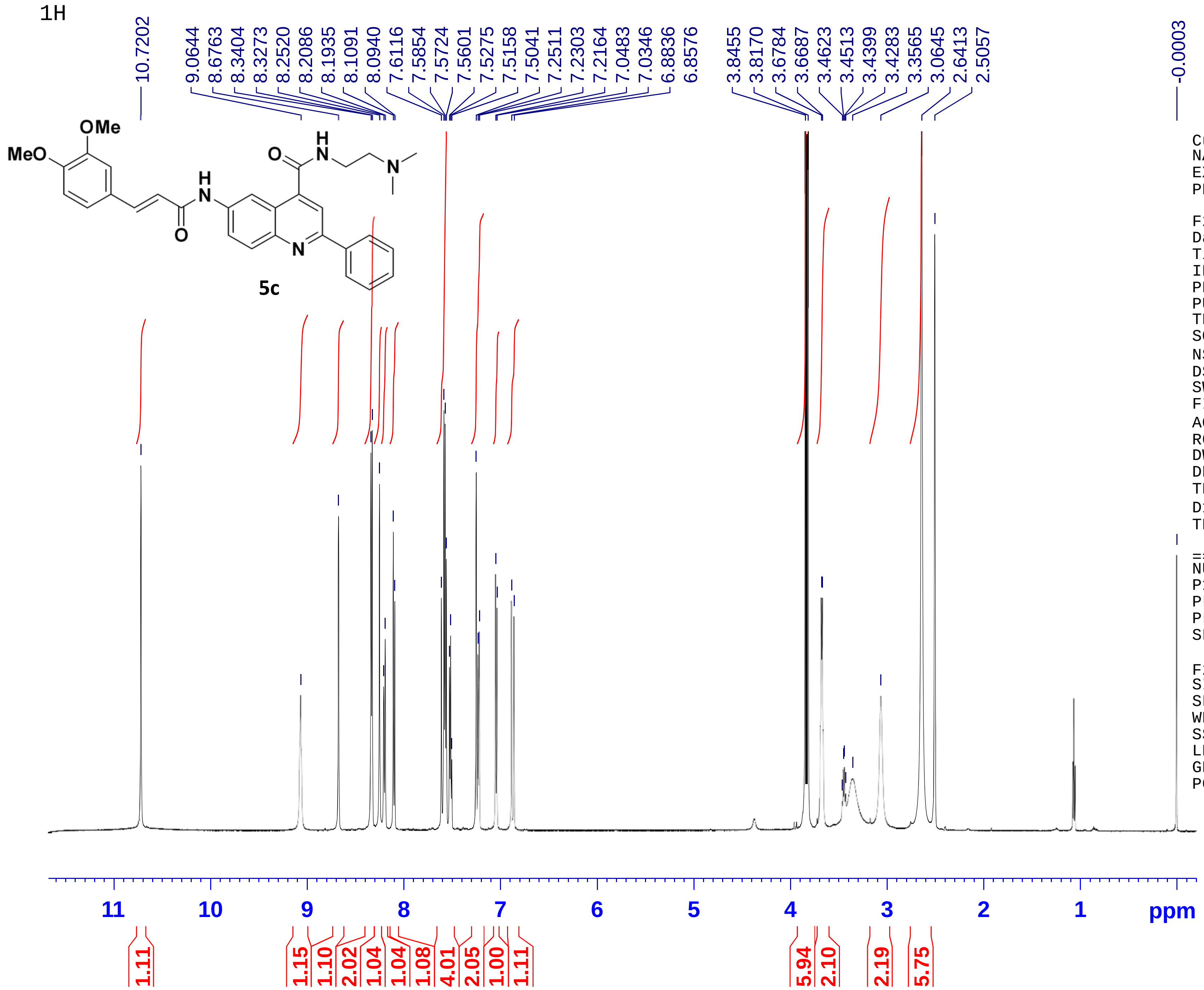


```
Current Data Parameters
NAME          RK-II-185-25
EXPNO                    1
PROCNO                   1

F2 - Acquisition Parameters
Date_             20120306
Time                 15.05
INSTRUM              spect
PROBHD    5 mm CPTCI 1H-
PULPROG                 zg
TD                   32768
SOLVENT               DMSO
NS                       1
DS                       0
SWH               8389.262 Hz
FIDRES            0.256020 Hz
AQ               1.9530228 sec
RG                      18
DW                  59.600 usec
DE                    6.50 usec
TE                   298.0 K
D1              2.00000000 sec
TD0                      1

======== CHANNEL f1 ========
NUC1                    1H
P1                    9.25 usec
PL1                  -1.00 dB
PL1W            3.00416374 W
SFO1           600.1328170 MHz

F2 - Processing parameters
SI                   32768
SF             600.1300036 MHz
WDW                     no
SSB        0
LB         0 Hz
GB         0
PC                    1.00
```

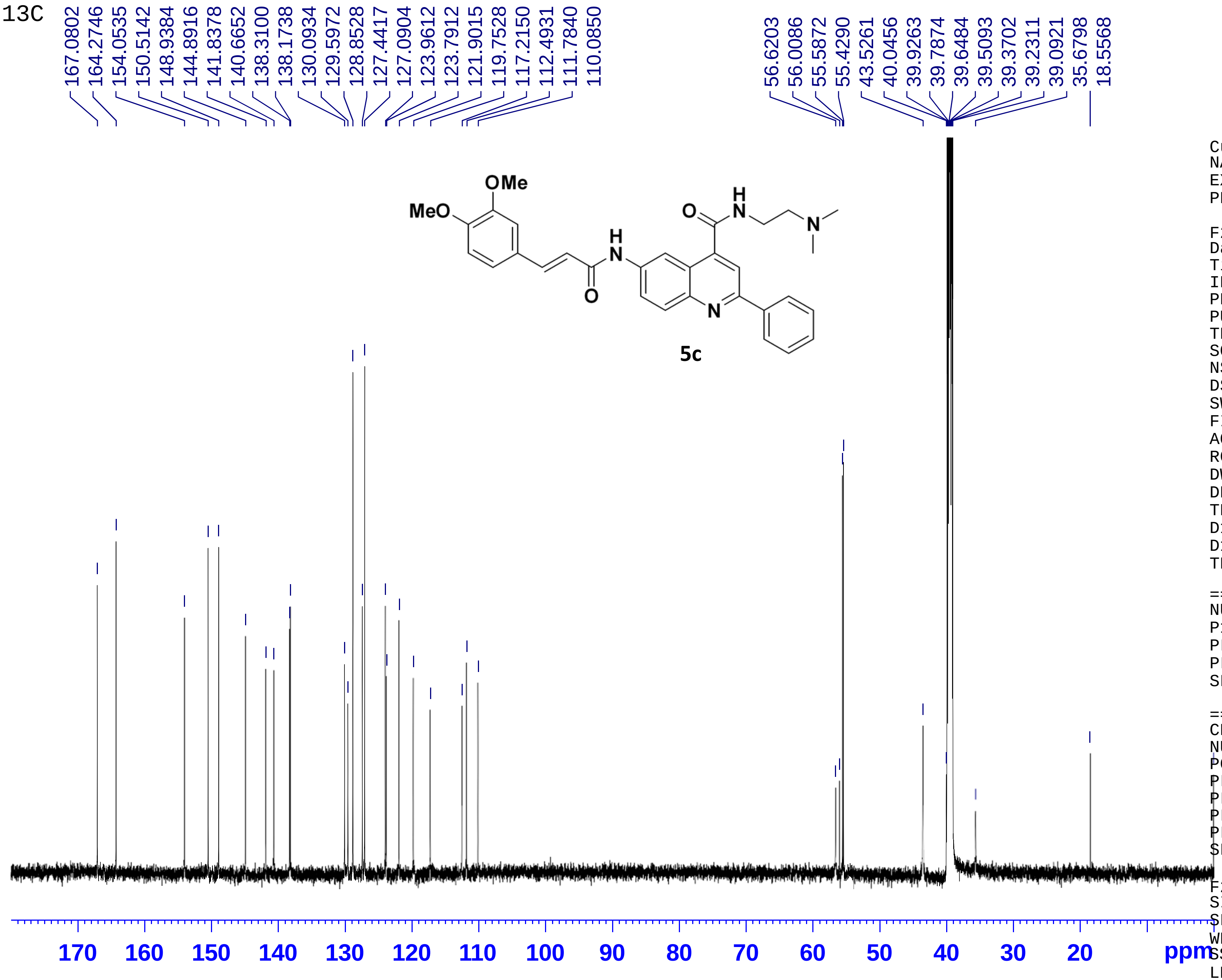

Current Data Parameters
NAME RK-II-185-25-13C
EXPNO 1
PROCNO 1

F2 - Acquisition Parameters
Date_ 20120306
Time 15.08
INSTRUM spect
PROBHD 5 mm CPTCI 1H-
PULPROG zgig30
TD 65536
SOLVENT DMSO
NS 408
DS 4
SWH 36231.883 Hz
FIDRES 0.552855 Hz
AQ 0.9044468 sec
RG 5160.6
DW 13.800 usec
DE 6.50 usec
TE 298.0 K
D1 2.00000000 sec
D11 0.03000000 sec
TD0 1

======== CHANNEL f1 ========
NUC1 13C
P1 15.00 usec
PL1 -2.80 dB
PL1W 79.45259094 W
SFO1 150.9178993 MHz

======== CHANNEL f2 ========
CPDPRG2 waltz16
NUC2 1H
PCPD2 80.00 usec
PL2 -5.40 dB
PL12 10.70 dB
PL2W 8.27415466 W
PL12W 0.20310640 W
SFO2 600.1324005 MHz

F2 - Processing parameters
SI 32768
SF 150.9028815 MHz
WDW EM
SSB 0
LB 1.00 Hz
GB 0
PC 1.40

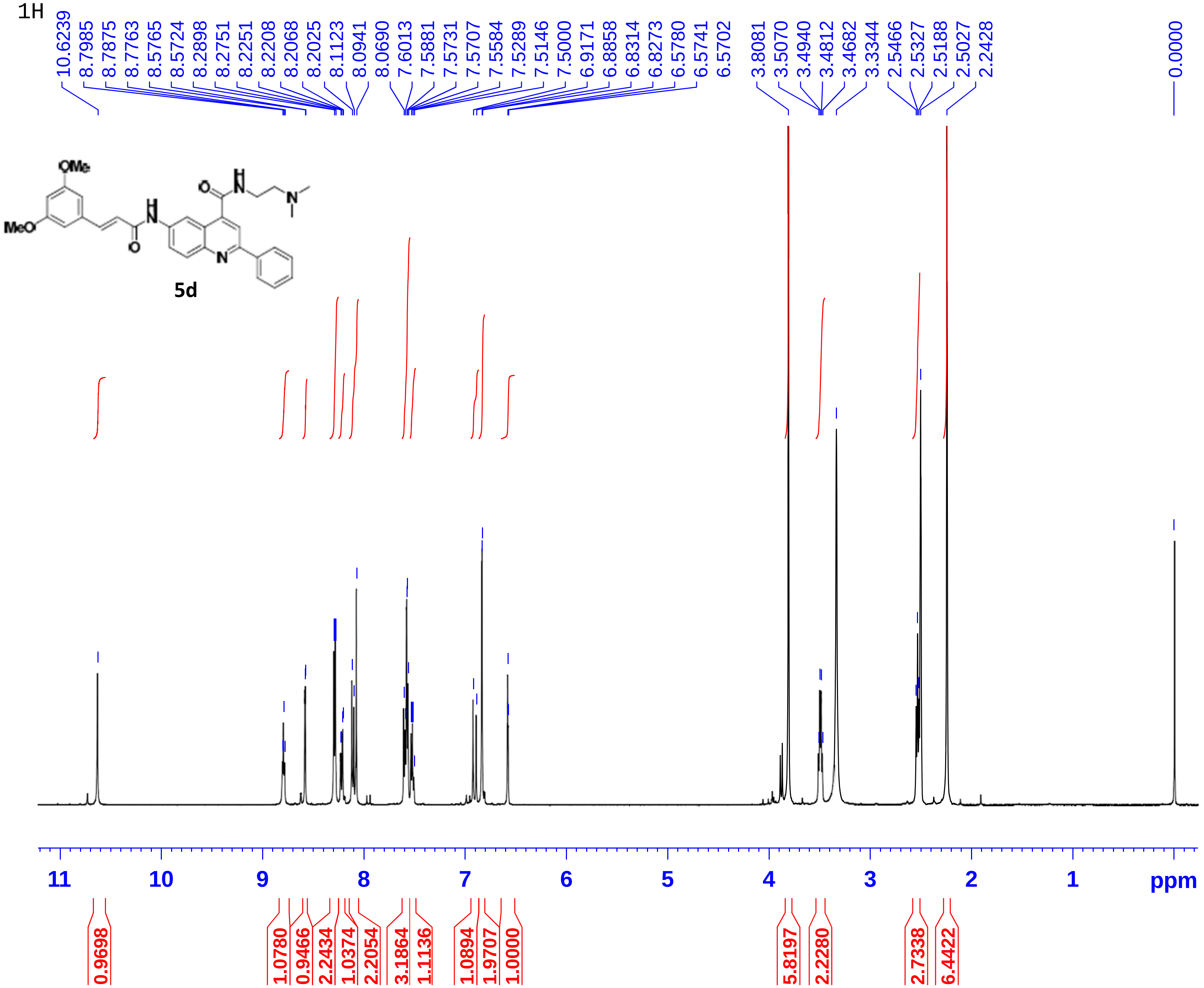

5d

Current Data Parameters
NAME RK-III-30-20
EXPNO 1
PROCNO 1

F2 - Acquisition Parameters
Date_ 20120611
Time 15.51
INSTRUM spect
PROBHD 5 mm CPTXI 1H-
PULPROG zg
TD 32768
SOLVENT DMSO
NS 1
DS 0
SWH 7002.801 Hz
FIDRES 0.213709 Hz
AQ 2.3396852 sec
RG 18
DW 71.400 usec
DE 6.50 usec
TE 298.1 K
D1 2.00000000 sec
TD0 1

======== CHANNEL f1 ========
NUC1 1H
P1 9.25 usec
PL1 -1.00 dB
PL1W 2.30846262 W
SFO1 500.1323476 MHz

F2 - Processing parameters
SI 32768
SF 500.1300033 MHz
WDW no
SSB 0
LB 0 Hz
GB 0
PC 1.00

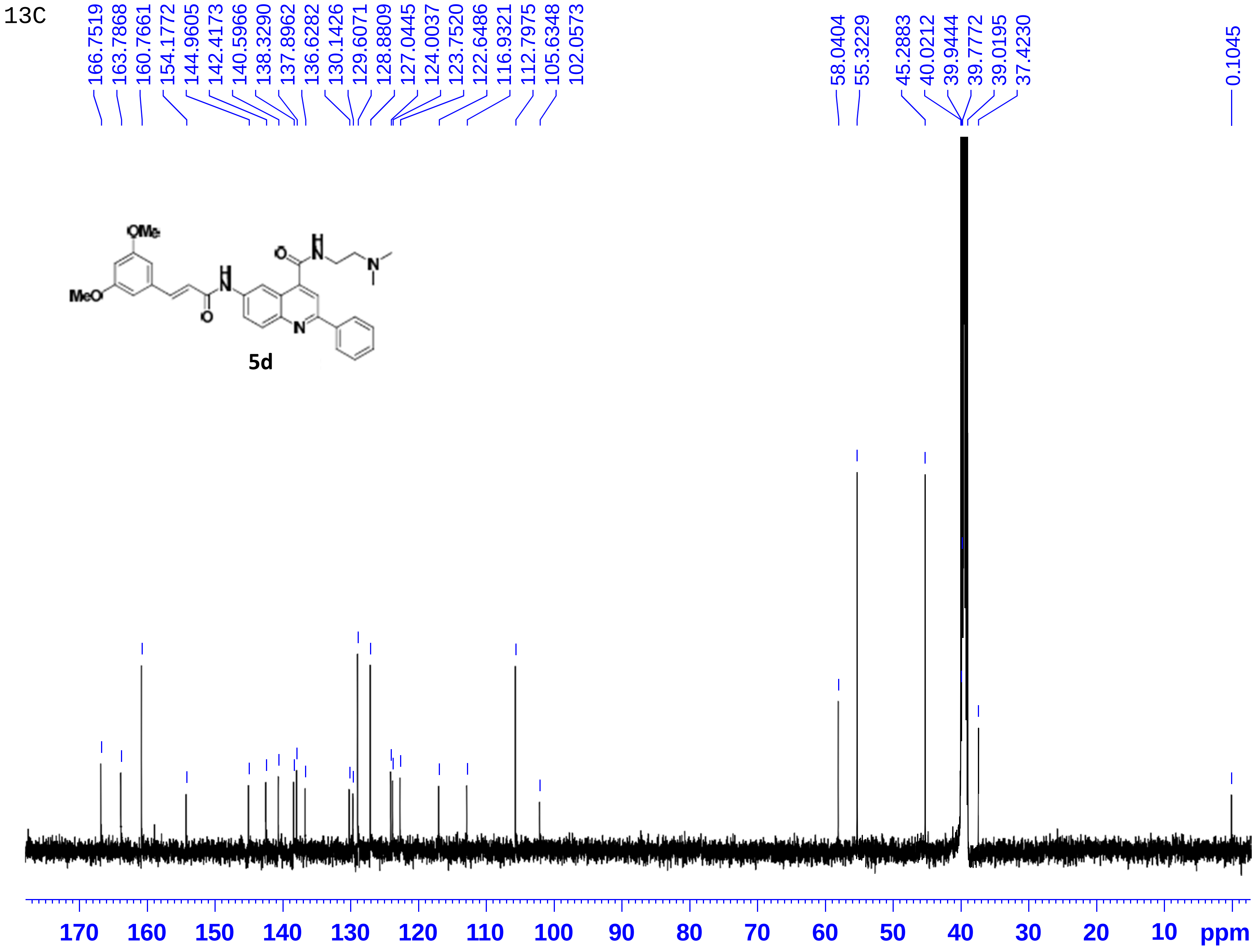

5d

Current Data Parameters

NAME	RK-III-30-20-13C
EXPNO	1
PROCNO	1

F2 - Acquisition Parameters

Date_	20120611
Time	15.54
INSTRUM	spect
PROBHD	5 mm CPTXI 1H-
PULPROG	zgpg30
TD	65536
SOLVENT	DMSO
NS	613
DS	4
SWH	30303.031 Hz
FIDRES	0.462388 Hz
AQ	1.0813940 sec
RG	23170.5
DW	16.500 usec
DE	6.50 usec
TE	298.1 K
D1	2.00000000 sec
D11	0.03000000 sec
TD0	1

======== CHANNEL f1 ========

NUC1	13C
P1	14.50 usec
PL1	-4.00 dB
PL1W	41.39080048 W
SFO1	125.7703638 MHz

======== CHANNEL f2 ========

CPDPRG2	waltz16
NUC2	1H
PCPD2	80.00 usec
PL2	-3.50 dB
PL12	13.50 dB
PL13	8.00 dB
PL2W	4.10509157 W
PL12W	0.08190735 W
PL13W	0.29061824 W
SFO2	500.1320005 MHz

F2 - Processing parameters

SI	32768
SF	125.7578482 MHz
WDW	EM
SSB	0
LB	1.00 Hz
GB	0
PC	1.40

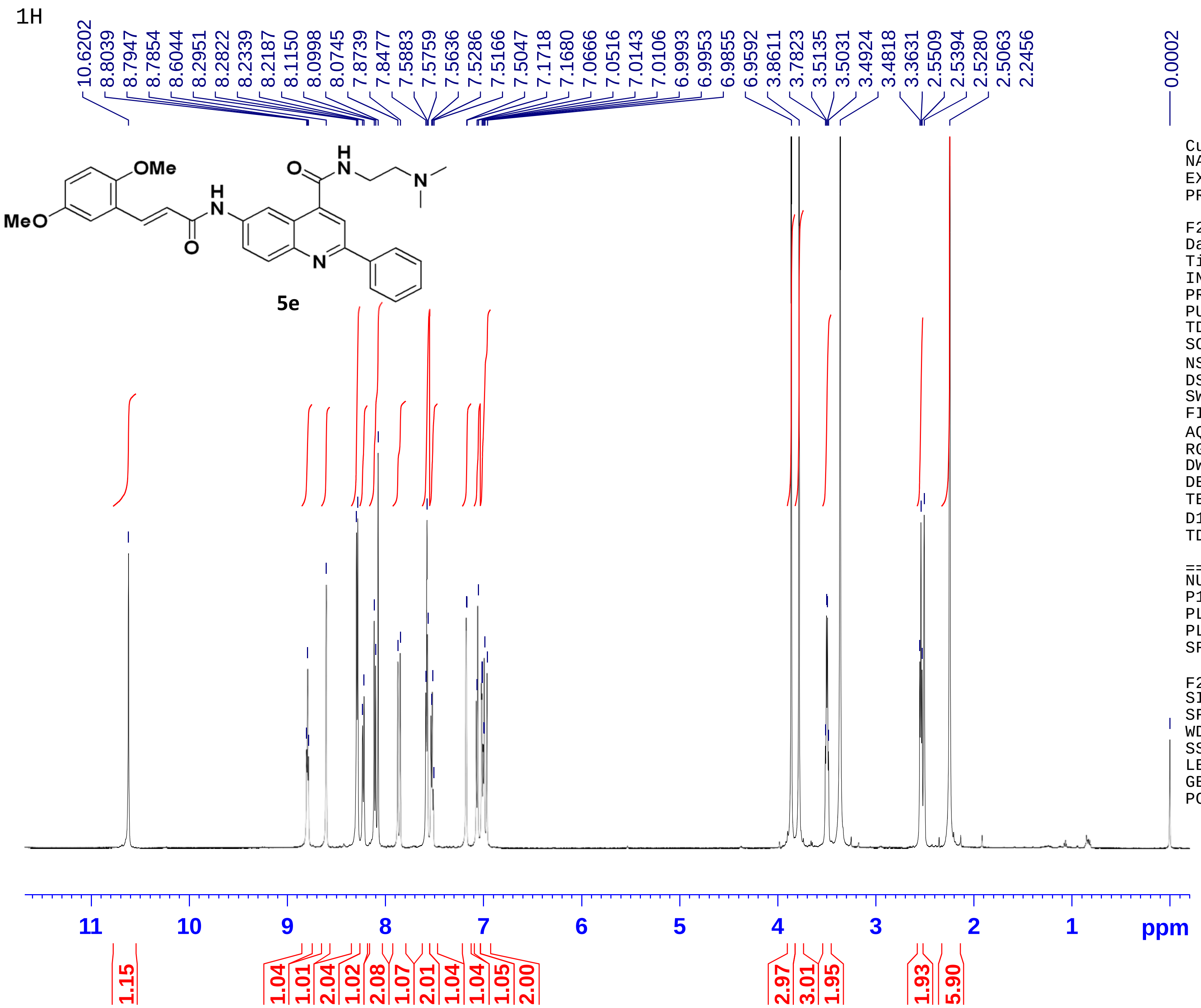


```
Current Data Parameters
NAME          RK-II-196-23
EXPNO                    1
PROCNO                   1

F2 - Acquisition Parameters
Date_             20120306
Time                 16.15
INSTRUM              spect
PROBHD   5 mm CPTCI 1H-
PULPROG                 zg
TD                   32768
SOLVENT               DMSO
NS                       1
DS                       0
SWH               8389.262 Hz
FIDRES            0.256020 Hz
AQ               1.9530228 sec
RG                      18
DW                  59.600 usec
DE                    6.50 usec
TE                   298.0 K
D1              2.00000000 sec
TD0                      1

======== CHANNEL f1 ========
NUC1                    1H
P1                    9.25 usec
PL1                  -1.00 dB
PL1W            3.00416374 W
SFO1           600.1328170 MHz

F2 - Processing parameters
SI                   32768
SF             600.1300032 MHz
WDW                     no
SSB      0
LB       0 Hz
GB       0
PC                    1.00
```

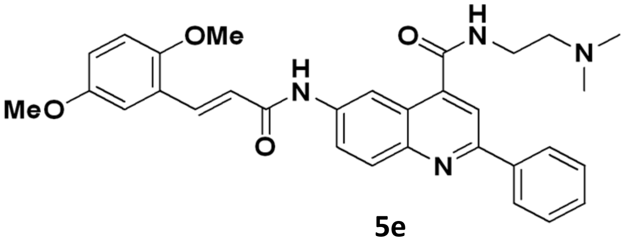

5e

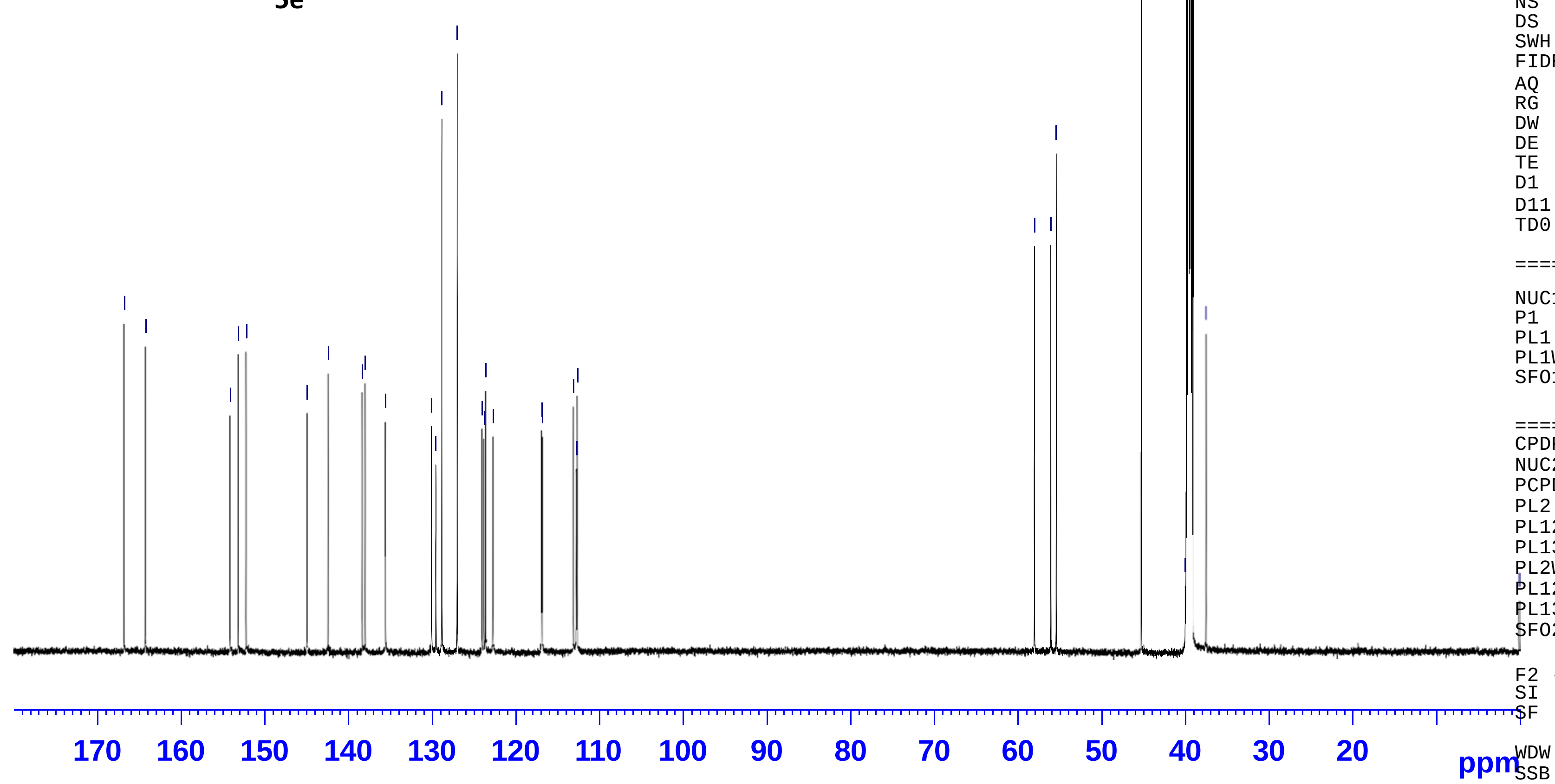


```
Current Data Parameters
NAME        RK-II-196-23-13C
EXPNO                      1
PROCNO                     1

F2 - Acquisition Parameters
Date_               20120306
Time                   16.17
INSTRUM                spect
PROBHD     5 mm CPTCI 1H-
PULPROG              zgpg30
TD                     65536
SOLVENT                 DMSO
NS                       861
DS                         4
SWH               36231.883 Hz
FIDRES             0.552855 Hz
AQ                0.9044468 sec
RG                    5160.6
DW                    13.800 usec
DE                    100.00 usec
TE                     298.0 K
D1                2.00000000 sec
D11               0.03000000 sec
TD0                        1

======== CHANNEL f1 ========
NUC1                     13C
P1                     15.00 usec
PL1                    -2.80 dB
PL1W             79.45259094 W
SFO1             150.9178993 MHz

======== CHANNEL f2 ========
CPDPRG2              waltz16
NUC2                      1H
PCPD2                  80.00 usec
PL2                    -5.40 dB
PL12                   10.70 dB
PL13                  120.00 dB
PL2W              8.27415466 W
PL12W             0.20310640 W
PL13W      0 W
SFO2             600.1324005 MHz

F2 - Processing parameters
SI                     32768
SF               150.9028823 MHz
WDW        0              EM
SSB
LB         0
GB                      1.00 Hz
PC
                        1.40
```

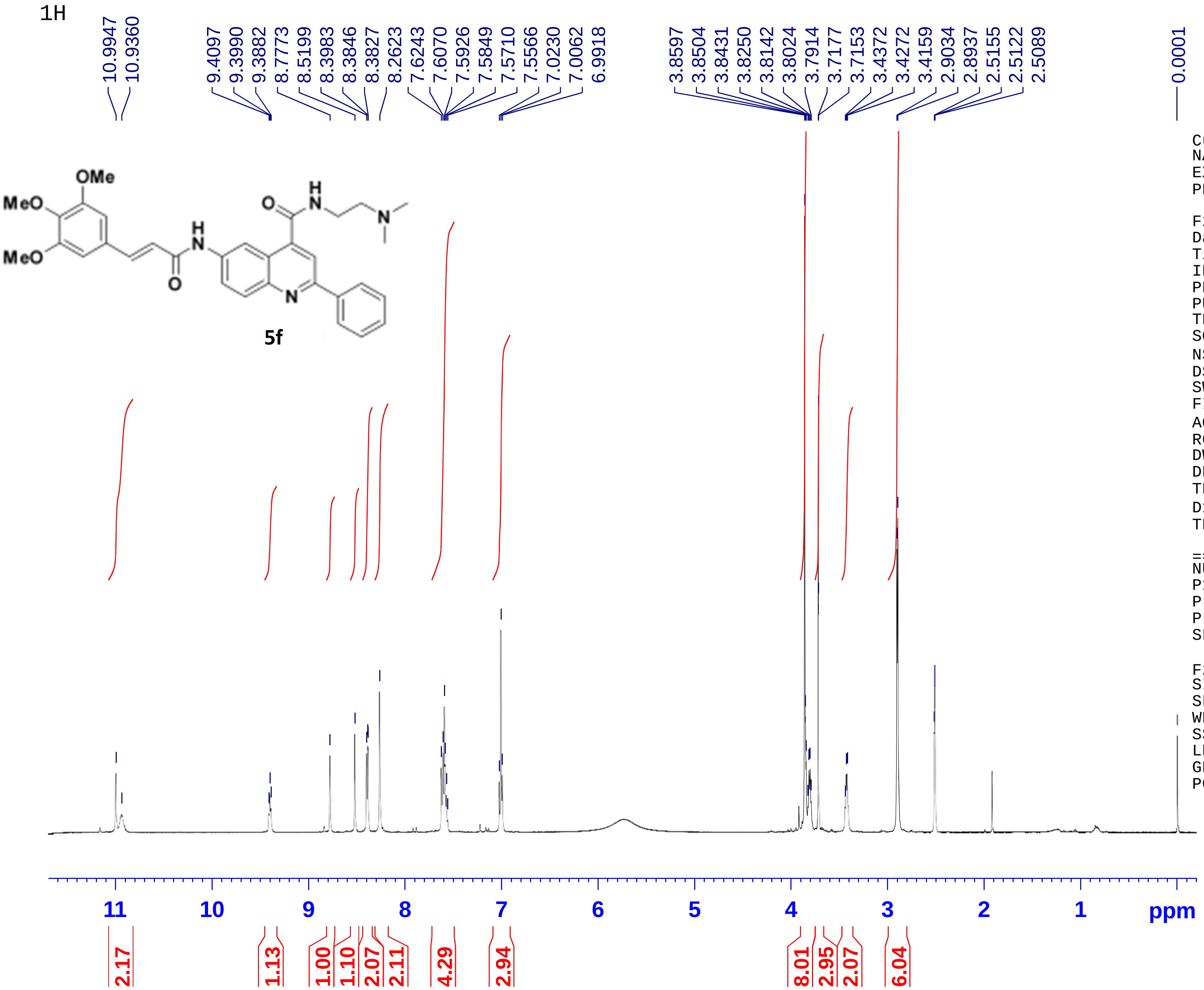

BRUKER

Current Data Parameters
NAME RK-II-164-28
EXPNO 1
PROCNO 1

F2 - Acquisition Parameters
Date_ 20110718
Time 15.53
INSTRUM spect
PROBHD 5 mm CPQNP 1H/
PULPROG zg
TD 32768
SOLVENT DMSO
NS 1
DS 0
SWH 7002.801 Hz
FIDRES 0.213709 Hz
AQ 2.3396852 sec
RG 18
DW 71.400 usec
DE 6.50 usec
TE 292.9 K
D1 2.00000000 sec
TD0 1

======== CHANNEL f1 ========
NUC1 1H
P1 9.25 usec
PL1 -1.00 dB
PL1W 2.30846262 W
SFO1 500.1323476 MHz

F2 - Processing parameters
SI 32768
SF 500.1299994 MHz
WDW no
SSB 0
LB 0 Hz
GB 0
PC 1.00

13C

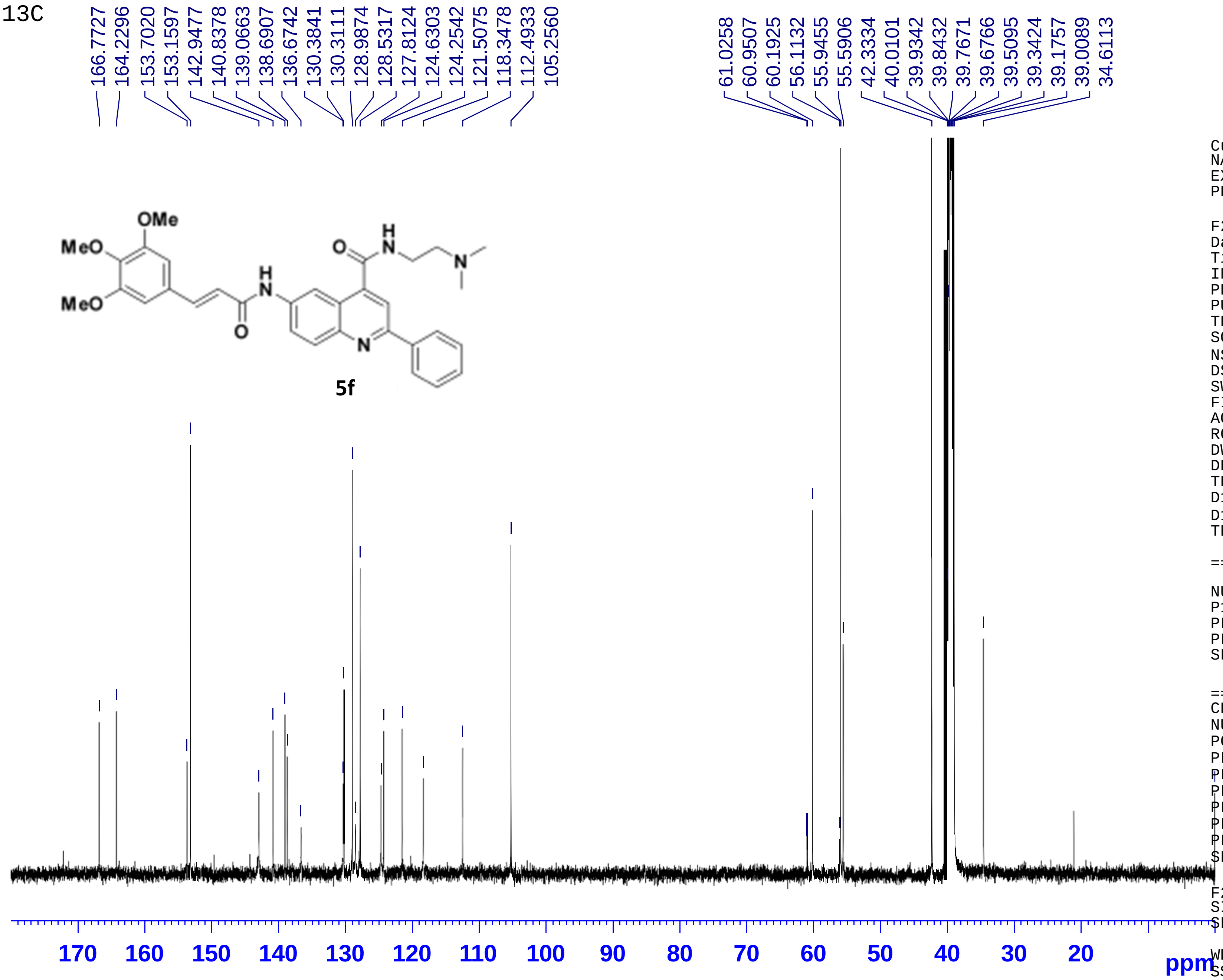

5f

Current Data Parameters
NAME RK-II-164-28-13C
EXPNO 2
PROCNO 1

F2 - Acquisition Parameters
Date_ 20110718
Time 15.56
INSTRUM spect
PROBHD 5 mm CPQNP 1H/
PULPROG zgpg30
TD 65536
SOLVENT DMSO
NS 208
DS 4
SWH 30303.031 Hz
FIDRES 0.462388 Hz
AQ 1.0813940 sec
RG 4096
DW 16.500 usec
DE 6.50 usec
TE 293.0 K
D1 2.00000000 sec
D11 0.03000000 sec
TD0 1

======== CHANNEL f1 ========

NUC1 13C
P1 10.10 usec
PL1 -4.00 dB
PL1W 41.39080048 W
SFO1 125.7703638 MHz

======== CHANNEL f2 ========
CPDPRG2 waltz16
NUC2 1H
PCPD2 80.00 usec
PL2 -5.00 dB
PL12 9.50 dB
PL13 12.50 dB
PL2W 5.79859591 W
PL12W 0.20574196 W
PL13W 0.10311524 W
SFO2 500.1320005 MHz

F2 - Processing parameters
SI 32768
SF 125.7578420 MHz
WDW 0 EM
SSB
LB 0 1.00 Hz
GB
PC 1.40

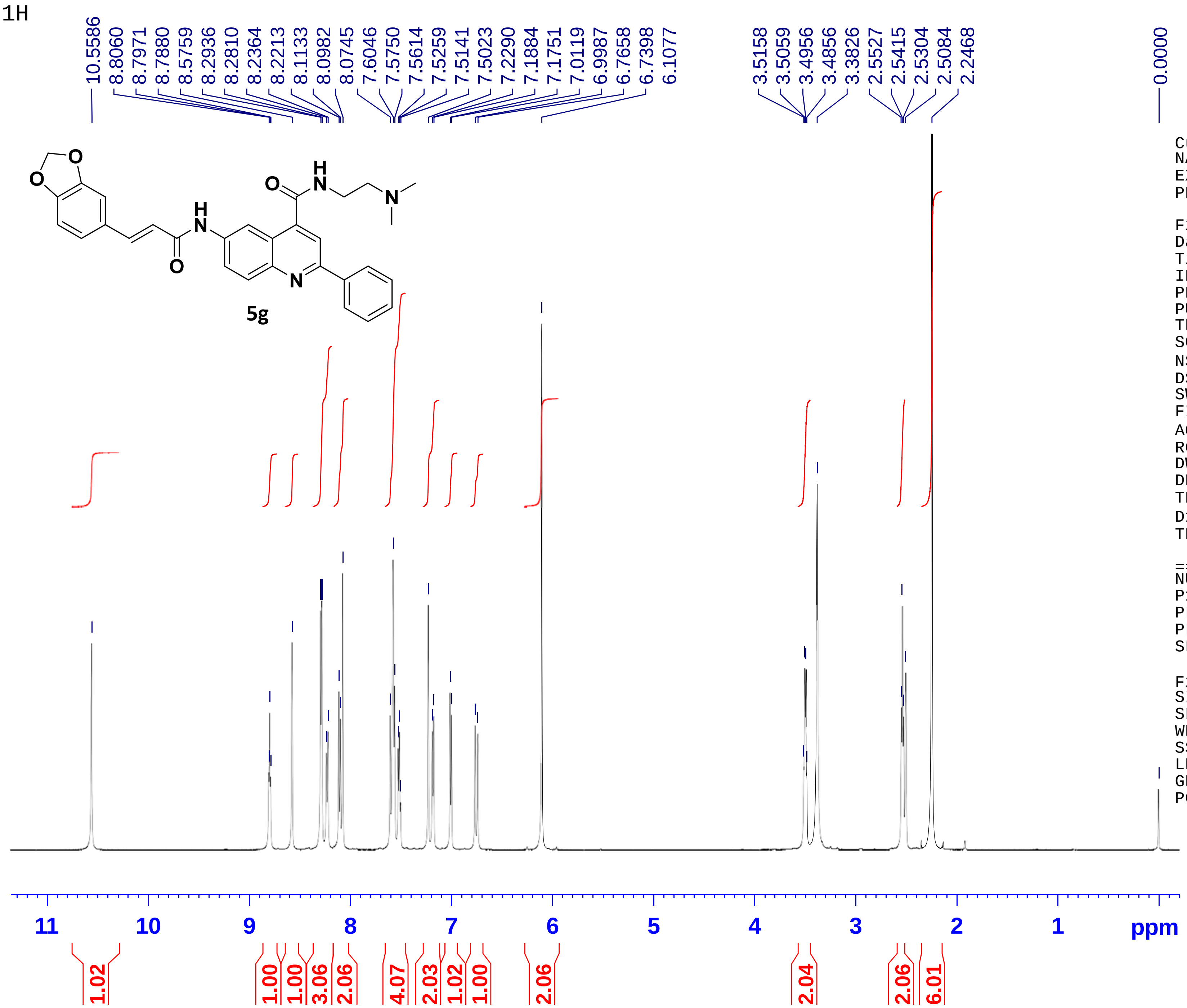

Current Data Parameters
NAME RK-II-197-25
EXPNO 1
PROCNO 1

F2 - Acquisition Parameters
Date_ 20120306
Time 17.03
INSTRUM spect
PROBHD 5 mm CPTCI 1H-
PULPROG zg
TD 32768
SOLVENT DMSO
NS 1
DS 0
SWH 8389.262 Hz
FIDRES 0.256020 Hz
AQ 1.9530228 sec
RG 18
DW 59.600 usec
DE 6.50 usec
TE 300.0 K
D1 2.00000000 sec
TD0 1

======== CHANNEL f1 ========
NUC1 1H
P1 9.25 usec
PL1 -1.00 dB
PL1W 3.00416374 W
SFO1 600.1328170 MHz

F2 - Processing parameters
SI 32768
SF 600.1300021 MHz
WDW no
SSB 0
LB 0 Hz
GB 0
PC 1.00

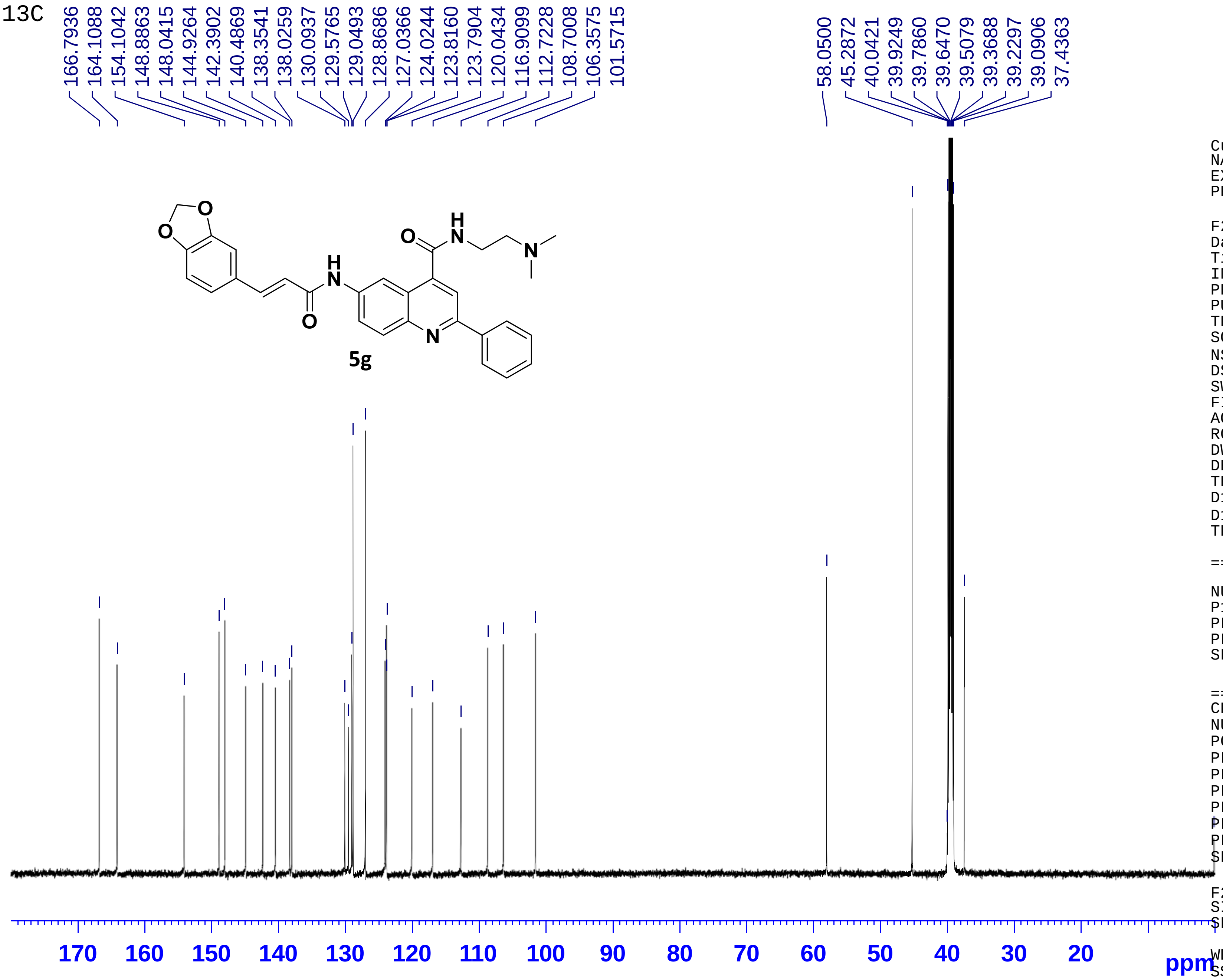


```
Current Data Parameters
NAME        RK-II-197-25-13C
EXPNO                      1
PROCNO                     1

F2 - Acquisition Parameters
Date_               20120306
Time                   17.05
INSTRUM                spect
PROBHD     5 mm CPTCI 1H-
PULPROG              zgpg30
TD                     65536
SOLVENT                 DMSO
NS                       310
DS                         4
SWH            36231.883 Hz
FIDRES          0.552855 Hz
AQ             0.9044468 sec
RG                      6502
DW                13.800 usec
DE               100.00 usec
TE                   298.0 K
D1            2.00000000 sec
D11           0.03000000 sec
TD0                        1

======== CHANNEL f1 ========
NUC1                     13C
P1                15.00 usec
PL1                 -2.80 dB
PL1W           79.45259094 W
SFO1         150.9178993 MHz

======== CHANNEL f2 ========
CPDPRG2              waltz16
NUC2                      1H
PCPD2             80.00 usec
PL2                 -5.40 dB
PL12                10.70 dB
PL13               120.00 dB
PL2W            8.27415466 W
PL12W           0.20310640 W
PL13W        0 W
SFO2         600.1324005 MHz

F2 - Processing parameters
SI                     32768
SF           150.9028807 MHz
WDW                       EM
SSB                        0
LB                   1.00 Hz
GB                         0
PC                      1.40
```

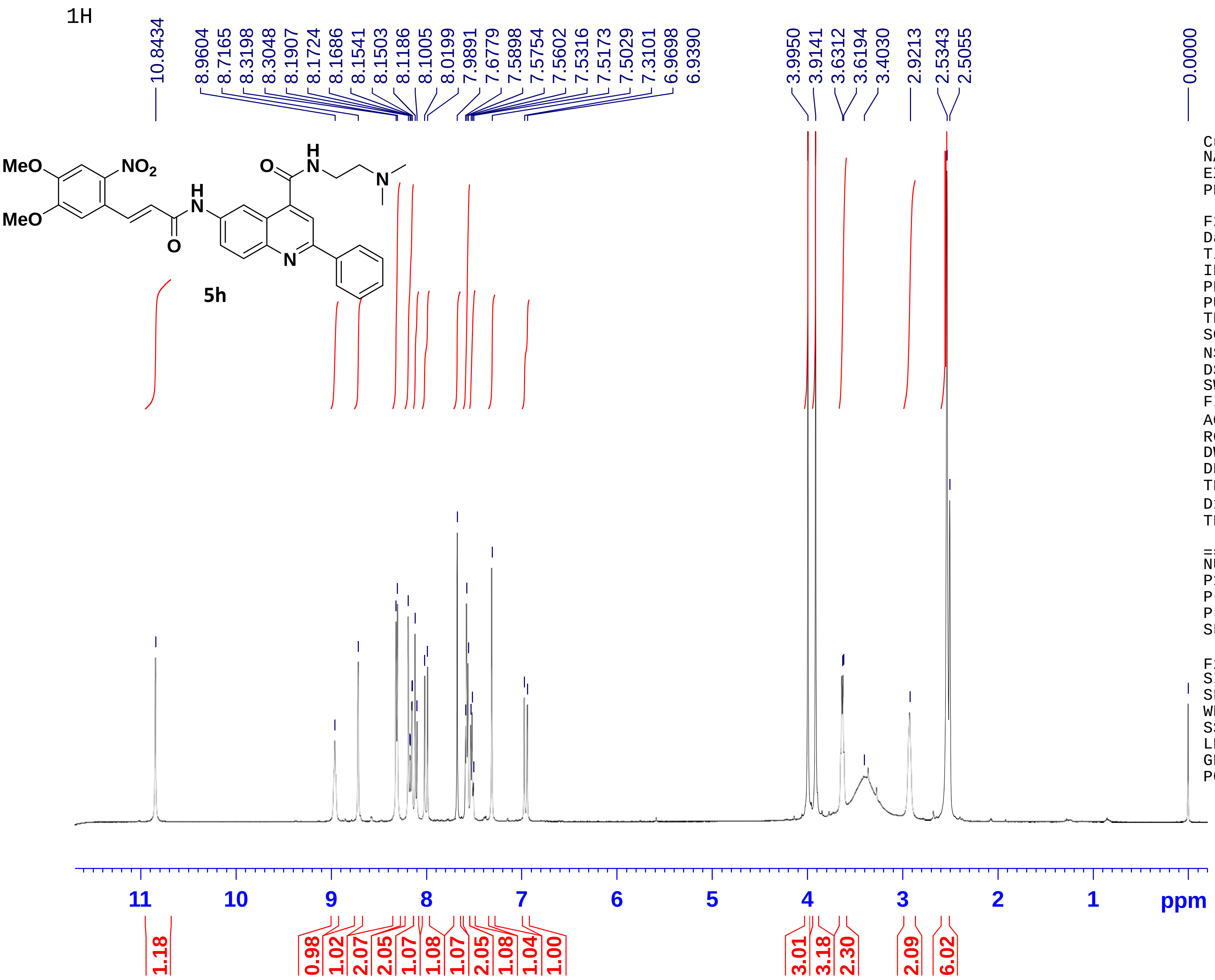

5h

BRUKER

Current Data Parameters
NAME RK-II-194-27
EXPNO 1
PROCNO 1

F2 - Acquisition Parameters
Date_ 20120220
Time 16.54
INSTRUM spect
PROBHD 5 mm CPTXI 1H-
PULPROG zg
TD 32768
SOLVENT DMSO
NS 1
DS 0
SWH 7002.801 Hz
FIDRES 0.213709 Hz
AQ 2.3396852 sec
RG 18
DW 71.400 usec
DE 6.50 usec
TE 302.0 K
D1 2.00000000 sec
TD0 1

======== CHANNEL f1 ========
NUC1 1H
P1 9.25 usec
PL1 -1.00 dB
PL1W 2.30846262 W
SFO1 500.1323476 MHz

F2 - Processing parameters
SI 32768
SF 500.1300020 MHz
WDW no
SSB 0
LB 0 Hz
GB 0
PC 1.00

13C

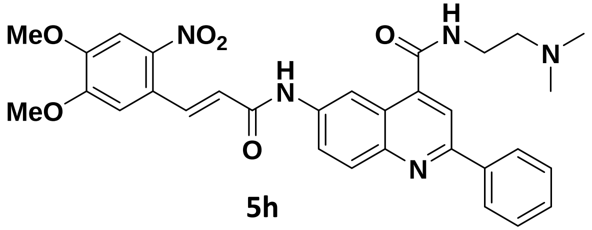

5h

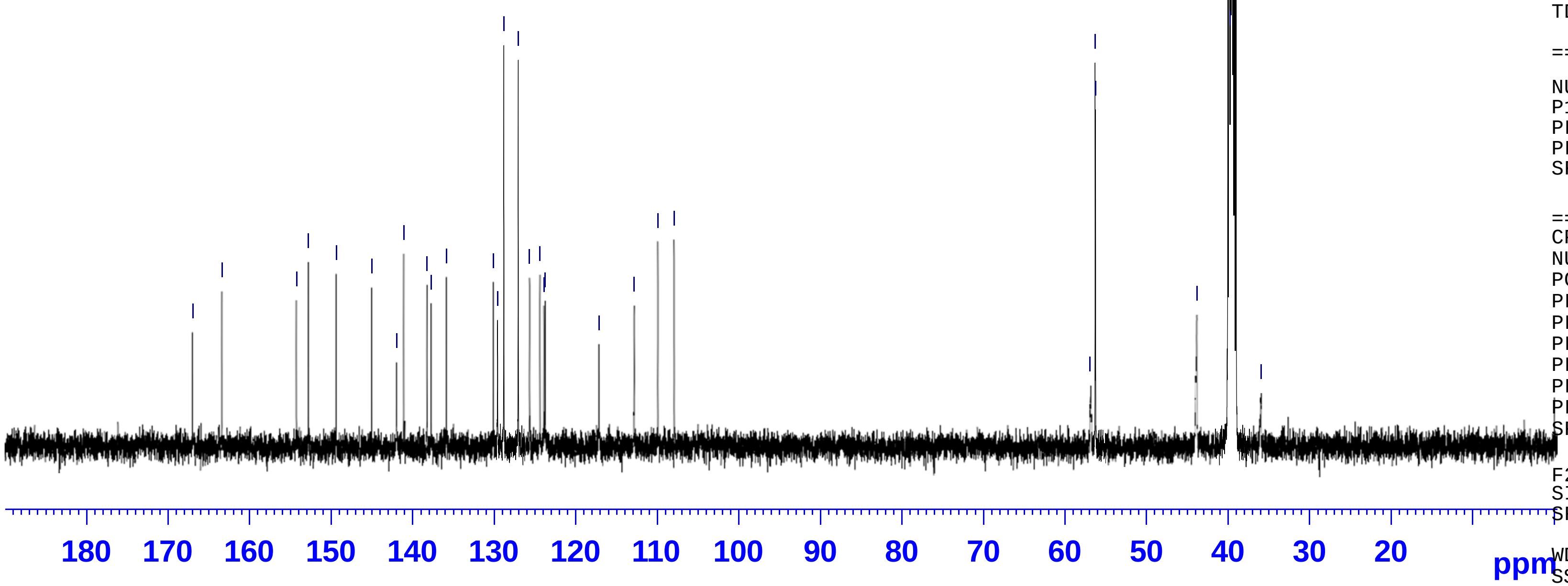

Current Data Parameters
NAME RK-II-194-27-13C
EXPNO 1
PROCNO 1

F2 - Acquisition Parameters
Date_ 20120220
Time 16.56
INSTRUM spect
PROBHD 5 mm CPTXI 1H-
PULPROG zgpg30
TD 65536
SOLVENT DMSO
NS 816
DS 4
SWH 30303.031 Hz
FIDRES 0.462388 Hz
AQ 1.0813940 sec
RG 23170.5
DW 16.500 usec
DE 6.50 usec
TE 302.0 K
D1 2.00000000 sec
D11 0.03000000 sec
TD0 1

======== CHANNEL f1 ========
NUC1 13C
P1 14.50 usec
PL1 -4.00 dB
PL1W 41.39080048 W
SFO1 125.7703638 MHz

======== CHANNEL f2 ========
CPDPRG2 waltz16
NUC2 1H
PCPD2 80.00 usec
PL2 -3.50 dB
PL12 13.50 dB
PL13 8.00 dB
PL2W 4.10509157 W
PL12W 0.08190735 W
PL13W 0.29061824 W
SFO2 500.1320005 MHz

F2 - Processing parameters
SI 32768
SF 125.7578535 MHz
WDW EM
SSB 0
LB 1.00 Hz
GB 0
PC 1.40

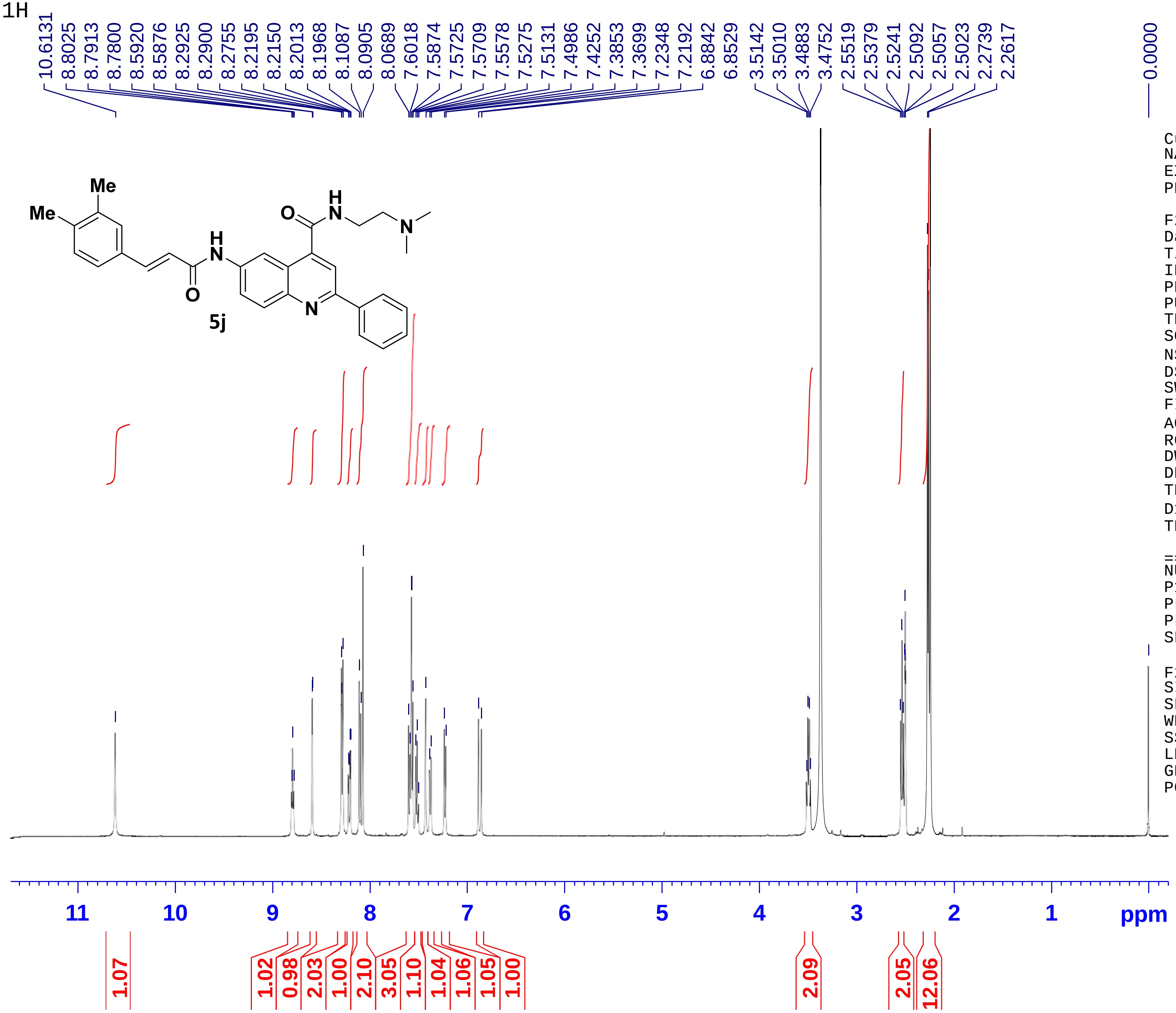


```
Current Data Parameters
NAME              RK-II-193
EXPNO                     1
PROCNO                    1

F2 - Acquisition Parameters
Date_              20120220
Time                  15.24
INSTRUM               spect
PROBHD   5 mm CPTXI 1H-
PULPROG                  zg
TD                    32768
SOLVENT                DMSO
NS                        1
DS                        0
SWH            7002.801 Hz
FIDRES         0.213709 Hz
AQ            2.3396852 sec
RG                       18
DW               71.400 usec
DE                 6.50 usec
TE                298.2 K
D1           2.00000000 sec
TD0                       1

======== CHANNEL f1 ========
NUC1                     1H
P1                 9.25 usec
PL1               -1.00 dB
PL1W         2.30846262 W
SFO1         500.1323476 MHz

F2 - Processing parameters
SI                    32768
SF           500.1300018 MHz
WDW                      no
SSB      0
LB       0 Hz
GB       0
PC                     1.00
```

13C

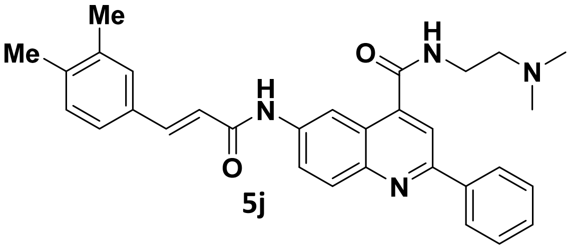

5j

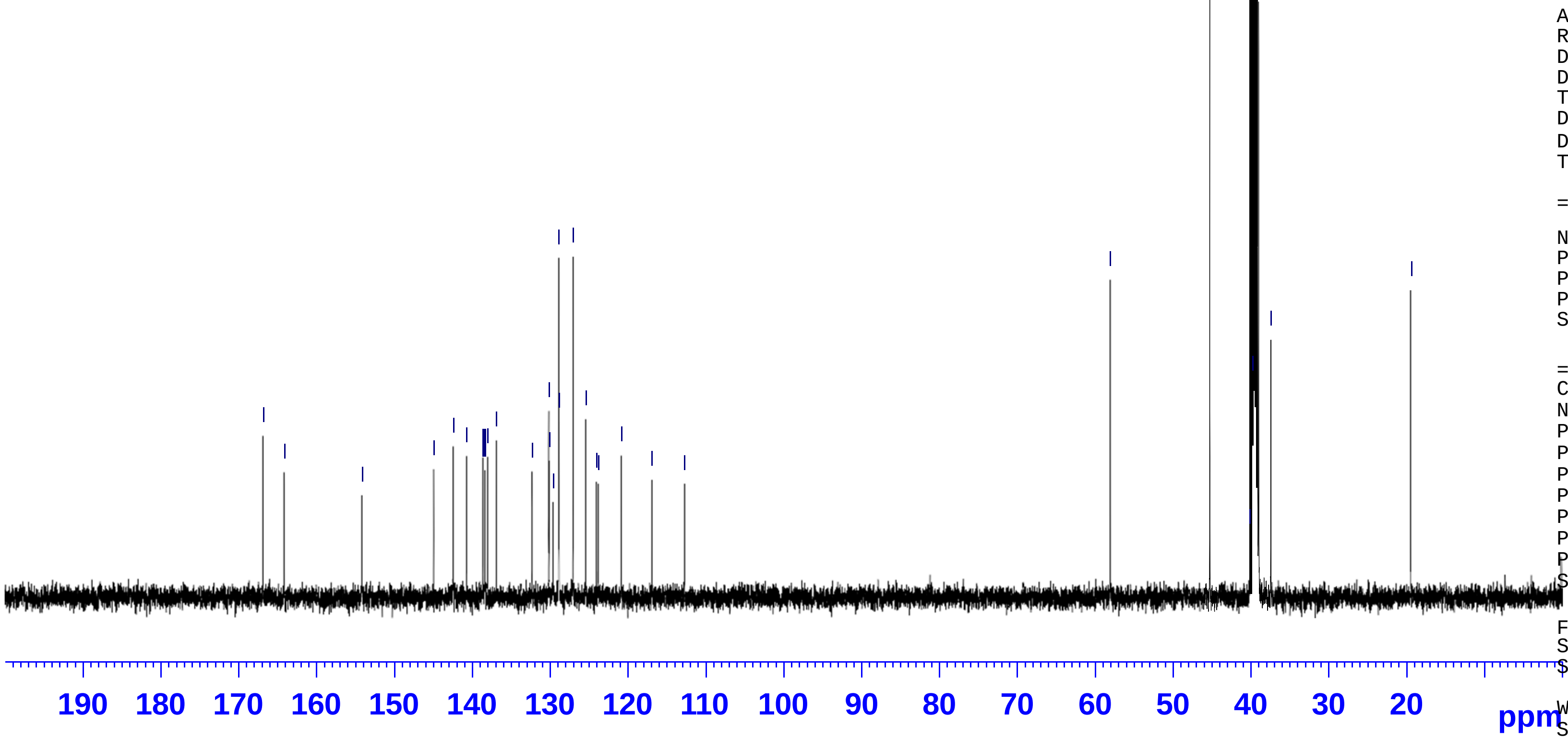

Current Data Parameters
NAME RK-II-193-13C
EXPNO 1
PROCNO 1

F2 - Acquisition Parameters
Date_ 20120220
Time 15.28
INSTRUM spect
PROBHD 5 mm CPTXI 1H-
PULPROG zgpg30
TD 65536
SOLVENT DMSO
NS 416
DS 4
SWH 30303.031 Hz
FIDRES 0.462388 Hz
AQ 1.0813940 sec
RG 23170.5
DW 16.500 usec
DE 6.50 usec
TE 298.0 K
D1 2.00000000 sec
D11 0.03000000 sec
TD0 1

======== CHANNEL f1 ========
NUC1 13C
P1 14.50 usec
PL1 -4.00 dB
PL1W 41.39080048 W
SFO1 125.7703638 MHz

======== CHANNEL f2 ========
CPDPRG2 waltz16
NUC2 1H
PCPD2 80.00 usec
PL2 -3.50 dB
PL12 13.50 dB
PL13 8.00 dB
PL2W 4.10509157 W
PL12W 0.08190735 W
PL13W 0.29061824 W
SFO2 500.1320005 MHz

F2 - Processing parameters
SI 32768
SF 125.7578488 MHz
WDW EM
SSB 0
LB 1.00 Hz
GB 0
PC 1.40

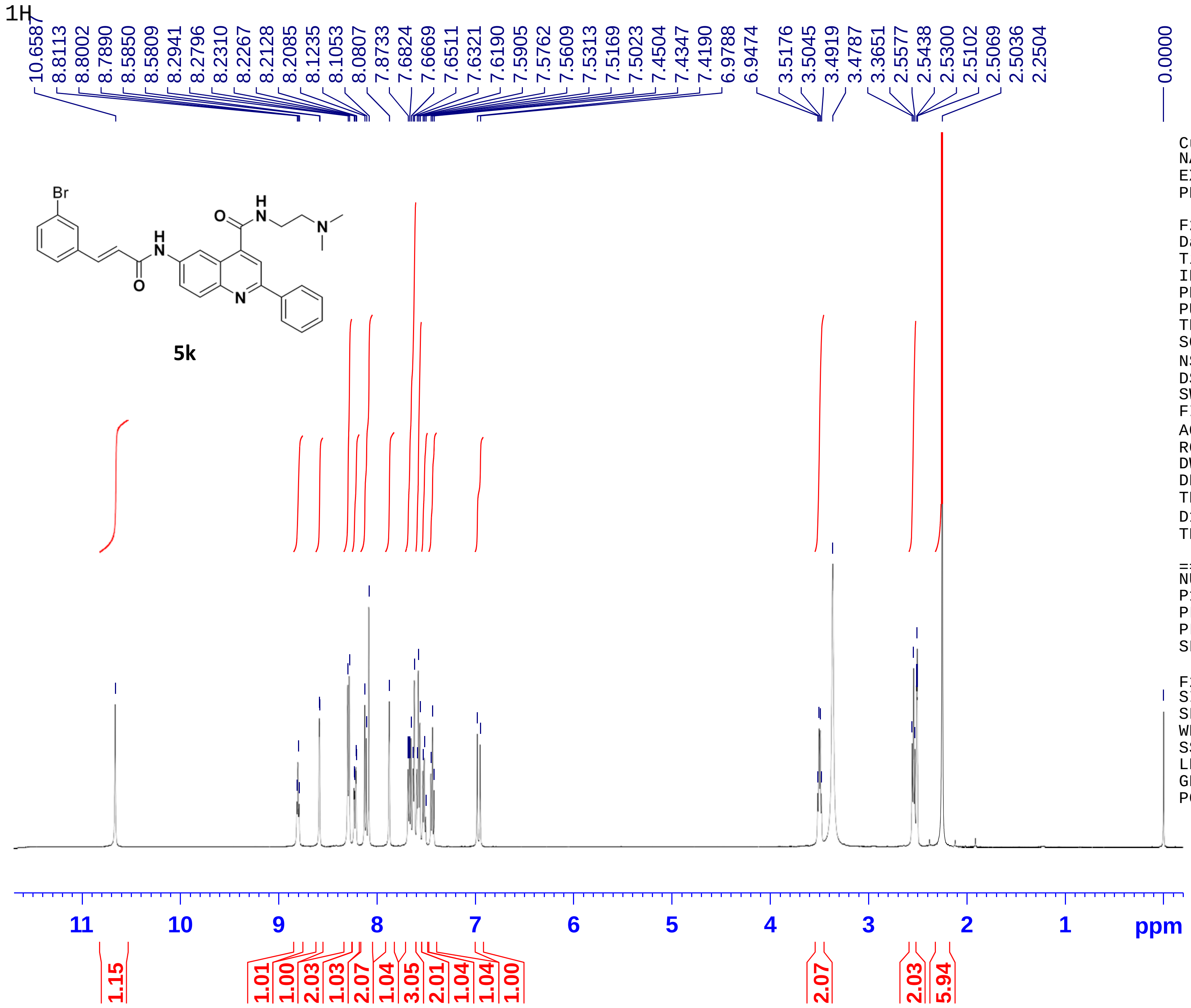

BRUKER

Current Data Parameters
NAME RK-II-195-24
EXPNO 1
PROCNO 1

F2 - Acquisition Parameters
Date_ 20120220
Time 16.22
INSTRUM spect
PROBHD 5 mm CPTXI 1H-
PULPROG zg
TD 32768
SOLVENT DMSO
NS 1
DS 0
SWH 7002.801 Hz
FIDRES 0.213709 Hz
AQ 2.3396852 sec
RG 18
DW 71.400 usec
DE 6.50 usec
TE 298.1 K
D1 2.00000000 sec
TD0 1

======== CHANNEL f1 ========
NUC1 1H
P1 9.25 usec
PL1 -1.00 dB
PL1W 2.30846262 W
SFO1 500.1323476 MHz

F2 - Processing parameters
SI 32768
SF 500.1300011 MHz
WDW no
SSB 0
LB 0 Hz
GB 0
PC 1.00

13C

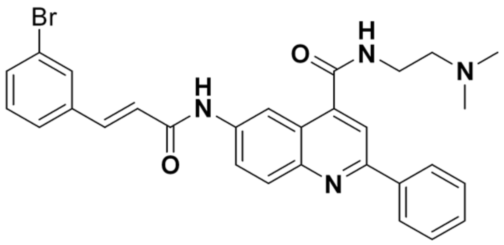

5k

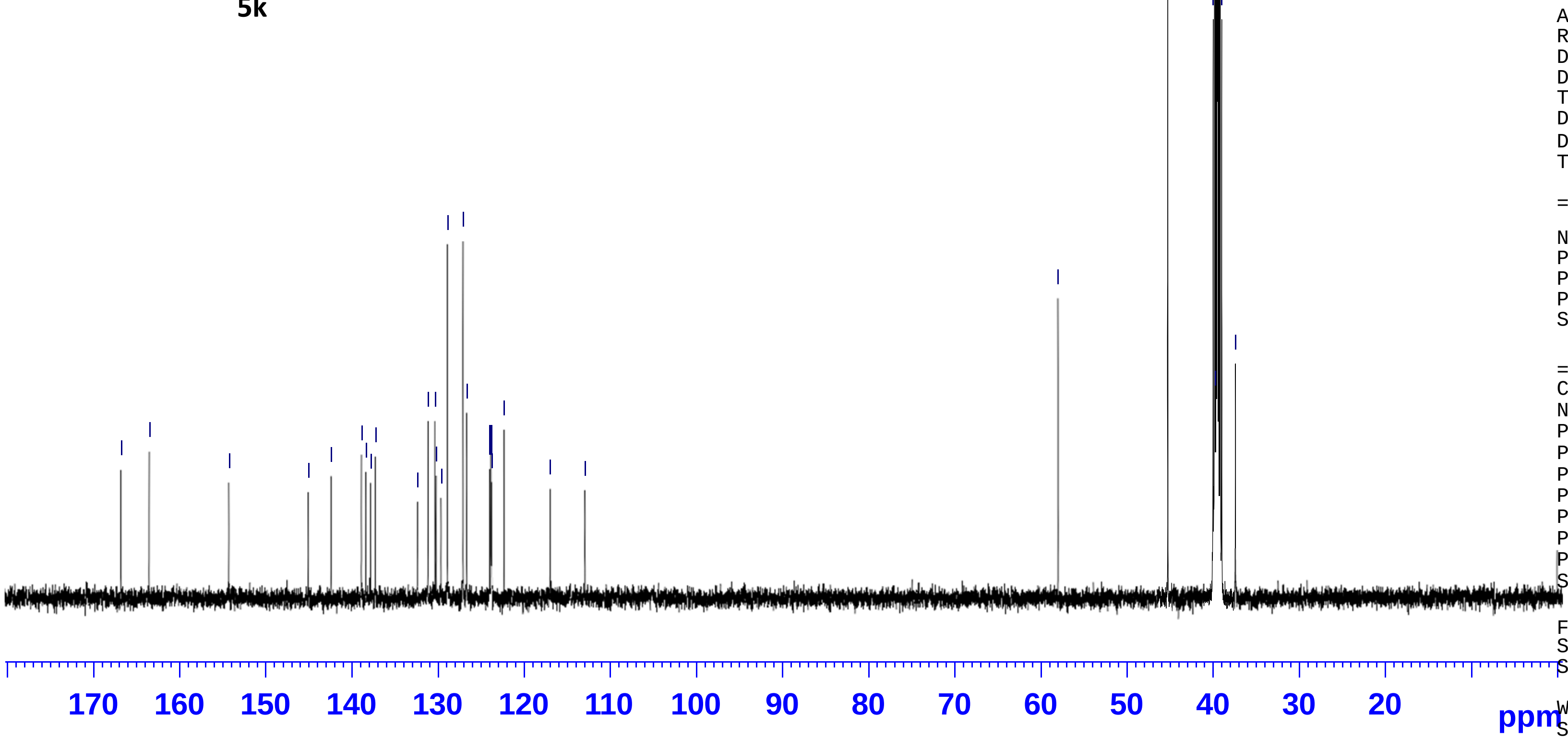


```
Current Data Parameters
NAME        RK-II-195-24-13C
EXPNO                      1
PROCNO                     1

F2 - Acquisition Parameters
Date_               20120220
Time                   16.25
INSTRUM                spect
PROBHD     5 mm CPTXI 1H-
PULPROG              zgpg30
TD                     65536
SOLVENT                 DMSO
NS                       416
DS                         4
SWH            30303.031 Hz
FIDRES          0.462388 Hz
AQ             1.0813940 sec
RG                   23170.5
DW               16.500 usec
DE                 6.50 usec
TE                  298.1 K
D1            2.00000000 sec
D11           0.03000000 sec
TD0                        1

======== CHANNEL f1 ========

NUC1                     13C
P1               14.50 usec
PL1                -4.00 dB
PL1W         41.39080048 W
SFO1       125.7703638 MHz

======== CHANNEL f2 ========
CPDPRG2              waltz16
NUC2                      1H
PCPD2            80.00 usec
PL2                -3.50 dB
PL12               13.50 dB
PL13                8.00 dB
PL2W          4.10509157 W
PL12W         0.08190735 W
PL13W         0.29061824 W
SFO2       500.1320005 MHz

F2 - Processing parameters
SI                     32768
SF         125.7578507 MHz
WDW          0            EM
SSB
LB           0       1.00 Hz
GB
PC
                        1.40
```

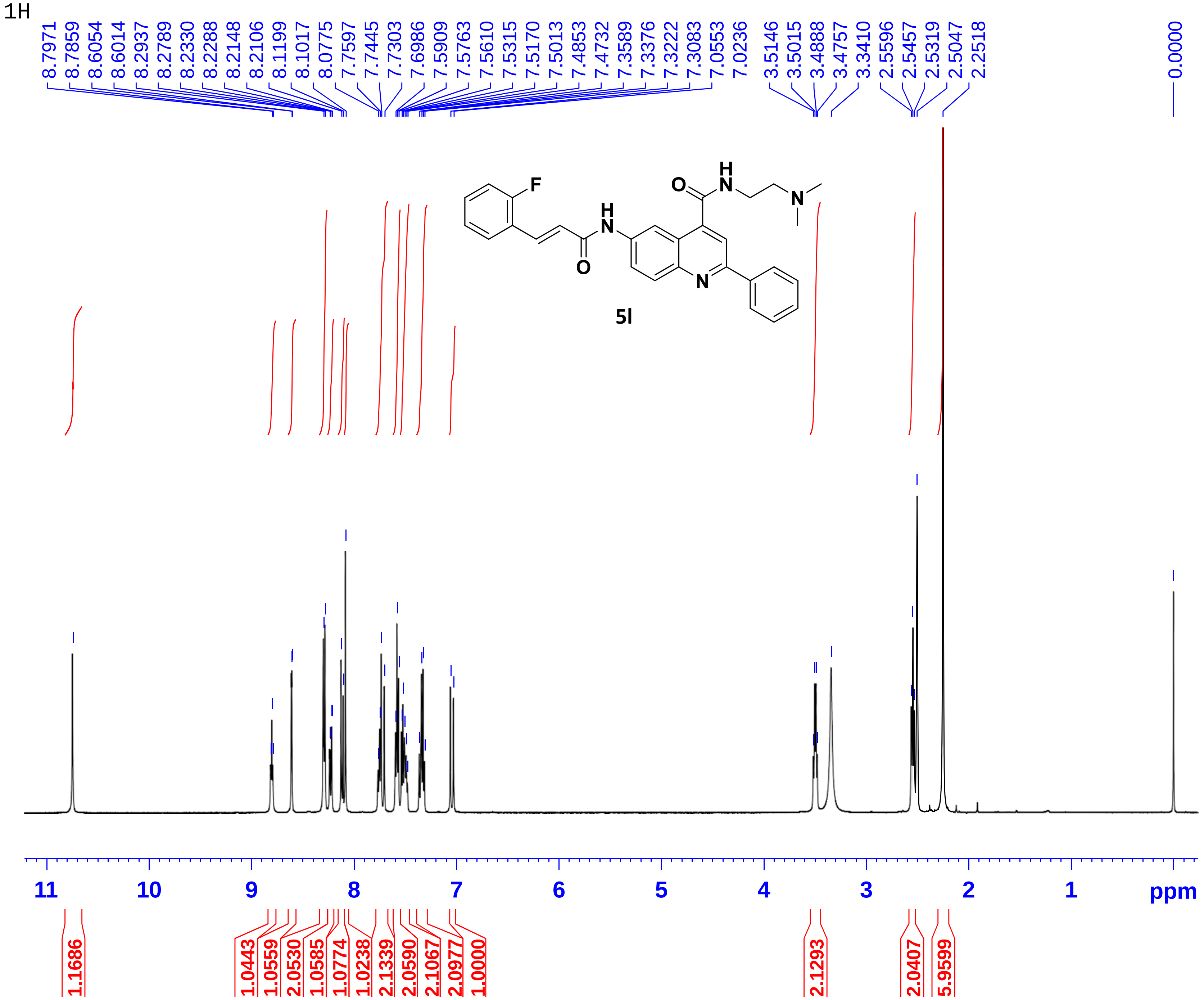

Current Data Parameters
NAME RK-III-43
EXPNO 1
PROCNO 1

F2 - Acquisition Parameters
Date_ 20120611
Time 15.06
INSTRUM spect
PROBHD 5 mm CPTXI 1H-
PULPROG zg
TD 32768
SOLVENT DMSO
NS 1
DS 0
SWH 7002.801 Hz
FIDRES 0.213709 Hz
AQ 2.3396852 sec
RG 18
DW 71.400 usec
DE 6.50 usec
TE 298.3 K
D1 2.00000000 sec
TD0 1

======== CHANNEL f1 ========
NUC1 1H
P1 9.25 usec
PL1 -1.00 dB
PL1W 2.30846262 W
SFO1 500.1323476 MHz

F2 - Processing parameters
SI 32768
SF 500.1300022 MHz
WDW no
SSB 0
LB 0 Hz
GB 0
PC 1.00

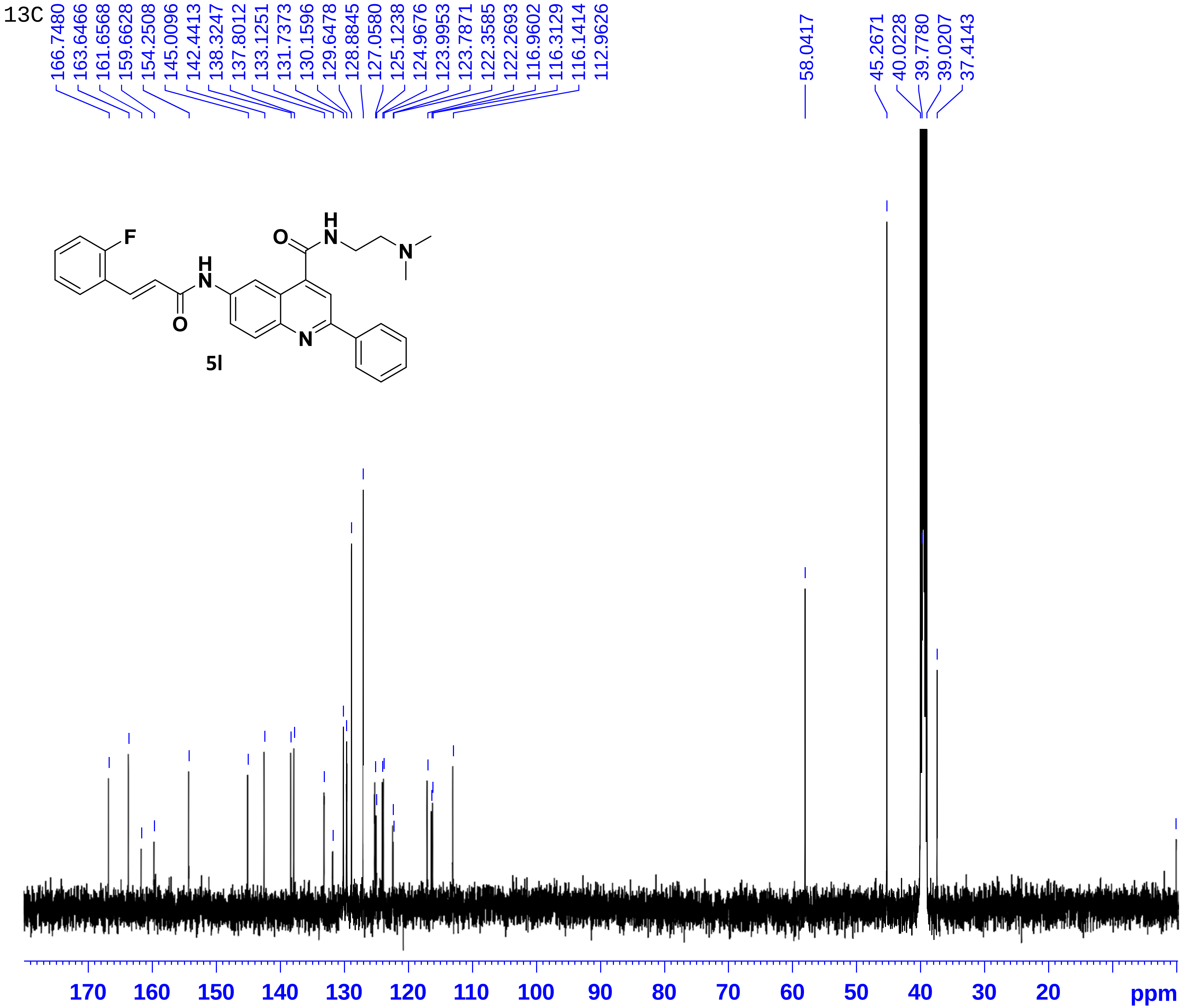


```
Current Data Parameters
NAME          RK-III-43-13C
EXPNO                     1
PROCNO                    1

F2 - Acquisition Parameters
Date_              20120611
Time                  15.09
INSTRUM               spect
PROBHD    5 mm CPTXI 1H-
PULPROG             zgpg30
TD                    65536
SOLVENT                DMSO
NS                      336
DS                        4
SWH              30303.031 Hz
FIDRES            0.462388 Hz
AQ               1.0813940 sec
RG                  23170.5
DW                   16.500 usec
DE                     6.50 usec
TE                    298.2 K
D1               2.00000000 sec
D11              0.03000000 sec
TD0                       1

======== CHANNEL f1 ========
NUC1                    13C
P1                    14.50 usec
PL1                   -4.00 dB
PL1W            41.39080048 W
SFO1            125.7703638 MHz

======== CHANNEL f2 ========
CPDPRG2             waltz16
NUC2                     1H
PCPD2                 80.00 usec
PL2                   -3.50 dB
PL12                  13.50 dB
PL13                   8.00 dB
PL2W             4.10509157 W
PL12W            0.08190735 W
PL13W            0.29061824 W
SFO2            500.1320005 MHz

F2 - Processing parameters
SI                    32768
SF              125.7578482 MHz
WDW                      EM
SSB       0
LB                     1.00 Hz
GB        0
PC                     1.40
```

1H

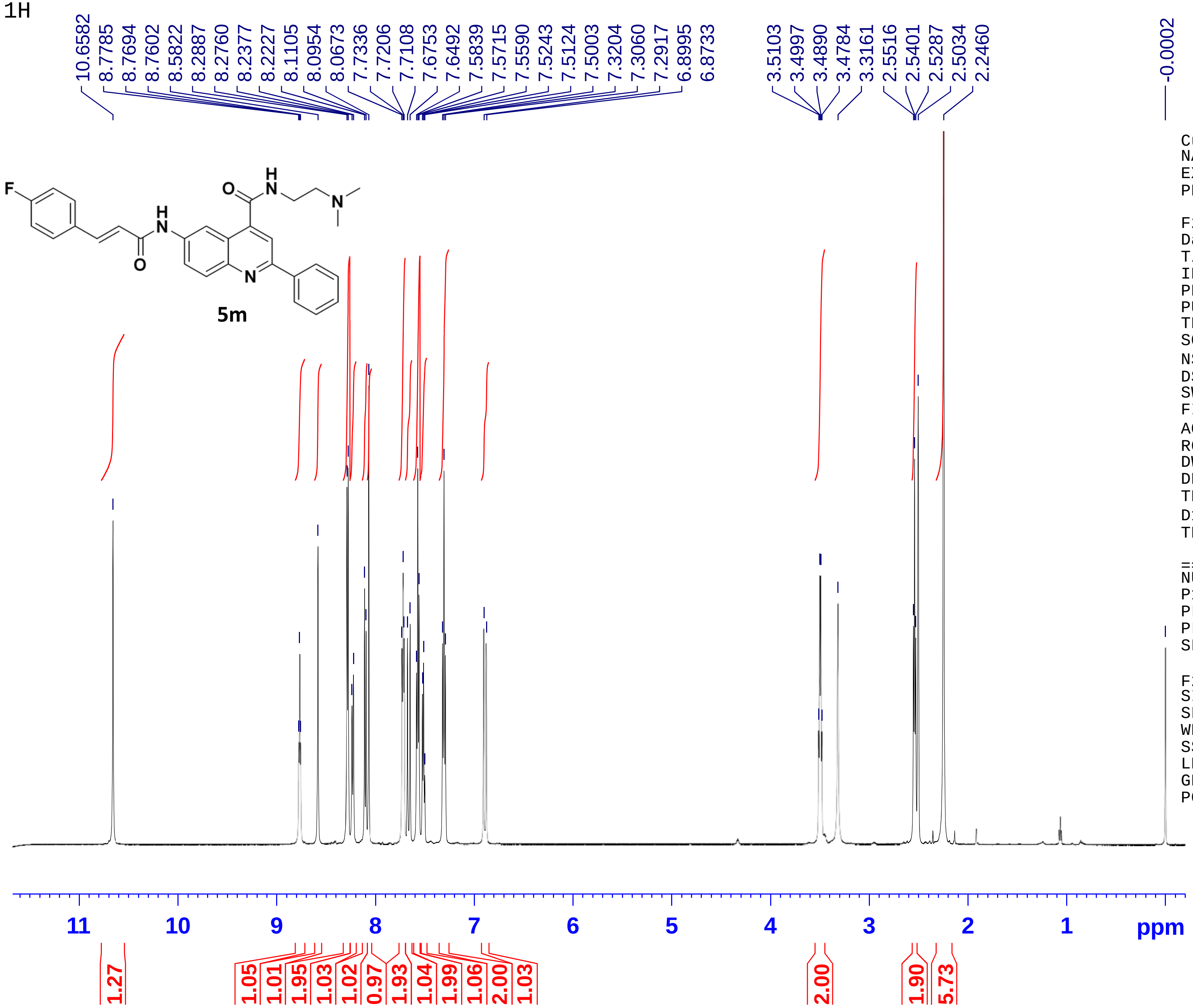


```
Current Data Parameters
NAME        RK-II-184-24-1H
EXPNO                     1
PROCNO                    1

F2 - Acquisition Parameters
Date_              20120306
Time                  14.12
INSTRUM               spect
PROBHD     5 mm CPTCI 1H-
PULPROG                  zg
TD                    32768
SOLVENT                DMSO
NS                        1
DS                        0
SWH                8389.262 Hz
FIDRES             0.256020 Hz
AQ                1.9530228 sec
RG                       18
DW                   59.600 usec
DE                     6.50 usec
TE                    305.4 K
D1               2.00000000 sec
TD0                       1

======== CHANNEL f1 ========
NUC1                     1H
P1                     9.25 usec
PL1                   -1.00 dB
PL1W             3.00416374 W
SFO1            600.1328170 MHz

F2 - Processing parameters
SI                    32768
SF              600.1300053 MHz
WDW                      no
SSB      0
LB       0 Hz
GB       0
PC                     1.00
```

13C

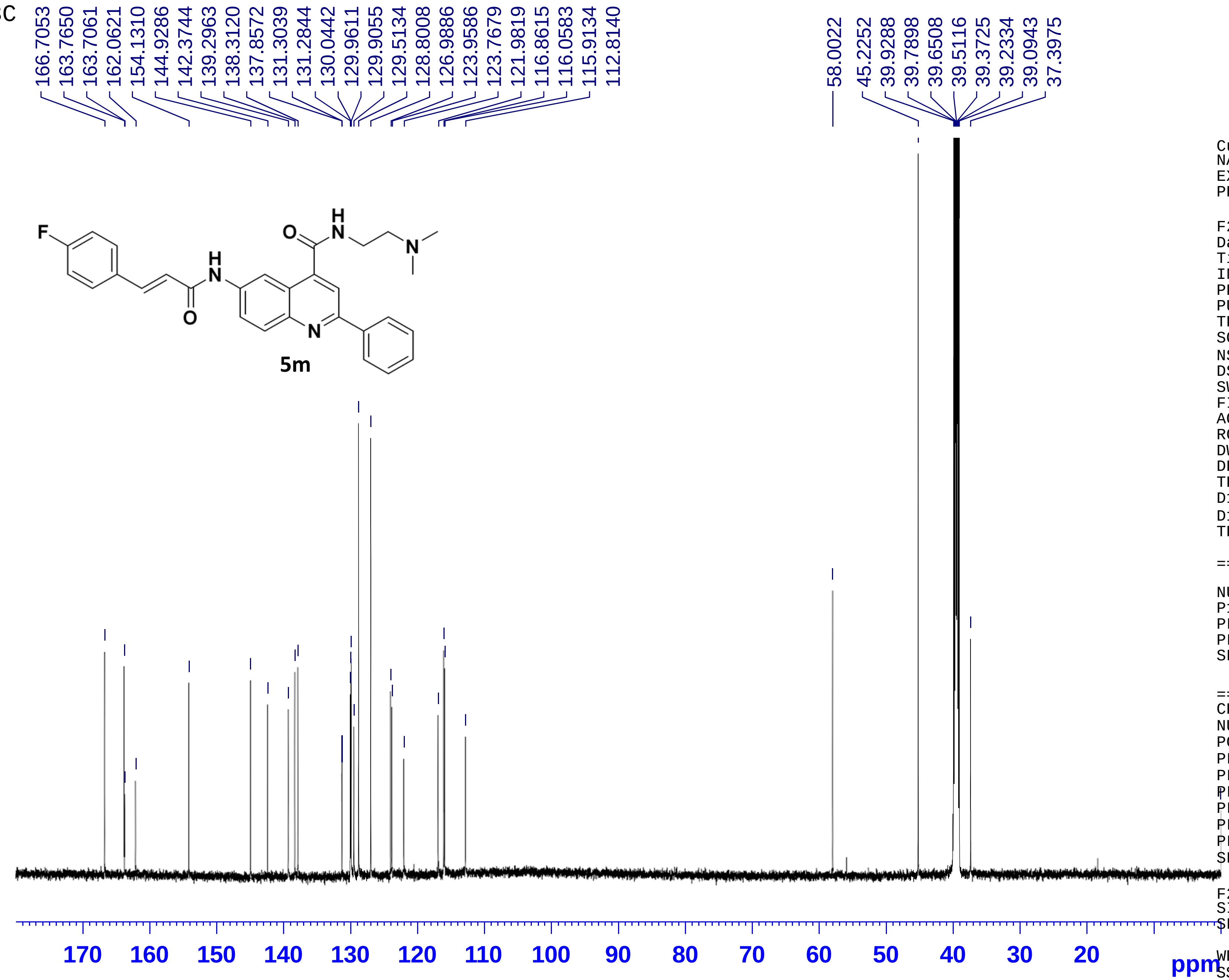

5m

Current Data Parameters
NAME RK-II-184-24-13C
EXPNO 1
PROCNO 1

F2 - Acquisition Parameters
Date_ 20120306
Time 14.15
INSTRUM spect
PROBHD 5 mm CPTCI 1H-
PULPROG zgpg30
TD 65536
SOLVENT DMSO
NS 336
DS 4
SWH 36231.883 Hz
FIDRES 0.552855 Hz
AQ 0.9044468 sec
RG 6502
DW 13.800 usec
DE 6.50 usec
TE 305.0 K
D1 2.00000000 sec
D11 0.03000000 sec
TD0 1

======== CHANNEL f1 ========
NUC1 13C
P1 15.00 usec
PL1 -2.80 dB
PL1W 79.45259094 W
SFO1 150.9178993 MHz

======== CHANNEL f2 ========
CPDPRG2 waltz16
NUC2 1H
PCPD2 80.00 usec
PL2 -5.40 dB
PL12 10.70 dB
PL13 120.00 dB
PL2W 8.27415466 W
PL12W 0.20310640 W
PL13W 0 W
SFO2 600.1324005 MHz

F2 - Processing parameters
SI 32768
SF 150.9028896 MHz
WDW EM
SSB 0
LB 1.00 Hz
GB 0
PC 1.40

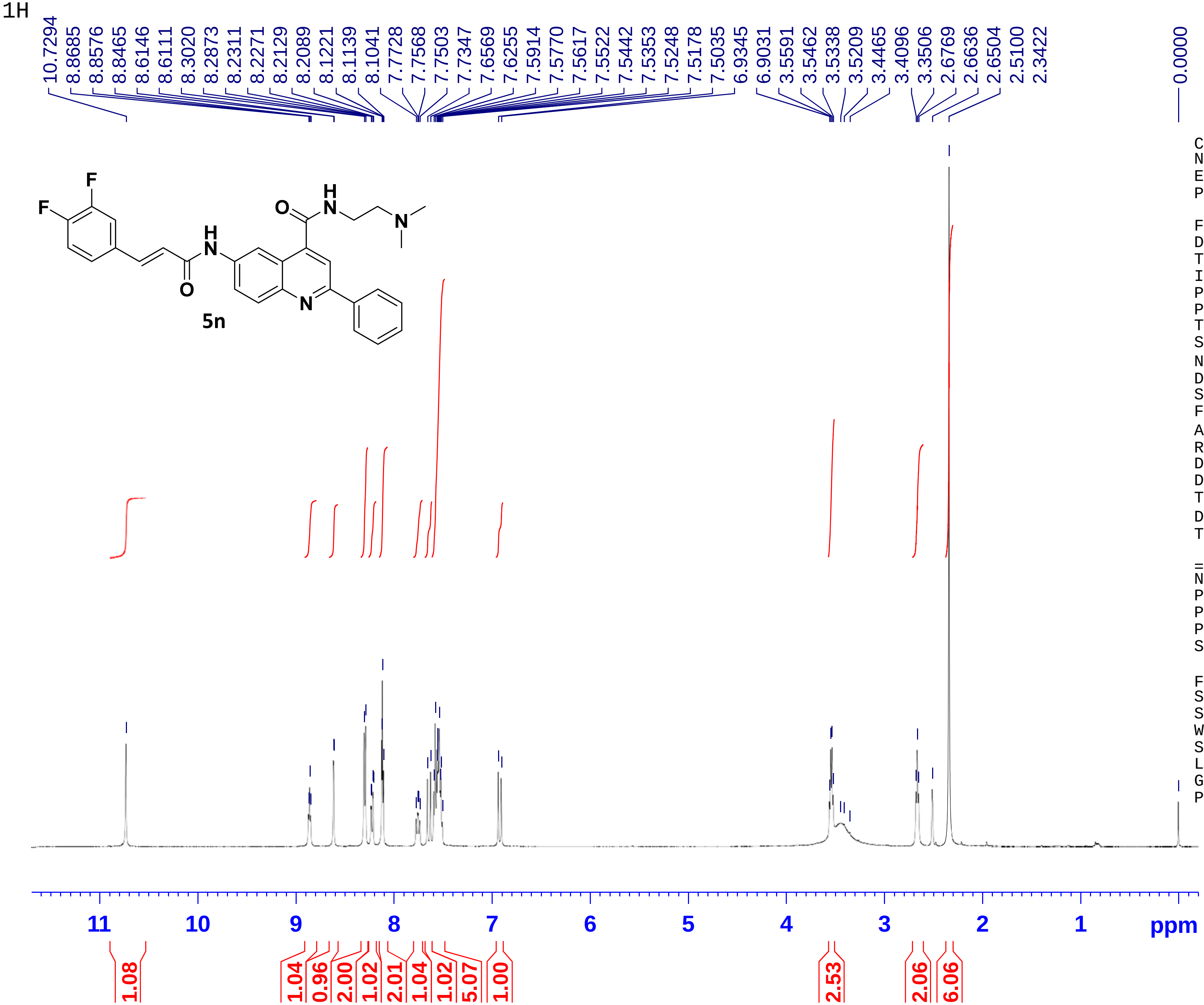

5n

BRUKER

```
Current Data Parameters
NAME              RK-II-192
EXPNO                     1
PROCNO                    1

F2 - Acquisition Parameters
Date_              20120220
Time                  14.43
INSTRUM               spect
PROBHD   5 mm CPTXI 1H-
PULPROG                  zg
TD                    32768
SOLVENT                DMSO
NS                        8
DS                        0
SWH            7002.801 Hz
FIDRES         0.213709 Hz
AQ            2.3396852 sec
RG                     14.3
DW              71.400 usec
DE                6.50 usec
TE                298.1 K
D1           2.00000000 sec
TD0                       1

======== CHANNEL f1 ========
NUC1                     1H
P1                9.25 usec
PL1               -1.00 dB
PL1W          2.30846262 W
SFO1        500.1323476 MHz

F2 - Processing parameters
SI                    32768
SF          500.1299996 MHz
WDW                      no
SSB         0
LB          0 Hz
GB          0
PC                     1.00
```

13C

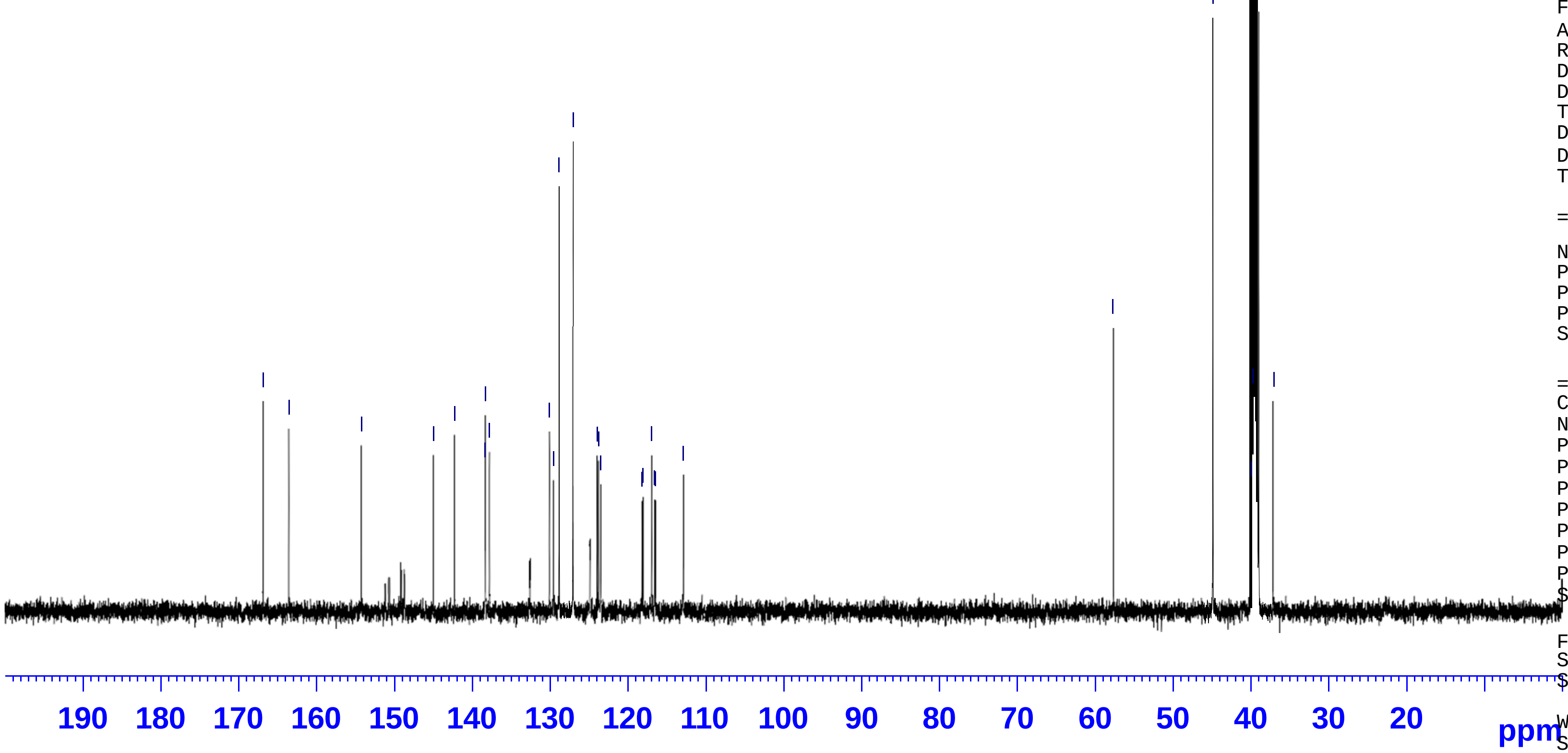

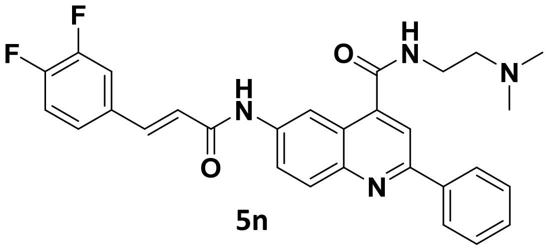

5n

Current Data Parameters
NAME RK-II-192-13C
EXPNO 2
PROCNO 1

F2 - Acquisition Parameters
Date_ 20120220
Time 14.49
INSTRUM spect
PROBHD 5 mm CPTXI 1H-
PULPROG zgpg30
TD 65536
SOLVENT DMSO
NS 496
DS 4
SWH 30303.031 Hz
FIDRES 0.462388 Hz
AQ 1.0813940 sec
RG 23170.5
DW 16.500 usec
DE 6.50 usec
TE 298.0 K
D1 2.00000000 sec
D11 0.03000000 sec
TD0 1

======== CHANNEL f1 ========
NUC1 13C
P1 14.50 usec
PL1 -4.00 dB
PL1W 41.39080048 W
SFO1 125.7703638 MHz

======== CHANNEL f2 ========
CPDPRG2 waltz16
NUC2 1H
PCPD2 80.00 usec
PL2 -3.50 dB
PL12 13.50 dB
PL13 8.00 dB
PL2W 4.10509157 W
PL12W 0.08190735 W
PL13W 0.29061824 W
SFO2 500.1320005 MHz

F2 - Processing parameters
SI 32768
SF 125.7578498 MHz
WDW EM
SSB 0
LB 1.00 Hz
GB 0
PC 1.40

1H

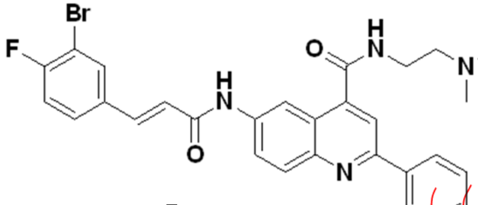

5o

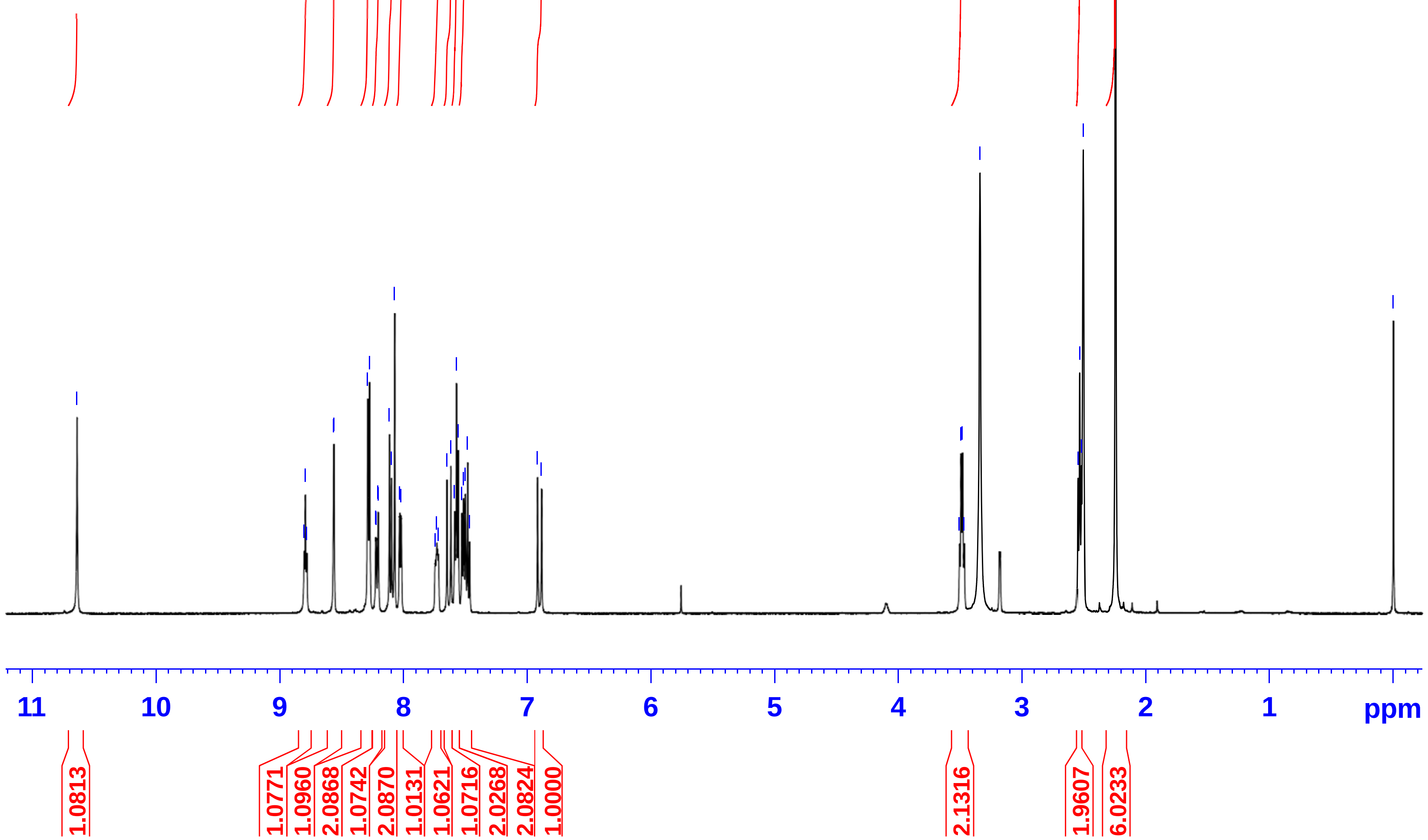

Current Data Parameters
NAME RK-III-31-26
EXPNO 1
PROCNO 1

F2 - Acquisition Parameters
Date_ 20120611
Time 16.29
INSTRUM spect
PROBHD 5 mm CPTXI 1H-
PULPROG zg
TD 32768
SOLVENT DMSO
NS 1
DS 0
SWH 7002.801 Hz
FIDRES 0.213709 Hz
AQ 2.3396852 sec
RG 18
DW 71.400 usec
DE 6.50 usec
TE 298.1 K
D1 2.00000000 sec
TD0 1

======== CHANNEL f1 ========
NUC1 1H
P1 9.25 usec
PL1 -1.00 dB
PL1W 2.30846262 W
SFO1 500.1323476 MHz

F2 - Processing parameters
SI 32768
SF 500.1300020 MHz
WDW no
SSB 0
LB 0 Hz
GB 0
PC 1.00

13C

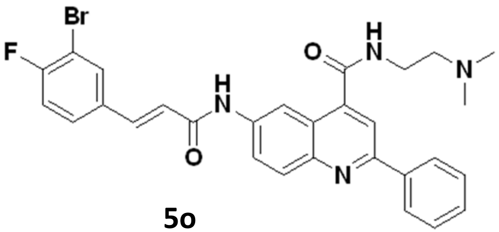

5o

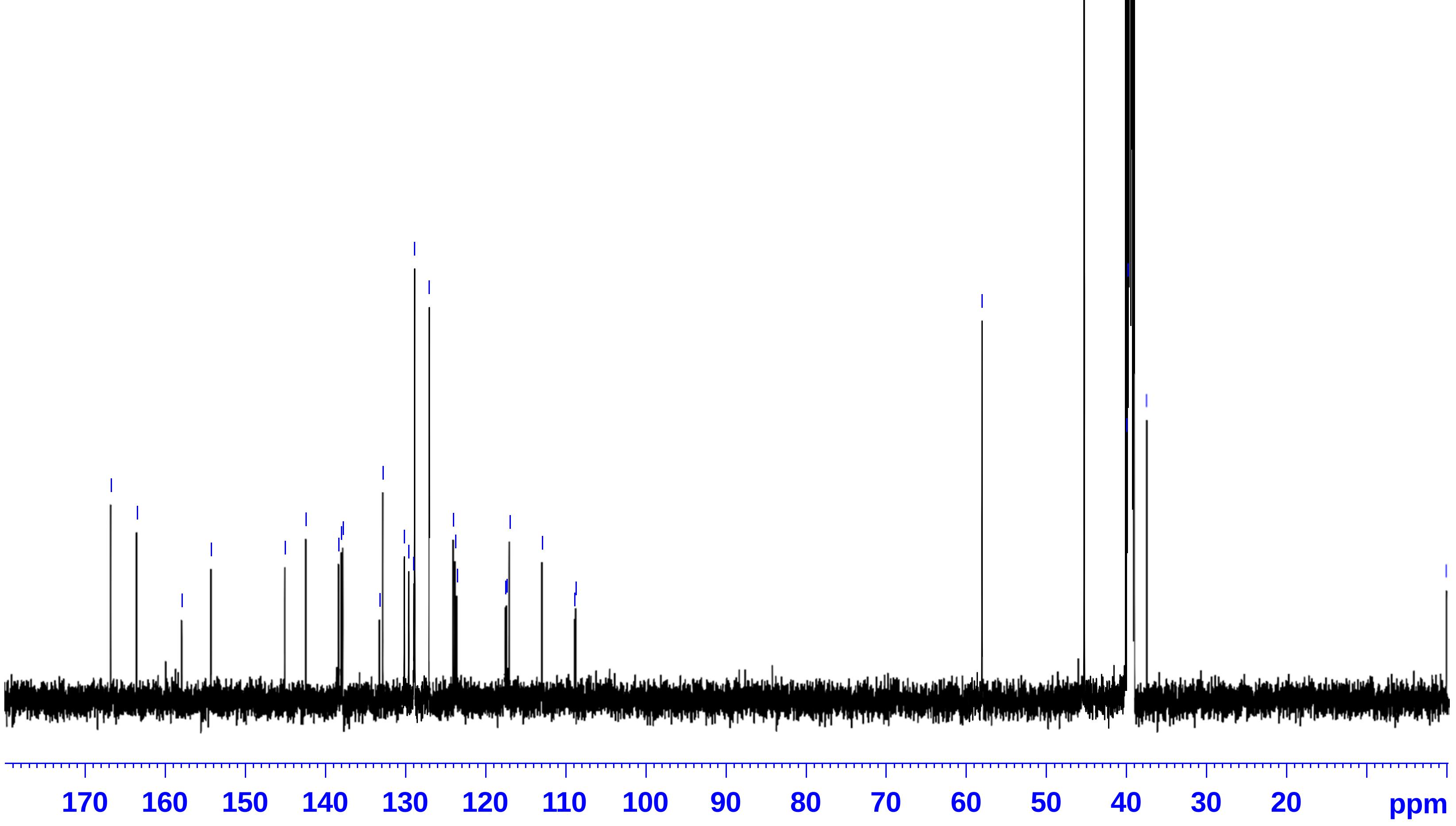

BRUKER

Current Data Parameters
NAME RK-III-31-26-13C
EXPNO 1
PROCNO 1

F2 - Acquisition Parameters
Date_ 20120611
Time 16.32
INSTRUM spect
PROBHD 5 mm CPTXI 1H-
PULPROG zgpg30
TD 65536
SOLVENT DMSO
NS 698
DS 4
SWH 30303.031 Hz
FIDRES 0.462388 Hz
AQ 1.0813940 sec
RG 23170.5
DW 16.500 usec
DE 6.50 usec
TE 298.0 K
D1 2.00000000 sec
D11 0.03000000 sec
TD0 1

======== CHANNEL f1 ========
NUC1 13C
P1 14.50 usec
PL1 -4.00 dB
PL1W 41.39080048 W
SFO1 125.7703638 MHz

======== CHANNEL f2 ========
CPDPRG2 waltz16
NUC2 1H
PCPD2 80.00 usec
PL2 -3.50 dB
PL12 13.50 dB
PL13 8.00 dB
PL2W 4.10509157 W
PL12W 0.08190735 W
PL13W 0.29061824 W
SFO2 500.1320005 MHz

F2 - Processing parameters
SI 32768
SF 125.7578482 MHz
WDW EM
SSB 0
LB 1.00 Hz
GB 0
PC 1.40

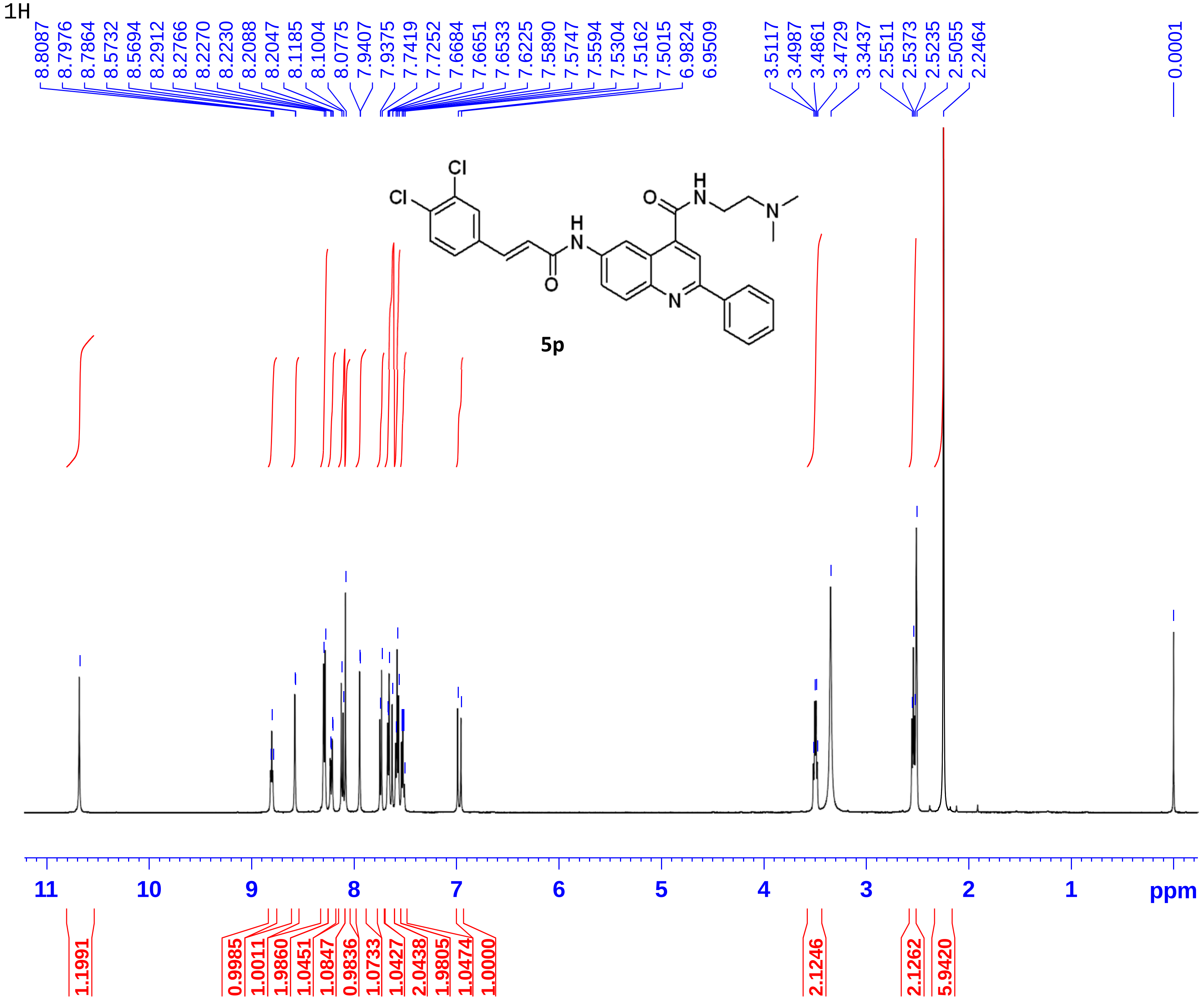

BRUKER

Current Data Parameters
NAME RK-III-44-23
EXPNO 1
PROCNO 1

F2 - Acquisition Parameters
Date_ 20120611
Time 17.11
INSTRUM spect
PROBHD 5 mm CPTXI 1H-
PULPROG zg
TD 32768
SOLVENT DMSO
NS 1
DS 0
SWH 7002.801 Hz
FIDRES 0.213709 Hz
AQ 2.3396852 sec
RG 18
DW 71.400 usec
DE 6.50 usec
TE 298.2 K
D1 2.00000000 sec
TD0 1

======== CHANNEL f1 ========
NUC1 1H
P1 9.25 usec
PL1 -1.00 dB
PL1W 2.30846262 W
SFO1 500.1323476 MHz

F2 - Processing parameters
SI 32768
SF 500.1300017 MHz
WDW no
SSB 0
LB 0 Hz
GB 0
PC 1.00

13C

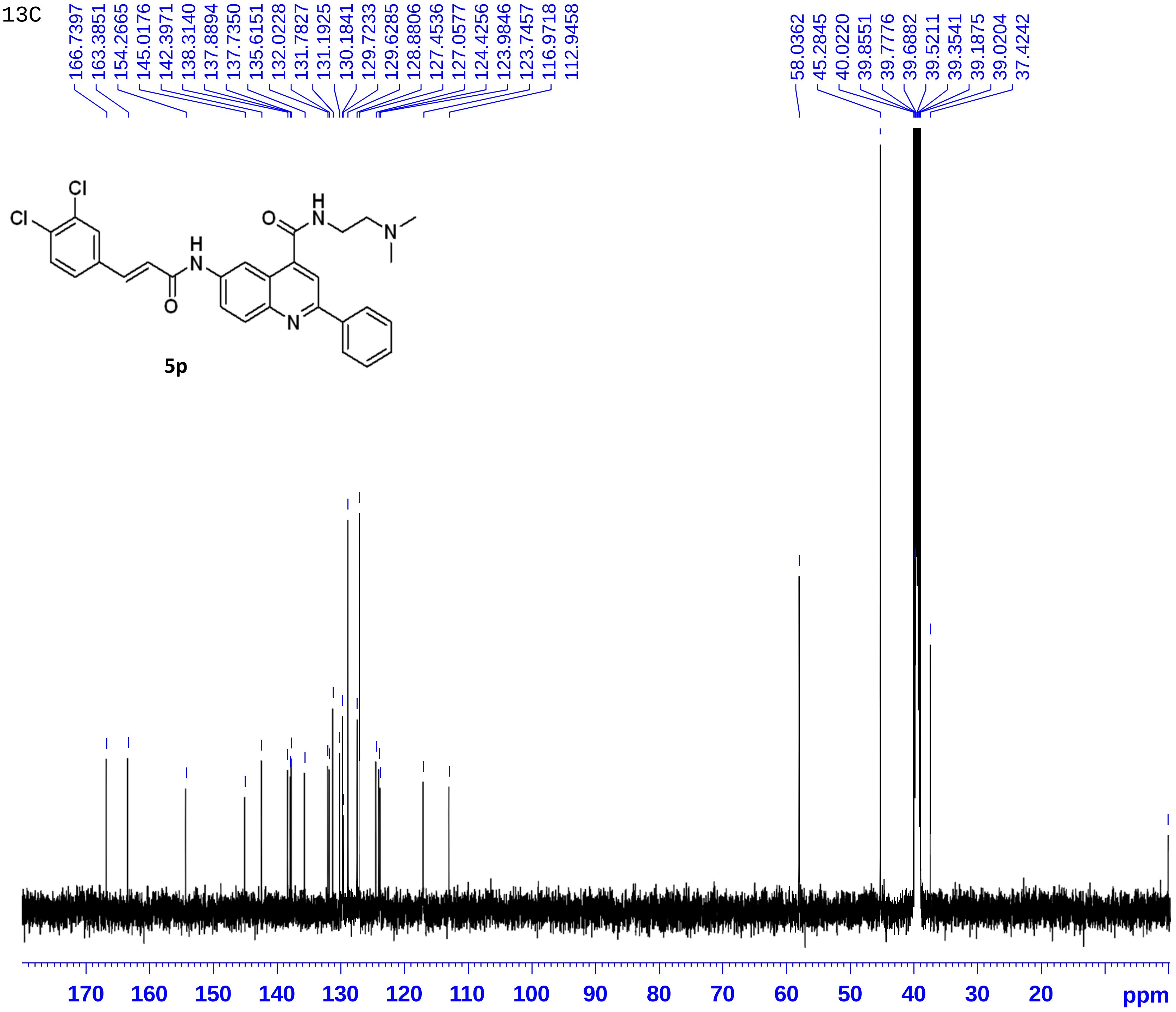

Current Data Parameters
NAME RK-III-44-23-13C
EXPNO 1
PROCNO 1

F2 - Acquisition Parameters
Date_ 20120611
Time 17.14
INSTRUM spect
PROBHD 5 mm CPTXI 1H-
PULPROG zgpg30
TD 65536
SOLVENT DMSO
NS 430
DS 4
SWH 30303.031 Hz
FIDRES 0.462388 Hz
AQ 1.0813940 sec
RG 23170.5
DW 16.500 usec
DE 6.50 usec
TE 298.0 K
D1 2.00000000 sec
D11 0.03000000 sec
TD0 1

======== CHANNEL f1 ========
NUC1 13C
P1 14.50 usec
PL1 -4.00 dB
PL1W 41.39080048 W
SFO1 125.7703638 MHz

======== CHANNEL f2 ========
CPDPRG2 waltz16
NUC2 1H
PCPD2 80.00 usec
PL2 -3.50 dB
PL12 13.50 dB
PL13 8.00 dB
PL2W 4.10509157 W
PL12W 0.08190735 W
PL13W 0.29061824 W
SFO2 500.1320005 MHz

F2 - Processing parameters
SI 32768
SF 125.7578482 MHz
WDW EM
SSB 0
LB 1.00 Hz
GB 0
PC 1.40

1H

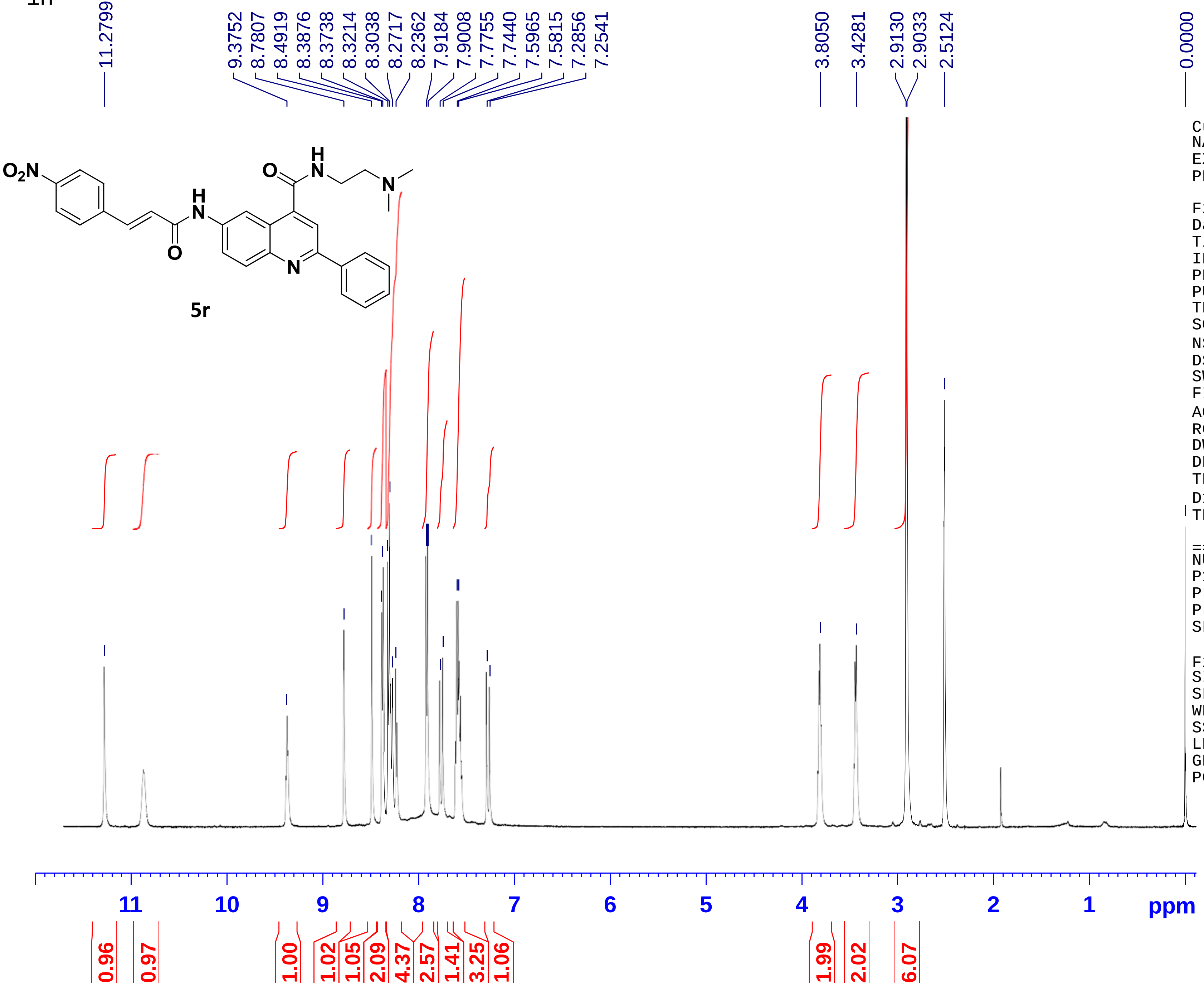

Current Data Parameters
NAME RK-II-167-29
EXPNO 1
PROCNO 1

F2 - Acquisition Parameters
Date_ 20110718
Time 16.11
INSTRUM spect
PROBHD 5 mm CPQNP 1H/
PULPROG zg
TD 32768
SOLVENT DMSO
NS 1
DS 0
SWH 7002.801 Hz
FIDRES 0.213709 Hz
AQ 2.3396852 sec
RG 18
DW 71.400 usec
DE 6.50 usec
TE 293.0 K
D1 2.00000000 sec
TD0 1

======== CHANNEL f1 ========
NUC1 1H
P1 9.25 usec
PL1 -1.00 dB
PL1W 2.30846262 W
SFO1 500.1323476 MHz

F2 - Processing parameters
SI 32768
SF 500.1299933 MHz
WDW no
SSB 0
LB 0 Hz
GB 0
PC 1.00

13C

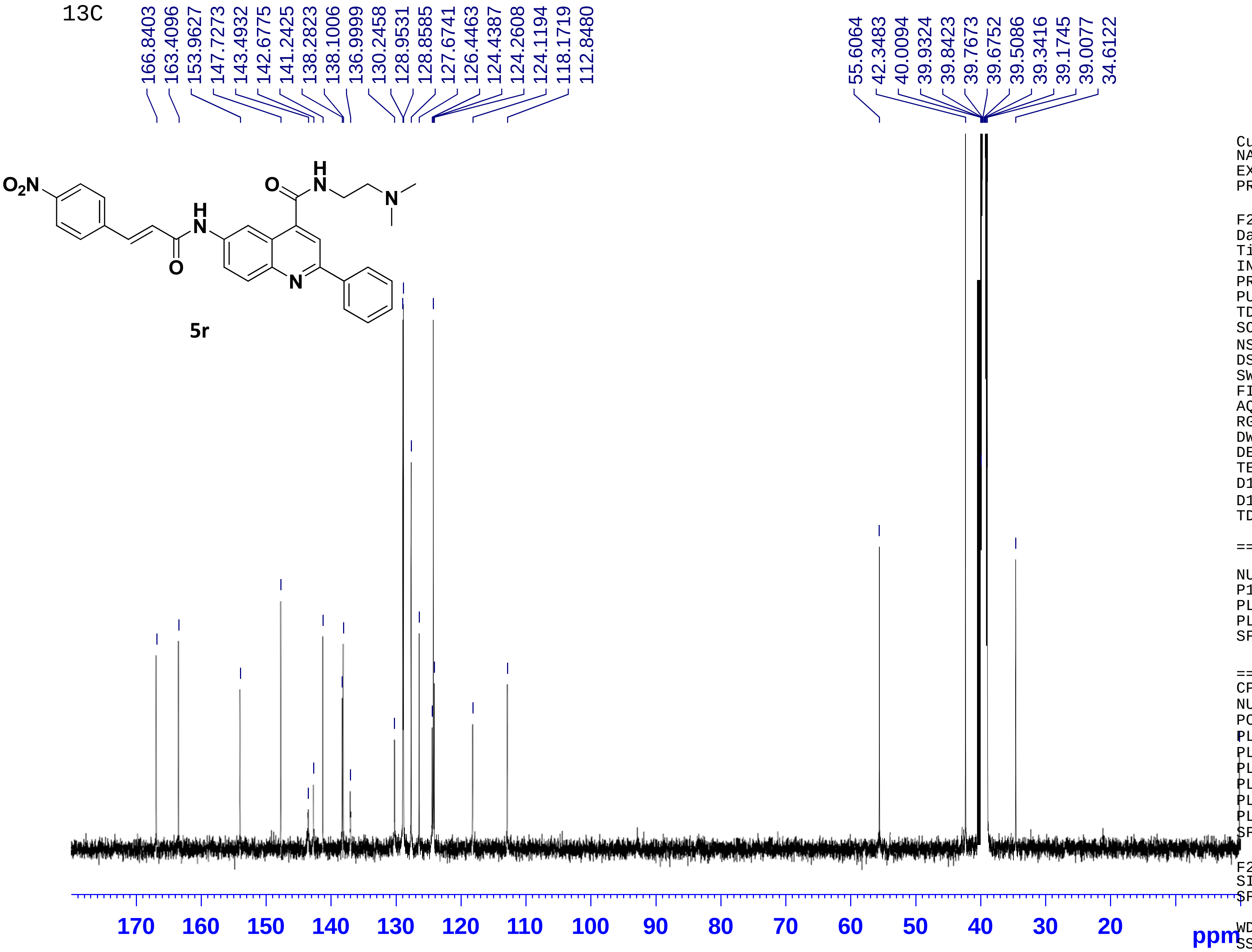

Current Data Parameters
NAME RK-II-167-29-13C
EXPNO 2
PROCNO 1

F2 - Acquisition Parameters
Date_ 20110718
Time 16.15
INSTRUM spect
PROBHD 5 mm CPQNP 1H/
PULPROG zgpg30
TD 65536
SOLVENT DMSO
NS 208
DS 4
SWH 30303.031 Hz
FIDRES 0.462388 Hz
AQ 1.0813940 sec
RG 6502
DW 16.500 usec
DE 6.50 usec
TE 293.0 K
D1 2.00000000 sec
D11 0.03000000 sec
TD0 1

======== CHANNEL f1 ========
NUC1 13C
P1 10.10 usec
PL1 -4.00 dB
PL1W 41.39080048 W
SFO1 125.7703638 MHz

======== CHANNEL f2 ========
CPDPRG2 waltz16
NUC2 1H
PCPD2 80.00 usec
PL2 -5.00 dB
PL12 9.50 dB
PL13 12.50 dB
PL2W 5.79859591 W
PL12W 0.20574196 W
PL13W 0.10311524 W
SFO2 500.1320005 MHz

F2 - Processing parameters
SI 32768
SF 125.7578418 MHz
WDW EM
SSB 0
LB 1.00 Hz
GB 0
PC 1.40
